# Perovskite Oxides Toward Oxygen Evolution Reaction: Intellectual Design Strategies, Properties and Perspectives

**DOI:** 10.1007/s41918-023-00209-2

**Published:** 2024-04-04

**Authors:** Lin-Bo Liu, Chenxing Yi, Hong-Cheng Mi, Song Lin Zhang, Xian-Zhu Fu, Jing-Li Luo, Subiao Liu

**Affiliations:** 1 School of Minerals Processing and Bioengineering, Central South University, Changsha, 410083 Hunan China; 2 Institute of Materials Research and Engineering, Agency for Science, Technology and Research, 2 Fusionopolis Way, Innovis, #08-03, Singapore, 138634 Singapore; 3 College of Materials Science and Engineering, Shenzhen University, Shenzhen, 518000 China; 4 Department of Chemical and Materials Engineering, University of Alberta, Edmonton, AB T6G 1H9 Canada

**Keywords:** Perovskite oxides, Oxygen evolution reaction, Synthetic modulation, Doping, Surface engineering, Structure mutation, Hybrids

## Abstract

Developing electrochemical energy storage and conversion devices (e.g., water splitting, regenerative fuel cells and rechargeable metal-air batteries) driven by intermittent renewable energy sources holds a great potential to facilitate global energy transition and alleviate the associated environmental issues. However, the involved kinetically sluggish oxygen evolution reaction (OER) severely limits the entire reaction efficiency, thus designing high-performance materials toward efficient OER is of prime significance to remove this obstacle. Among various materials, cost-effective perovskite oxides have drawn particular attention due to their desirable catalytic activity, excellent stability and large reserves. To date, substantial efforts have been dedicated with varying degrees of success to promoting OER on perovskite oxides, which have generated multiple reviews from various perspectives, e.g., electronic structure modulation and heteroatom doping and various applications. Nonetheless, the reviews that comprehensively and systematically focus on the latest intellectual design strategies of perovskite oxides toward efficient OER are quite limited. To bridge the gap, this review thus emphatically concentrates on this very topic with broader coverages, more comparative discussions and deeper insights into the synthetic modulation, doping, surface engineering, structure mutation and hybrids. More specifically, this review elucidates, in details, the underlying causality between the being-tuned physiochemical properties [e.g., electronic structure, metal–oxygen (M–O) bonding configuration, adsorption capacity of oxygenated species and electrical conductivity] of the intellectually designed perovskite oxides and the resulting OER performances, coupled with perspectives and potential challenges on future research. It is our sincere hope for this review to provide the scientific community with more insights for developing advanced perovskite oxides with high OER catalytic efficiency and further stimulate more exciting applications.

**Graphical Abstract:**

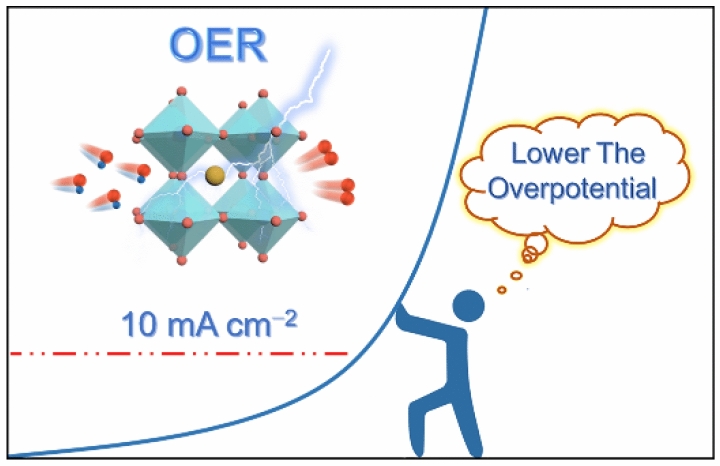

## Introduction

The continuously increasing energy demand is bound to stimulate the excessive exploitation and utilization of fossil fuels, inevitably accelerating ${\mathrm{CO}}_{2}$ accumulation in the atmosphere and further aggravating global warming and the associated environmental issues [1, 2]. The International Energy Outlook 2016 (IEO2016) predicted that global ${\mathrm{CO}}_{2}$ emissions would reach a record-breaking level of 43.2 billion tons by the year of 2040 [3]. Therefore, it is imperative to reduce CO_2_ emissions by developing highly efficient energy conversion and storage systems (e.g., electrolyzers, batteries and fuel cells) driven by renewable energies (e.g., wind, solar and geothermal energies), since they are considered as an effective, sustainable and environmentally friendly approach to addressing these issues from the source so as to achieve the global dual goals of “carbon emissions peak” and “carbon neutrality” [4–7]. Among them, the modular, compact, flexible and scalable electrochemical energy conversion and storage devices (e.g., ${\mathrm{H}}_{2}{\mathrm{O}}$ splitting, ${\mathrm{N}}_{2}$ and ${\mathrm{CO}}_{2}$ electroreduction), especially when feasibly integrated with renewable power sources, have drawn particular attentions since the whole process is controlled by electrochemical potential at ambient temperature and pressure [8, 9]. However, the coupling oxygen evolution reaction (OER) in alkaline solution [Eq. (1)] or acidic solution [Eq. (2)] at anode compartment normally determines the reaction rate because of their sluggish kinetics involving O–H breakage, O=O formation and complex proton-coupled electron transfer.

1 $${\mathrm{4OH}}^{-} { } \to {\text{ O}}_{{2}} { } + {\text{ 2H}}_{{2}} {\text{O }} + {\text{ 4e}}^{-}$$

2 $${\mathrm{2H}}_{{2}} {\text{O }} \to {\text{ O}}_{{2}} { } + {\text{ 4H}}^{ + } { } + {\text{ 4e}}^{-}$$

**Fig. 1:**
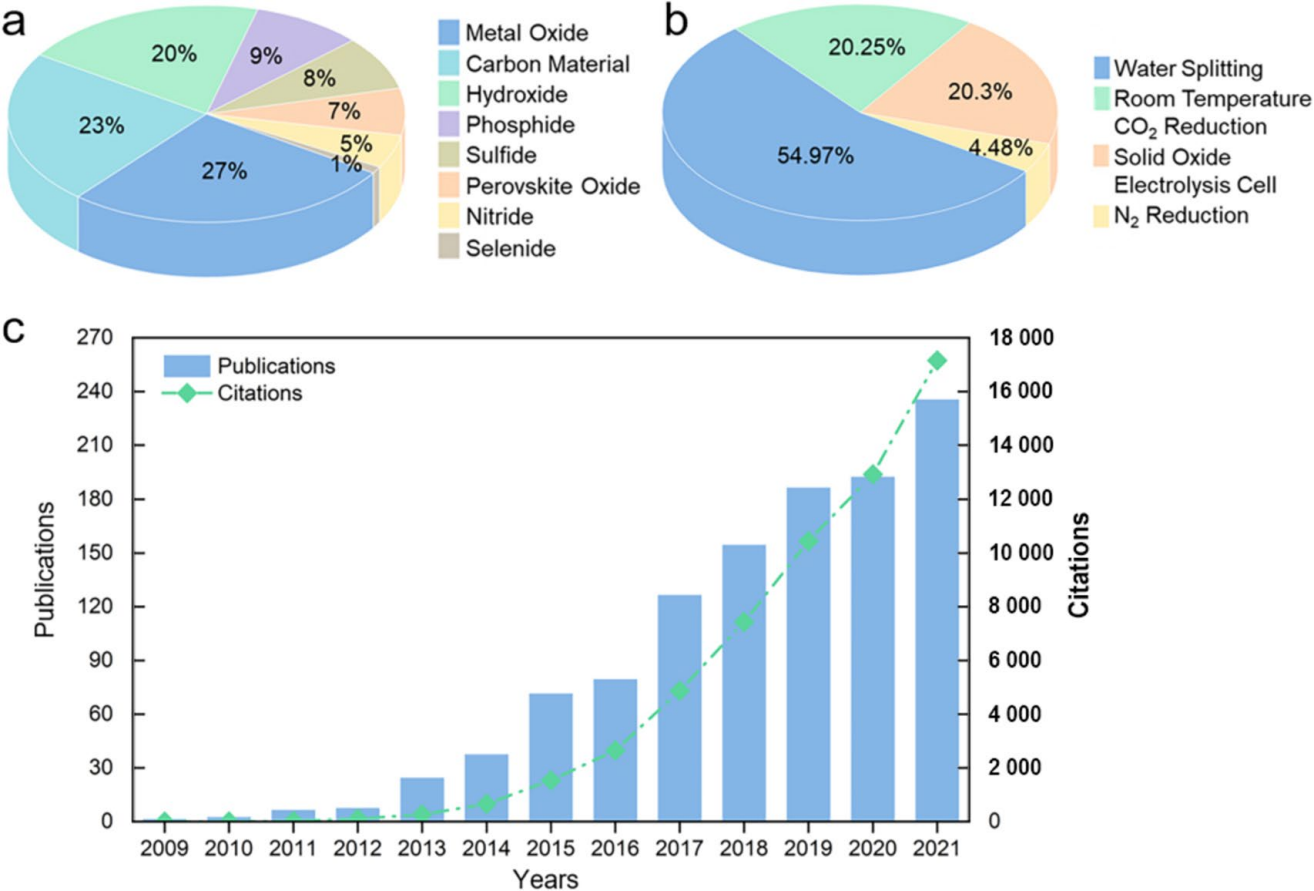
Statistics of previous studies on OER (*source*: Web of Science, 2022–09). The proportions of (**a**) different materials and **b** perovskite oxides applied in various electrolysis. **c** The number of publications and citations by searching the keywords of “oxygen evolution reaction” and “perovskites” from 2009 to 2021

The energy barriers built up in these reactions are measured as overpotential (*η*), i.e., the gap between the applied potential and the thermodynamic potential of 1.23 V vs. reversible hydrogen electrode (RHE) at 25 °C and 101.325kPa, and the *η* must be overcome to afford a practicably usable current density (*j*, e.g., 10 mA cm^−2^). Apparently, a high value of *η* would seriously limit the reaction and conversion efficiencies of the entire system [10–12]. This thus highlights the importance of highly active and stable materials capable of efficiently driving OER with comparably lower *η* toward the ultimate goal of closing the anthropogenic carbon cycle [13, 14]. In the past decades, precious and rare metal oxides (e.g., ${\mathrm{Ir}}{\mathrm{O}}_{2}$ and ${\mathrm{Ru}}{\mathrm{O}}_{2}$) have been widely recognized as the benchmark materials for effectively accelerating OER, but their scarcity, high cost and poor stability hinder their large-scale commercial utilizations [15, 16]. To this end, substantial efforts have been devoted to developing high-performance yet cost-effective alternatives with varying degrees of success, including but not limited to the transition metal oxides/hydroxides [17–19], sulfides [20, 21], phosphides [22, 23], carbon-based materials [24–26] and various composites [27, 28] (Fig. 1a).

**Fig. 2:**
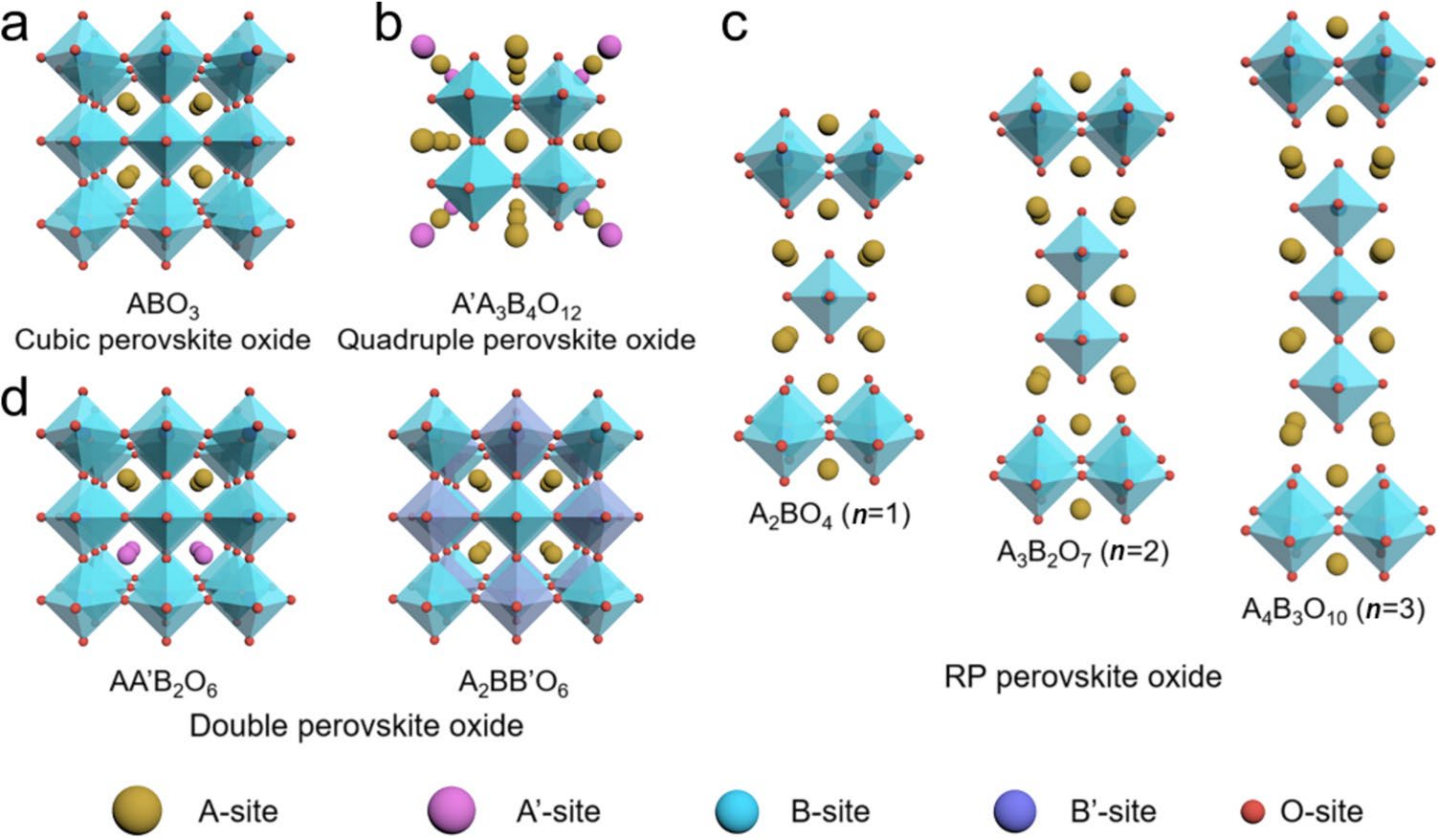
Typical crystal structures of different types of perovskite oxides: **a** Cubic (${\mathrm{AB}}{\mathrm{O}}_{3}$), **b** Quadruple (${\mathrm{A}}^{\prime}{\mathrm{A}}_{3}{{\mathrm{B}}}_{4}{{\mathrm{O}}}_{12}$), **c** A-site ordered (${\mathrm{AA}}^{\prime}{\mathrm{B}}_{2}{{\mathrm{O}}}_{6}$) and B-site ordered (${\mathrm{A}}_{2}{\mathrm{BB}}^{\prime}{\mathrm{O}}_{6}$) as well as **d** RP (${\mathrm{A}}_{{{n}}+ \mathrm{1} }{\mathrm{B}}_{{n}}{{\mathrm{O}}}_{{3}{{n}}+ \mathrm{1} }$, *n* = 1, 2 and 3) perovskite oxides

Of particular note, cubic perovskite oxides with a formula of ${\mathrm{AB}}{\mathrm{O}}_{3}$, where A is a rare or alkaline earth metal ion coordinated with 12-fold O and B is a transition metal ion coordinated with 6-fold O, have attracted much research interest in recent years, since they hold the very promising potential due to their intrinsically high electroactivity, large reserves and low-cost [13, 29] (Fig. 1b, c). Moreover, their variable composition and flexible crystal structures enable perovskite oxides to have more application possibilities [30]. Particularly, the derivatives such as the layered double perovskite oxides [31, 32], Ruddlesden-Popper (RP) perovskite oxides [33, 34] and quadruple perovskite oxides [35, 36] have also been extensively studied thanks to their desirable intrinsic catalytic activities (Fig. 2). Still, the challenges remain to be overcome prior to their commercial utilizations, including their limited specific surface area insufficient catalytic activities, poor ionic and electrical conductivities to afford a high *η* to achieve an applicable *j* (e.g., 10 mA cm^−2^). So far, many intellectual design strategies of perovskite oxides have been proposed aiming to effectively enhance their OER performances with varying degrees of success.

**Fig. 3:**
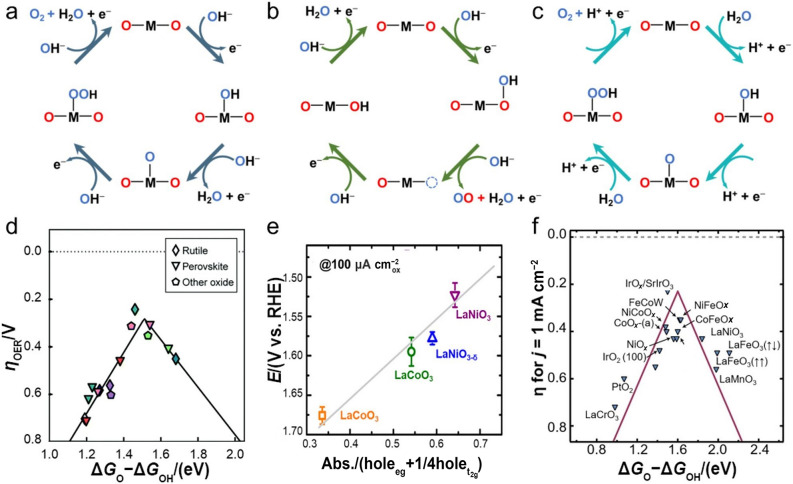
**a** AEM in alkaline media. **b** LOM in alkaline media. **c** OER mechanism in acidic media. **d** Volcano tendencies of OER activity versus ($\Delta G_{{\mathrm{*O}}} {{ {-} }}\Delta G_{{\mathrm{*OH}}}$). Adapted with permission from Ref. [38]. Copyright © 2016, Springer Nature. **e** The relation between OER activity and M−O covalency of perovskite oxides. Adapted with permission from Ref. [43]. Copyright © 2014, American Chemical society. **f** Volcano-type plot between *η*@*j* = 1 mA cm^−2^ and ($\Delta G_{{\mathrm{*O}}} {{ {-} }}\Delta G_{{\mathrm{*OH}}}$) over various catalysts for OER. Adapted with permission from Ref. [44]. Copyright © 2017, American Association for the Advancement of Science

It is worth noting that the underlying OER mechanisms still remain controversial and need further explorations. Currently, the widely recognized and accepted mechanism for OER on the surface of perovskite oxides is adsorbate evolution mechanism (AEM), which involves the proton-coupled $4 {\mathrm{e}}^{-}$ transfers on the exterior metal centers (M) and the final ${\mathrm{O}}_{2}$ generation from ${\mathrm{OH}}^{-}$ [37], as described in Fig. 3a. More specifically, ${\mathrm{OH}}^{-}$ first absorbs on M to form M–OH, and then the absorbed *OH deprotonates to obtain *O, which intermediately combines with another ${\mathrm{OH}}^{-}$ to deliver *OOH, and subsequently deprotonates again to generate *OO and evolves ${\mathrm{O}}_{2}$ (Note: * represents the adsorption of oxygenated intermediates on M). To rationalize the AEM, the reaction free energy (Δ*G*) difference between $\Delta G_{{\mathrm{*O}}}$ and $\Delta G_{{\mathrm{*OH}}}$ (i.e., $\Delta G_{{\mathrm{*O}}} {{ {-} }}\Delta G_{{\mathrm{*OH}}}$) is established as a descriptor to evaluate the appropriateness of certain perovskite oxides for OER by virtue of the rate-determining step of *OH deprotonation during OER. Currently, the volcano tendency between *η* and ($\Delta G_{{\mathrm{*O}}} {{ {-} }}\Delta G_{{\mathrm{*OH}}}$) toward OER has been well recognized by the associated fields (Fig. 3d), where a ($\Delta G_{{\mathrm{*O}}} {{ {-} }}\Delta G_{{\mathrm{*OH}}}$) value of 1.6 eV points to an optimal OER catalytic activity [38], which is consistent with the Sabatier principle that the transition metal cations cannot bind the adsorbed oxygen intermediates too strongly nor too weakly [39]. Subsequently, Suntivich and co-workers proposed another representative OER descriptor regarding intrinsic catalytic activity, which suggested that the B-site cations ${\mathrm{e}}_{\mathrm{g}}$ orbital filling needs to be close to 1.0. This is verified by the previously reported ${\mathrm{Ba}}_{{{0}{\mathrm{.5}}}} {\mathrm{Sr}}_{{{0}{\mathrm{.5}}}} {\mathrm{Co}}_{{{0}{\mathrm{.8}}}} {\mathrm{Fe}}_{{{0}{\mathrm{.2}}}} {\mathrm{O}}_{{{3}{-}\delta }}$ (BSCF) with an ${\mathrm{e}}_{\mathrm{g}}$ of 1.2, which showed a comparably higher OER catalytic activity than all of the other perovskite oxides and commercial ${\mathrm{Ir}}{\mathrm{O}}_{2}$ [16]. However, the typical AEM has been challenged by the recent lattice oxygen oxidation mechanism (LOM), where the lattice oxygen from the perovskite oxide surface is assumed as the active sites that can directly participate in OER [40], as illustrated in Fig. 3b. In details, lattice oxygen, coupled with adsorbed oxygen (i.e., ${\mathrm{OH}}^{-}$), directly forms M–**O**OH (Note: **O** represents the lattice oxygen), where *OH deprotonation and lattice oxygen oxidation contribute to the formations of ${\mathrm{O}}_{2}$ and an oxygen vacancy and subsequently ${\mathrm{OH}}^{-}$ from the electrolyte refills the oxygen vacancies and further undergoes deprotonation to regenerate the lattice oxygen site. This claims the importance of lattice oxygen for the transformation of key intermediates toward LOM [41]. Moreover, this also indicates that tuning the metal–oxygen (M–O) covalency of perovskite oxides plays a crucial role in LOM, since the lattice oxygen and the associated M–**O**OH directly participate in the final ${\mathrm{O}}_{2}$ generation, while the key *OH deprotonation determines the reaction rate. More importantly, perovskite oxides undergoing LOM involve a distinct reaction pathway for O–O coupling and could bypass the inherent limitation caused by the adsorption energy scaling relationship between *OH and *OOH (i.e., a constant difference of ≈ 3.2 eV between $\Delta G_{{\mathrm{*OOH}}}$ and $\Delta G_{{\mathrm{*OH}}}$) toward AEM. This enables a much better OER catalytic activity and a much lower Tafel slope [42]. As demonstrated by the Shao-Horn group, where stoichiometric ${\mathrm{LaNi}}{\mathrm{O}}_{3}$ with enhanced M–O covalency showed an optimal OER performance (Fig. 3e) [43]. It is worth noting that the underlying OER mechanism in acid media is similar to the AEM in alkaline media, indicating that the descriptor of ${(}\Delta G_{{\mathrm{O*}}} {{ {-} }}\Delta G_{{\mathrm{HO*}}} {)}$ is also applicable to evaluate the suitability of perovskite oxides in acid media (Fig. 3f) [44]. The remarkable difference lies in the ${\mathrm{OH}}^{-}$ from the ${\mathrm{H}}_{{2}} {\mathrm{O}}$ dissociation process ${\mathrm{(H}}_{2} {\text{O }} \to {\text{ H}}^{ + } { } + {\text{ OH}}^{-} {)}$ rather than the electrolyte itself (Fig. 3c) [45], and the extra energy barrier caused by ${\mathrm{H}}_{2}{\mathrm{O}}$ dissociation in acidic media would slow the OER kinetics to some extent as compared to that in alkaline media.

Despite the existence of debates, the different underlying mechanisms raise the two key concerns, i.e., what decides the difference and how it works? To clarify both issues and rationalize what has been achieved, a comprehensive and systematic discussion and a deep understanding on the latest intellectual design strategies of perovskite oxides for OER and the associated intrinsic relationships of design strategies–properties–activities are thus highly in need. Through screening the literature, previously published reviews almost only focus on one specific design strategy of perovskite oxides for OER or different types of materials for various applications. Although OER is partially mentioned, a comprehensive coverage on intellectual design strategies reflecting the fast-growing progress of this field is not found. Moreover, the design strategies mentioned within these most relevant recent reviews collectively fall short of fully covering all aspects of the subject since they only deal with some individual parts of this field. This creates an actual need to write a new review including not only the most recent findings and discoveries with one step further but also the critical analyses and key research highlights that connect the missing parts in previous reviews and fill the gaps in the knowledge base. The importance of an adequate and comprehensive understanding of the induced physiochemical and structural properties resulting from various design strategies cannot be more emphasized since all the associated properties are normally interlinked and affected each other. In this regard, the relatively simple description of any individual design strategy cannot comprehensively elucidate some underlining interactions and inter-platform phenomena. Therefore, the deep understandings of the interlinked relationships among all the design strategies summarized in our review, the associated being-tuned physiochemical and structural properties and OER activities of perovskite oxides are indispensable.

**Fig. 4:**
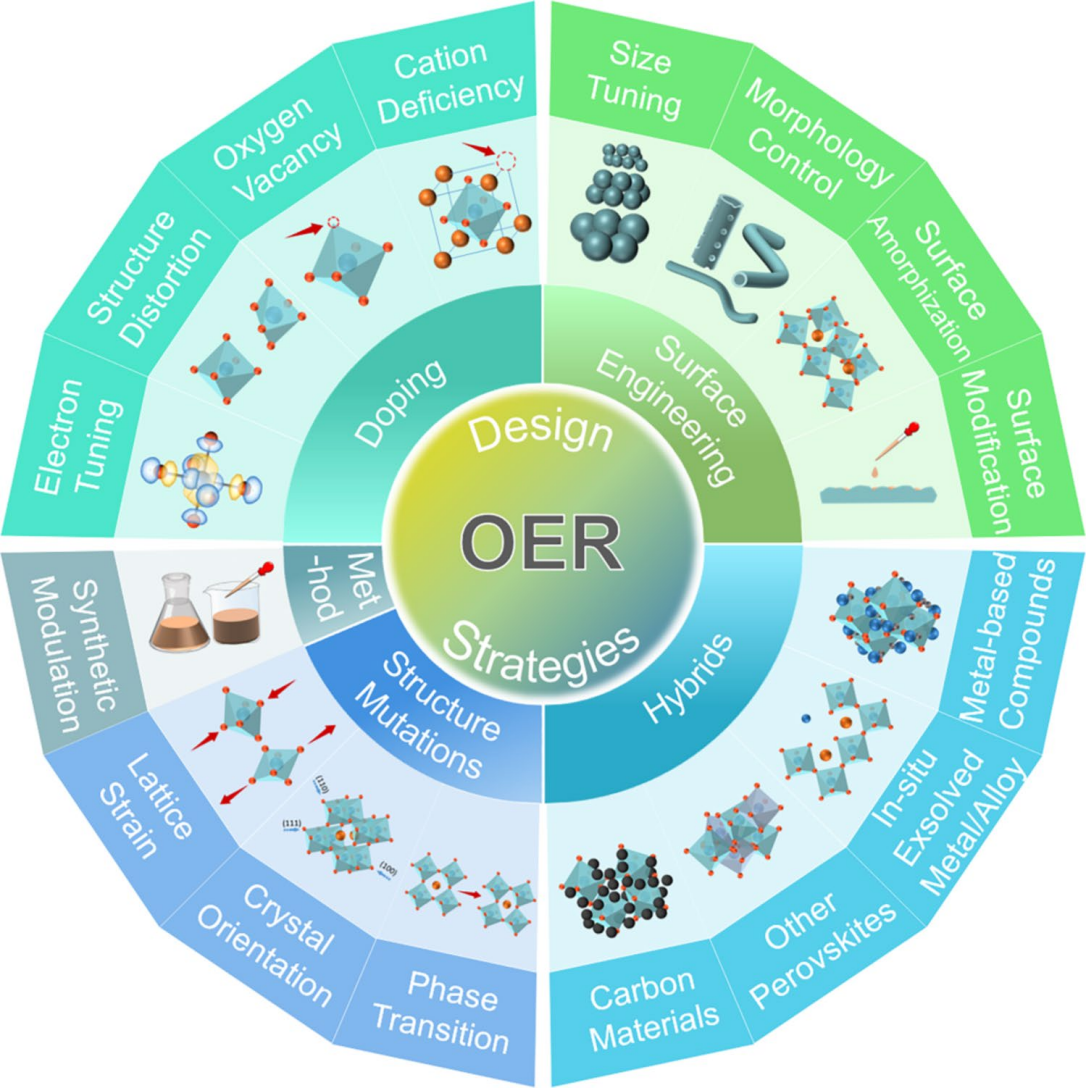
Schematic illustration of intellectual design strategies of perovskite oxides toward efficient OER

To bridge this gap, we systematically and comprehensively summarized the intellectual design strategies mainly from five aspects: synthetic modulation, doping, surface engineering, structure mutation and hybrids (Fig. 4). Also included in this review are the corresponding theoretical computations, e.g., density functional theory (DFT) calculations of Gibbs free energies, adsorption energies and density of states (DOS) as well as charge densities and transition states, to quantitatively illustrate the underlying relationship between the intrinsic physical and/or chemical properties of perovskite oxides and the associated OER performances, with an emphasis on the following factors for each aspect, including but not limited to the electronic distribution, structure distortion, oxygen vacancy, surface defect and the synergistic effect of heterogeneous composite, etc. More importantly, our perspectives on developing perovskite oxides as the alternative materials capable of driving highly efficient and stable OER and the potential challenges are also presented at last. Clearly, this review is in the position to fulfill that need by emphatically concentrating on this very topic with much broader coverage, more comparative discussions and deeper insights into synthetic modulation, doping, surface engineering, structure mutation and hybrid. We sincerely hope that this review would collectively provide more fundamental insights of these “wonder materials” and inspire more exciting applications of this growing family of perovskite oxides due to their wide versatility, diversity and flexibility. This review would also provide better understandings and more general guidelines for broader nanomaterial research.

## Synthetic Modulation

One specific perovskite oxide with the same composition but synthesized by different synthesis methods could sometimes display different properties and the associated properties-activities relationships. Generally, perovskite oxides are ${\mathrm{ABO}}_{3}$-type of oxides that can be regarded as a compound of two metal oxides (i.e., ${\mathrm{A}}{\mathrm{O}}_{{x}}$ and ${\mathrm{BO}}_{{{3}{-}x}}$), where A is commonly a rare-earth or alkaline earth element and B is commonly a transition metal, allowing them to be synthesized by solid-state reaction through mechanically ball milling the associated metal oxides and then annealing at elevated temperature. For example, ${\mathrm{La}}_{{{1}{-}x}} {\mathrm{Sr}}_{x} {\mathrm{CoO}}_{{3}}$ is synthesized by integrating stoichiometric amounts of ${\mathrm{La}}_{2}{{\mathrm{O}}}_{3}$, ${\mathrm{SrC}}{\mathrm{O}}_{3}$ and ${\mathrm{Co}}_{3}{{\mathrm{O}}}_{4}$ with mild grinding and subsequent calcination at 1 100 °C in air for 24 h [46]. However, perovskite oxides synthesized via this synthetic route generally possess relatively large particle sizes and even form impurities, which are not favorable for improving the OER catalytic activity. Additionally, many other strategies, including solution combustion [47], sol-gel [48], Pechini [49], co-precipitation [50, 51], hydrothermal/solvothermal [52, 53] and molten salt methods [54, 55] have also been developed to prepare perovskite oxides. In fact, various synthesis methods have their own pros and cons for the target products, mainly depending upon the specific applications and the practical demands. For example, solution combustion tends to form the perovskite oxides with small particle sizes through dissolving metal precursors and glycine/citric acid in deionized water and then annealing at an elevated temperature for certain length of time, while sol-gel method follows the similar procedures and has been widely used for preparing perovskite oxides since high purity and uniform nanostructure could be easily achieved. In a typical sol-gel synthesis of BSCF, stoichiometric metal precursors, i.e., ${\mathrm{Ba}}{\mathrm{(N}{\mathrm{O}}_{3}\mathrm{)}}_{2}$, ${\mathrm{Sr}}{\mathrm{(}{\mathrm{NO}}_{3}\mathrm{)}}_{2}$, ${\mathrm{Co(NO}}_{{3}} {)}_{{2}} \cdot {\text{ 6H}}_{{2}} {\mathrm{O}}$ and ${\mathrm{Fe(NO}}_{{3}} {)}_{{3}} \cdot {\text{ 9H}}_{{2}} {\mathrm{O}}$ are first dissolved in deionized water under continuous stirring, a combined solution of ethylenediamine tetraacetic acid (EDTA) and ${\mathrm{NH}}_{3}{{\mathrm{H}}}_{2}{\mathrm{O}}$ is then added, followed by the addition of citric acid. The mixture is expected to form an organic resin under continuous stirring at an elevated temperature, and after a subsequent calcination, the gel forms the target BSCF [56]. Accordingly, the pechini method is similar to the sol-gel one, except that the transition metal ions are trapped in a polymer gel. In fact, the co-precipitation method has also been used to prepare perovskite oxides based on the precipitation reaction between organic bases and metal precursors, e.g., ${\mathrm{LaAl}}{\mathrm{O}}_{3}$ and ${\mathrm{La}}_{0.8}{\mathrm{Sr}}_{0.2}{\mathrm{Mn}}{\mathrm{O}}_{3}$ [50, 51]. However, impurities commonly exist due to the quite different hydrolysis rates of various multi-metal salts. In addition, hydrothermal and solvothermal methods are able to control the size, crystallinity, morphology of perovskite oxides by adjusting the experimental parameters (e.g., reaction temperature, time, solvents, surfactants or metal precursors) through chemical reactions of metal precursors in aqueous solution at elevated temperature and pressure. The molten-salt method can synthesize perovskite oxides by controlling the rapid growth of metal precursors in fused salt, which could tune the particle sizes and microstructures through modulating the amounts and types of salts, reaction time, temperature and cooling rates [57]. In addition to the direct synthesis of perovskite oxides, other strategies by means of high-technique instruments have also been extensively investigated and have drawn increasing attention. For example, electrospinning is a facile and versatile method for synthesizing perovskite oxides with specific nanostructures and large specific surface areas [58, 59], and pulsed laser deposition could easily form thin films of perovskite oxides [60, 61], while flame spraying obtains a comparably smaller particle size of perovskite oxides [62].

However, various synthesis strategies deliver different physicochemical properties of perovskite oxides, which greatly affect their OER catalytic performances. Comparatively, the BSCF synthesized by solid-state reaction displayed a higher conductivity and a better OER catalytic activity than those by solution combustion and sol-gel method [63]. However, a converse study on ${\mathrm{La}}_{{{0}{\mathrm{.4}}}} {\mathrm{Sr}}_{{{0}{\mathrm{.6}}}} {\mathrm{Co}}_{{{0}{\mathrm{.7}}}} {\mathrm{Fe}}_{{{0}{\mathrm{.2}}}} {\mathrm{Nb}}_{{{0}{\mathrm{.1}}}} {\mathrm{O}}_{{{3}{-}\delta }}$ found that sol-gel method could enable a larger specific surface area, a superior ${\mathrm{e}}_{\mathrm{g}}$ filling and more oxygen vacancy content as compared to solid-state reaction, with the final results of having a higher catalytic activity and a better stability for OER [64]. Moreover, Bail et al. reported a novel and economic method for perovskite oxides crystallization at a low temperature, the as prepared ${\mathrm{LaFe}}{\mathrm{O}}_{3}$ nanoparticles (NPs) display an outstanding OER performance benefitting from the presence of rich oxygen vacancies and active surface species [65]. Despite the existence of debatable conclusions, the influences of different synthetic methods have been demonstrated to affect the OER performances of perovskite oxides to some extent, thus a reasonable and feasible selection of synthetic strategies is highly in need to well modulate the properties like particle size, morphology and crystallinity.

## Doping

The doping strategy, including cation (A- and/or B-site) doping and anion (O-site) doping, has been widely and intensively investigated in many fields, e.g., solid oxide cells [66, 67], oxygen separation and membrane reactor [68, 69], all of which could also provide fundamental and practical guidance in designing highly active and stable perovskite oxides that can enable desirable OER. Throughout the literatures, the doping strategy mainly affects four aspects of perovskite oxides, i.e., electron tuning, structure distortion, oxygen vacancy and cation deficiency, all of which could tailor the electroactive sites, the reaction path, the electronic structure and the band structure for OER to some extent.

### Electron Tuning

**Fig. 5:**
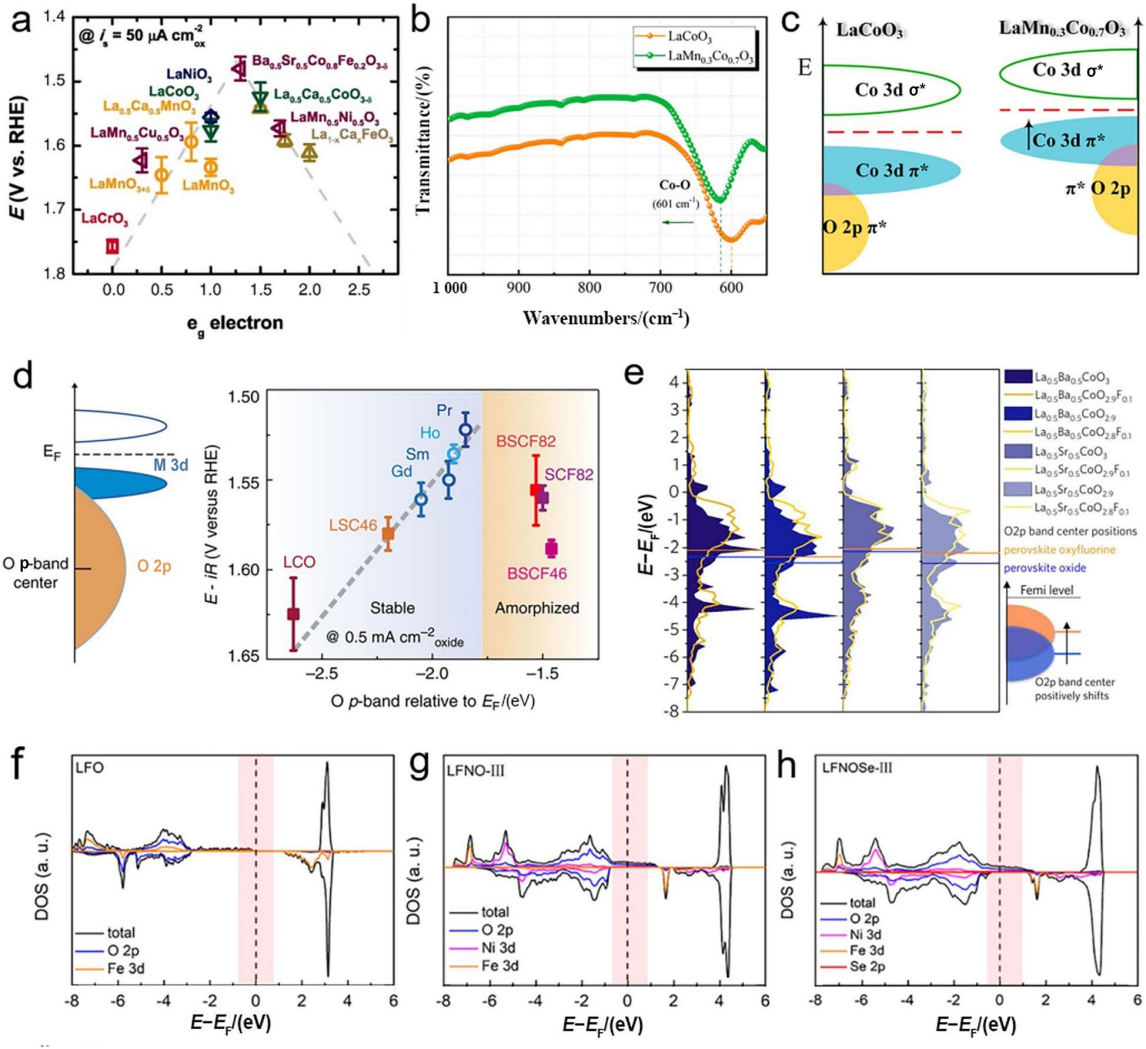
**a** The relation between potentials at 50 ${\upmu \mathrm{A}}\, {\mathrm{cm}}^{{{-}2}}$ and ${\mathrm{e}}_{\mathrm{g}}$ fillings of B-site cations in various perovskite oxides. Adapted with permission from Ref. [16]. Copyright © 2011, American Association for the Advancement of Science. **b** FTIR spectra of ${\mathrm{LaCo}}{\mathrm{O}}_{3}$ and ${\mathrm{La}}{\mathrm{Mn}}_{0.3}{\mathrm{Co}}_{0.7}{\mathrm{O}}_{3}$. **c** Schematic diagram of uplifted O 2p band of ${\mathrm{La}}{\mathrm{Mn}}_{0.3}{\mathrm{Co}}_{0.7}{\mathrm{O}}_{3}$. Adapted with permission from Ref. [74]. Copyright © 2020, American Chemical Society. **d** The relation between potentials at 0.5 mA cm^−2^ and O p-band relative to *E*_*f*_ (eV) on various perovskite oxides. Adapted with permission from Ref. [31]. Copyright © 2013, Springer Nature. **e** Projected DOS of O 2p bands for ${\mathrm{La}}_{{{0}{\mathrm{.5}}}} {\mathrm{Ba}}_{{{0}{\mathrm{.5}}}} {\mathrm{CoO}}_{{3{-}\delta }}$, ${\mathrm{La}}_{{{0}{\mathrm{.5}}}} {\mathrm{Ba}}_{{{0}{\mathrm{.5}}}} {\mathrm{CoO}}_{{{2}{\mathrm{.9}}{-}\delta }} {\mathrm{F}}_{{{0}{\mathrm{.1}}}}$, ${\mathrm{La}}_{{{0}{\mathrm{.5}}}} {\mathrm{Sr}}_{{{0}{\mathrm{.5}}}} {\mathrm{CoO}}_{{3{-}\delta }}$, and ${\mathrm{La}}_{{{0}{\mathrm{.5}}}} {\mathrm{Sr}}_{{{0}{\mathrm{.5}}}} {\mathrm{CoO}}_{{{2}{\mathrm{.9}}{-}\delta }} {\mathrm{F}}_{{{0}{\mathrm{.1}}}}$ (*δ* = 0 or 0.1). Adapted with permission from Ref. [81]. Copyright © 2018, Cell Press. f−h) DOS of ${\mathrm{LaFe}}{\mathrm{O}}_{3}$, ${\mathrm{La}}{\mathrm{Fe}}_{0.25}{\mathrm{Ni}}_{0.75}{\mathrm{O}}_{3}$ and Se-doped ${\mathrm{La}}{\mathrm{Fe}}_{0.25}{\mathrm{Ni}}_{0.75}{\mathrm{O}}_{3}$. Adapted with permission from Ref. [84]. Copyright © 2020, American Chemical Society

Through doping foreign elements into A- and/or B-sites, the electronic structure of perovskite oxides could be well tuned to deliver the optimal catalytic performance. In an octahedral ${\mathrm{B}}{\mathrm{O}}_{6}$ coordination of perovskite oxide, transition metal d orbital hybridizes with O 2p orbital in an octahedral crystal field and contributes to five d orbitals (i.e., $\mathrm{d}_{{x^{2} {-}y^{2} }}$, $\mathrm{d}_{{z^{2} }}$, $\mathrm{d}_{xy}$, $\mathrm{d}_{yz}$, $\mathrm{d}_{xz}$), which are classified into two groups, namely, σ orbital (known as ${\mathrm{e}}_{\mathrm{g}}$ orbital) with higher energy (i.e., ${\mathrm{d}}_{{x^{2} {-}y^{2} }}$ and ${\mathrm{d}}_{{{z}}^{2}}$) and π orbital (known as ${\mathrm{t}}_{\mathrm{2g}}$ orbital) with lower energy (i.e., ${\mathrm{d}}_{{xy}}$, ${\mathrm{d}}_{{yz}}$ and ${\mathrm{d}}_{{xz}}$) [13, 15]. Importantly, the ${\mathrm{e}}_{\mathrm{g}}$ orbitals of surface transition metal cations participate in bonding with adsorbed oxygen intermediates, and its electron occupation would remarkably affect the catalytic activity of perovskite oxides, which is well recognized as a volcano-shape diagram in Fig. 5a [16]. Moreover, electrons of d orbitals in transition metal cations have a complicated arrangement due to their varying oxidation and spin states [70]. For example, Du et al. demonstrated that the Sr-doped ${\mathrm{La}}_{0.5}{\mathrm{Sr}}_{0.5}{\mathrm{NiO}}_{3}$ epitaxial films with enhanced hybridization of Ni 3d–O 2p and accelerated charge transfer ability exhibited the improved OER catalytic activity [71], while Chen et al. confirmed that the Ni 3d–O 2p covalency of ${\mathrm{La}}_{{0}\mathrm{.9}}{\mathrm{Sn}}_{0.1}{\mathrm{NiO}}_{3}$ became stronger than that of the pristine ${\mathrm{LaNi}}{\mathrm{O}}_{3}$, together with the enhanced electron transfer ability through Sn doping [72]. Nevertheless, doping Ce in ${\mathrm{La}}_{0.7}{\mathrm{Ce}}_{0.3}{\mathrm{Co}}{\mathrm{O}}_{3}$ could modulate the charge density of the active Co atoms and consequently increase the electrical conductivity and the final OER performance [73]. Compared with the abovementioned doping strategy in A-site, doping in B-site holds the potential to accommodate a wider range of dopants and allows a more flexible tuning of electronic structure. For instance, Yin et al. found that the O 2p band center was closer to the Fermi level, and the covalency of Co–O bonds was enhanced by doping appropriate amounts of Mn in ${\mathrm{LaCo}}{\mathrm{O}}_{3}$ (Fig. 5b, c) [74], both of which subsequently promoted the charge transfer between the surface metal cations and reaction intermediates and also improved OER catalytic activity; this was further confirmed by the dual-doping of Sc and Nb in ${\mathrm{SrSc}}_{{{0}{\mathrm{.025}}}} {\mathrm{Nb}}_{{{0}{\mathrm{.025}}}} {\mathrm{Co}}_{{{0}{\mathrm{.95}}}} {\mathrm{O}}_{{{3}{-}\delta }}$, i.e., the dual-doping could increase the electron occupancy near the Fermi level and improve the ${\mathrm{OH}}^{-}$ adsorption capability [75]. Likewise, by doping nonmetallic P at B-site, it was verified that the high-valence ${\mathrm{P}}^{5 + }$ in the as-prepared ${\mathrm{Sr(Co}}_{{{0}{\mathrm{.8}}}} {\mathrm{Fe}}_{{{0}{\mathrm{.2}}}} {)}_{{{0}{\mathrm{.95}}}} {\mathrm{P}}_{{{0}{\mathrm{.05}}}} {\mathrm{O}}_{{3{-}\delta }}$ not only could reduce the valence state of Co cation and promote the electron transfer, but also could induce the increase of oxygen vacancy content [76].

Besides the cubic perovskite oxides, the layered perovskite oxides have also been extensively studied for OER due to their unique layered crystal structure along with a multitude of attractive physical and chemical properties, such as high diffusion and surface-exchange coefficients [77, 78]. Shao-Horn et al. compared A-site ordered double perovskite oxides of (${\mathrm{Ln}}_{{{0}{\mathrm{.5}}}} {\mathrm{Ba}}_{{{0}{\mathrm{.5}}}} {\mathrm{)CoO}}_{{3{-}\delta }}$ (Ln = Pr, Sm, Gd, and Ho) with the facially amorphized BSCF in strong alkaline solution and found that such double perovskite oxides, especially (${\mathrm{Pr}}_{{{0}{\mathrm{.5}}}} {\mathrm{Ba}}_{{{0}{\mathrm{.5}}}} {\mathrm{)CoO}}_{{3{-}\delta }}$, showed superb activity and stability originating from their optimal O p-band center position that is neither too close nor too far relative to the Fermi level (Fig. 5d) [31]. This is further confirmed by double perovskite oxide of ${\mathrm{PrBa}}_{{{0}{\mathrm{.5}}}} {\mathrm{Sr}}_{{{0}{\mathrm{.5}}}} {\mathrm{Co}}_{{{1}{\mathrm{.5}}}} {\mathrm{Fe}}_{{{0}{\mathrm{.5}}}} {\mathrm{O}}_{5 + \delta }$, where partially doping Sr and Fe rendered O p-band center to locate at a more proper position relative to the Fermi level [79]. Accordingly, with the benefit of an appropriate O p-band center relative to the Fermi level and a strong ${\mathrm{OH}}^{-}$ adsorption and ${\mathrm{O}}_{{2}}$ desorption abilities, RP-type perovskite oxide of ${\mathrm{LaSr}}_{{3}} {\mathrm{Co}}_{m} {\mathrm{Fe}}_{{{3} - m}} {\mathrm{O}}_{{10{-}\delta }}$ with an optimal Co doping ratio of *m* = 1.5 exhibited an excellent OER catalytic activity [33]. Moreover, selective doping Ni in ${\mathrm{La}}_{{{0}{\mathrm{.5}}}} {\mathrm{Sr}}_{{{1}{\mathrm{.5}}}} {\mathrm{Ni}}_{{{1}{-}x}} {\mathrm{Fe}}_{x} {\mathrm{O}}_{{{4} + \delta }}$ could also tune the overlap between the Ni and Fe 3d bands and the O 2p band, as reported by the Robin group [80].

Apart from the conventional cation doping strategy at A- and/or B-sites of perovskite oxides, partially substituting O-site by anion (e.g., F, Cl and Se) also serves as an effective strategy to optimize OER performance. Initially, doping F in ${\mathrm{La}}_{{{0}{\mathrm{.5}}}} {\mathrm{Ba}}_{{{0}{\mathrm{.25}}}} {\mathrm{Sr}}_{{{0}{\mathrm{.25}}}} {\mathrm{CoO}}_{{2.9{-}\delta }} {\mathrm{F}}_{{{0}{\mathrm{.1}}}}$ was studied, and the results showed that partial substitution of O by F could rise the O ${\mathrm{p}}$-band center and improve the electrical conductivity (Fig. 5e) [81]. Subsequently, an optimal Cl doping ratio of 0.1 in ${\mathrm{LaFeO}}_{{{3}{-}x{-}\delta }} {\mathrm{Cl}}_{x}$, i.e., ${\mathrm{LaFeO}}_{{{2}{\mathrm{.9}}{-}\delta }} {\mathrm{Cl}}_{{{0}{\mathrm{.1}}}}$, achieved the best OER performance on account of the increased electrical conductivity and the enhanced Fe–O covalency [82]. Besides the halogen group (VIIA) elements (e.g., F and Cl), the chalcogen group (VIA) elements (e.g., S and Se) were also investigated to explore their influences on electronic structure [83]. Wang et al. employed DFT calculations to rationalize the quite low *η* of only 287 mV on Se-doped ${\mathrm{La}}{\mathrm{Fe}}_{0.25}{\mathrm{Ni}}_{0.75}{\mathrm{O}}_{3}$ at 10 mA cm^−2^ in 1.0 mol L^−1^ KOH solution and concluded that Se doping uplifted the O 2p band center (Fig. 5f–h), which thus enhanced the conductivity and optimized the adsorption of intermediates [84]. Such observations provide solid and persuasive evidence that partially doping pristine perovskite oxides in cation (A- and/or B-sites) and anion (O-site) paves an effective and attractive way to remarkably tailor OER electrocatalysis. This also highlights the importance of exploring the cation and anion doping toward OER.

### Structure Distortion

**Fig. 6:**
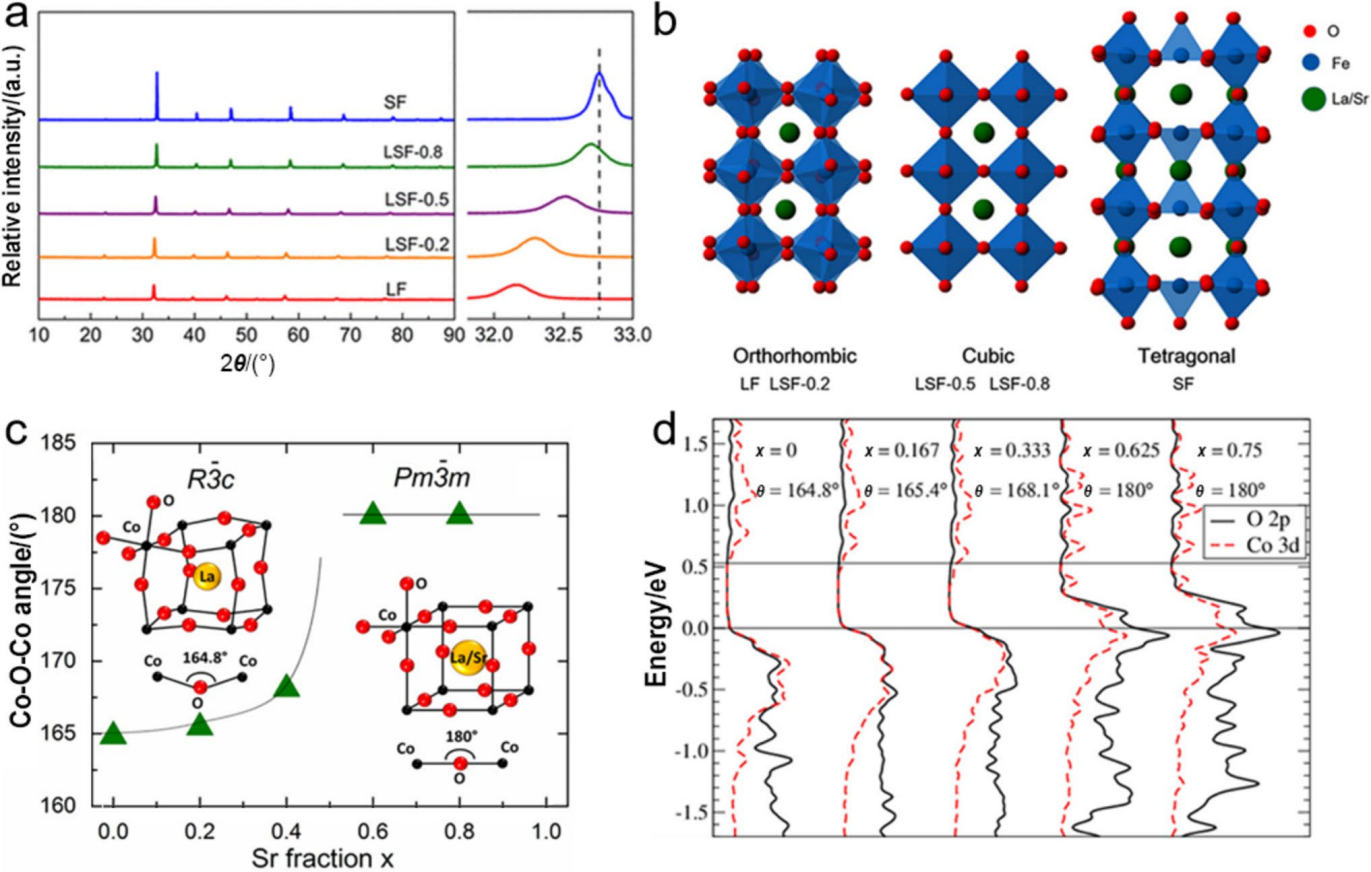
**a** X-ray diffraction patterns of LF with different Sr doping ratios. **b** Crystal structures of orthorhombic LF and LSF-0.2, cubic LSF-0.5 and LSF-0.8, and tetragonal SF. Adapted with permission from Ref. [87]. Copyright © 2018, American Chemical Society. **c** The relation between Co−O−Co angle and Sr fraction. **d** PDOS of ${\mathrm{La}}_{{{1}{-}x}} {\mathrm{Sr}}_{x} {\mathrm{CoO}}_{{3}}$ versus Co−O−Co angle and Sr content. Adapted with permission from Ref. [88]. Copyright © 2015, American Chemical Society

The strategy of doping foreign elements in an ideal cubic perovskite oxide at O-, A- and/or B-sites normally enables a structure distortion, which inevitably deteriorates the optimal length ratio of A–O bond/B–O bond of $\sqrt{2}$. However, the structure usually allows a certain degree of mismatch of ionic radius, which is often described by the Goldschmidt tolerance factor in Eq. (3) [85]:

3 $${{t}}\text{ = }\frac{{{r}}_{\mathrm{A}} \, \mathrm{+} \, {{r}}_{\mathrm{O}}}{\sqrt{2}\mathrm{(}{{r}}_{\mathrm{B}} \, \mathrm{+} \, {{r}}_{\mathrm{O}}\mathrm{)}}$$

where ${\mathrm{r}}_{\mathrm{A}}$, ${\mathrm{r}}_{\mathrm{B}}$ and ${\mathrm{r}}_{\mathrm{O}}$ are the ionic radii of A, B and O ions, respectively. Generally, $t \approx 1$ represents an ideal cubic symmetry, whereas $0.7 \leqslant t < 1$ means a structure distortion and a compensation for the cation size mismatch, and $t > 1$ stands for the formation of other phases instead of perovskite oxides [86]. In this regard, Shao et al. systematically investigated the effects of crystal structural distortion of several Sr-incorporated ${\mathrm{La}}_{{{1}{-}x}} {\mathrm{Sr}}_{x} {\mathrm{FeO}}_{{{3}{-}\delta }}$ samples on OER performance and found that the lattice size, accompanied with the gradual transition of crystal structure, decreased with the increase of Sr content (Fig. 6a, b). The results revealed that despite the occurrence of a structural transition from orthorhombic to cubic perovskite oxide, the sample with a doping ratio of 0.8, i.e., ${\mathrm{La}}_{{{0}{\mathrm{.2}}}} {\mathrm{Sr}}_{{{0}{\mathrm{.8}}}} {\mathrm{FeO}}_{{3{-}\delta }}$ still enjoyed an acceptable structural distortion and demonstrated the best OER catalytic activity and stability [87]. A further study found that the Sr substitution in ${\mathrm{La}}_{{{1}{-}x}} {\mathrm{Sr}}_{x} {\mathrm{CoO}}_{{3}}$ led to a structure distortion and caused an increase of Co–O–Co angle from 164.83° in ${\mathrm{LaCo}}{\mathrm{O}}_{3}$ to 180° in ${\mathrm{La}}_{0.4}{\mathrm{Sr}}_{0.6}{\mathrm{Co}}{\mathrm{O}}_{3}$, which consequently resulted in a structural transition from a rhombohedral ($x \leqslant 0.4$) to a cubic ($x \geqslant 0.6$) structure (Fig. 6c) and an improved electrical conductivity benefiting from the increased overlap between the occupied O 2p valence band and the unoccupied Co 3d conduction band (Fig. 6d) [88]. This is further confirmed by Tanja and coworkers in that doping Ni in ${\mathrm{La}}_{{{0}{\mathrm{.7}}}} {\mathrm{Sr}}_{{{0}{\mathrm{.3}}}} {\mathrm{Fe}}_{{{0}{\mathrm{.6}}}} {\mathrm{Ni}}_{{{0}{\mathrm{.4}}}} {\mathrm{O}}_{{3{-}\delta }}$ enabled a structure distortion and as a result, formed a cubic symmetry structure with an improved electrical conductivity [89].

Particularly, when half of A- and/or B-sites cations in cubic ${\mathrm{ABO}}_{3}$ are replaced by other cations (i.e., ${\mathrm{AA}}^{\prime } {\mathrm{B}}_{{2}} {\mathrm{O}}_{{6}} {\text{, A}}_{{2}} {\mathrm{BB}}^{\prime } {\mathrm{O}}_{{6}} {\text{ and AA}}^{\prime } {\mathrm{BB}}^{\prime } {\mathrm{O}}_{{6}}$), the disordered or ordered double perovskite oxide structure with varying degrees of distortion will be formed on account of the different cation radii [90, 91]. For example, a lattice contraction was observed through doping Sr in ${\mathrm{PrBa}}_{{{1}{-}x}} {\mathrm{Sr}}_{x} {\mathrm{Co}}_{{2}} {\mathrm{O}}_{{{5} + \delta }}$, where an increased Co–O covalency and an accelerated electron transfer were obtained [92], which was further confirmed by doping Co in ${\mathrm{PrBaMn}}_{{2}} {\mathrm{O}}_{{{5} + \delta }}$ [93]. Accordingly, the Ciucci group reported that layered ${\mathrm{NdBa}}{\mathrm{Mn}}_{2}{{\mathrm{O}}}_{5.5}$ possessed a much higher distorted structure and exhibited higher electroactivity as compared to the cubic ${\mathrm{Nb}}_{{{0}{\mathrm{.5}}}} {\mathrm{Ba}}_{{{0}{\mathrm{.5}}}} {\mathrm{MnO}}_{{3{-}\delta }}$ and ${\mathrm{NdBaMn}}_{{2}} {\mathrm{O}}_{5.5 + \delta }$ [94]. More importantly, the vibronic superexchange of ${\mathrm{Ni}}^{3 + } {-}{\mathrm{O}}{-}{\mathrm{Mn}}^{3 + }$ in ${\mathrm{La}}_{2}{\mathrm{NiMn}}{\mathrm{O}}_{6}$ induced distortions of ${\mathrm{MnO}}_{6}$ and ${\mathrm{NiO}}_{6}$ octahedra, which could optimize the ${\mathrm{e}}_{\mathrm{g}}$ filling state and promote the formation of active species of Mn/Ni oxide/hydroxide on the surface of perovskite oxides, thus leading to a comparably high OER performance [95]. All the studies indicate that the crystal structure distortion derived from the doping strategy on perovskite oxides toward electrocatalysis also matters to some extent.

### Oxygen Vacancy

Upon doping A- and/or B-sites cations by dopants with low valence (e.g., Sr and Ba), an unbalanced electronegativity would be created on the whole lattice, which subsequently induces O atoms to be released from the perovskite oxide lattice to maintain charge neutrality and simultaneously form oxygen vacancies in the lattice, which also transforms the lattice from an octahedral structure of ${\mathrm{BO}}_{6}$ to a pyramidal structure of ${\mathrm{BO}}_{5}$. Practically, the oxygen vacancies concentration greatly depends on the degree of substitution and the employed dopants [96].

**Fig. 7:**
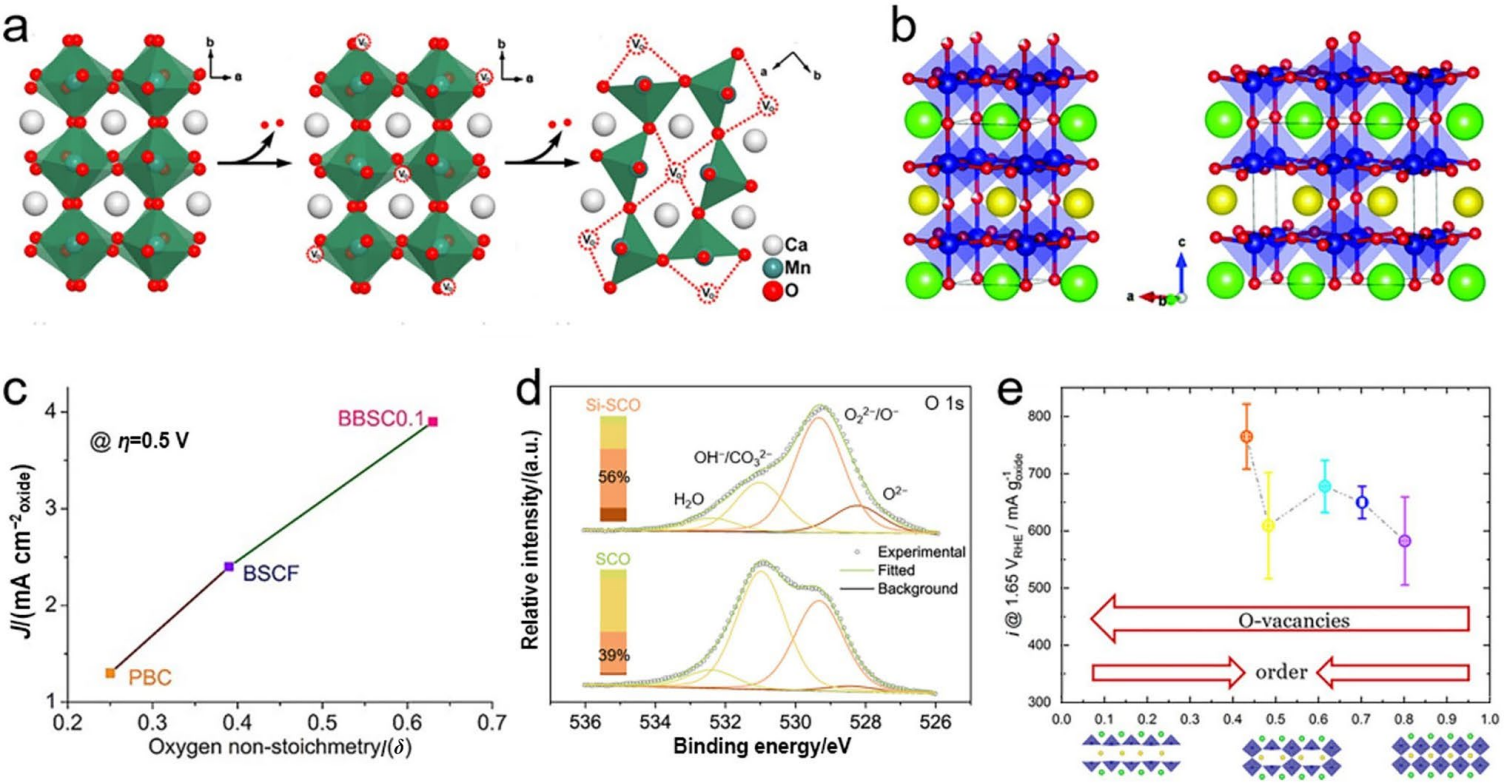
Crystal structures of **a** stoichiometric ${\mathrm{CaMn}}{\mathrm{O}}_{3}$ and oxygen-deficient ${\mathrm{CaMn}}{\mathrm{O}}_{2.76}$ and ${\mathrm{CaMn}}{\mathrm{O}}_{2.5}$, **b** ${\mathrm{PrBa}}{\mathrm{Co}}_{2}{{\mathrm{O}}}_{5.75}$ (left) and ${\mathrm{PrBa}}{\mathrm{Co}}_{2}{{\mathrm{O}}}_{5.5}$ (right). Adapted with permission from Ref. [97, 100]. Copyright © 2014, American Chemical Society; Copyright © 2019, The Royal Society of Chemistry. **c** *j* at *η* = 0.5 V against $\delta$ of PBC, BSCF and BBSC0.1. Adapted with permission from Ref. [102]. Copyright © 2017, Wiley–VCH. **d** O 1s XPS spectra of SCO and Si-SCO. Adapted with permission from Ref. [106]. Copyright © 2020, Springer Nature. **e** The relation between *i* at 1.65 V and $\delta$, and crystal structures of ${\mathrm{PrBaCo}}_{{2}} {\mathrm{O}}_{{{5} + \delta }}$ ($\delta$ = 1, 0.5 and 0). Adapted with permission from Ref. [107]. Copyright © 2021, Wiley–VCH

To get insights into the oxygen vacancy effect and the associated underlying mechanism for the enhanced OER performance, Chen and co-workers investigated a series of nonstoichiometric perovskite oxides of ${\mathrm{CaMnO}}_{{{3}{-}\delta }}$ (0 < $\delta \, \leqslant$   0.5) (Fig. 7a) and demonstrated that ${\mathrm{CaMnO}}_{{3{-}\delta }}$ with oxygen non-stoichiometry value ($\delta$) of 0.25 reached the highest electrical conductivity [97]. To specifically recognize the oxygen vacancy, Gui et al. integrated Sr doping with Ar plasma treatment to introduce rich oxygen vacancies into ${\mathrm{La}}{\mathrm{CoO}}_{3}$ [98], and it turned out that the ${\mathrm{La}}_{{{0}{\mathrm{.7}}}} {\mathrm{Sr}}_{{{0}{\mathrm{.3}}}} {\mathrm{CoO}}_{{{3}{-}\delta }}$ provided the most active sites and the best OER catalytic activity. Different from randomly distributed oxygen vacancies in cubic perovskite oxides, oxygen vacancies in layered perovskite oxides tend to localize in A–O layers due to the differences in ionic radii and polarizability between two A-site cations (Fig. 7b) [94, 99, 100]. Luo group reported that layered ${\mathrm{PrBa}}_{{{0}{\mathrm{.85}}}} {\mathrm{Ca}}_{{{0}{\mathrm{.15}}}} {\mathrm{MnFeO}}_{{{5} + \delta }}$ could provide long-range ordered oxygen vacancy channels and rich surface oxygen species [101], while Shao group verified that a higher $\delta$ in Bi-doped ${\mathrm{Ba}}_{{2}} {\mathrm{Bi}}_{{{0}{\mathrm{.1}}}} {\mathrm{Sc}}_{{{0}{\mathrm{.2}}}} {\mathrm{Co}}_{{{1}{\mathrm{.7}}}} {\mathrm{O}}_{{{6}{-}\delta }}$ enabled a faster diffusion rate of oxygen ions (Fig. 7c) [102]. A further study by Tavassol et al. confirmed that not only the oxygen vacancies content but also its specific chemical ordering in Ca-doped ${\mathrm{Sr}}_{{2{-}x}} {\mathrm{Ca}}_{x} {\mathrm{Fe}}_{{2}} {\mathrm{O}}_{{6{-}\delta }}$ could remarkably affect the OER performance [103]. However, it is worth noting that excessive oxygen vacancies in perovskite oxides could reversely decay the OER performance, as confirmed by the lower OER activity of ${\mathrm{PrBa}}{\mathrm{Mn}}_{2}{{\mathrm{O}}}_{{5}\mathrm{.5}}$ with a higher content of oxygen vacancies but lower ${\mathrm{e}}_{\mathrm{g}}$ orbital occupancy, higher electrical resistivity and weaker Co–O bond covalency than ${\mathrm{PrBa}}{\mathrm{Mn}}_{2}{{\mathrm{O}}}_{5.75}$ [100].

As previously stated on LOM, lattice oxygen directly participates in the formation of intermediates during OER, which was identified by ^18^O isotope detection [104]. Actually, the presence of oxygen vacancy has also been proposed to be beneficial to proceeding lattice OER (LOER) since that oxygen vacancies could facilitate the refilling of the surface lattice oxygen as it is consumed [105]. To demonstrate it, the Shao group doped different ratios of Si in ${\mathrm{SrCo}}_{{1{-}y}} {\mathrm{Si}}_{y} {\mathrm{O}}_{{{3}{-}\delta }}$ (*y* = 0.03, 0.05, 0.07 and 0.10) to increase the highly oxidative species (${\mathrm{O}}^{{2{-}}} {\mathrm{/O}}^{-}$) on the surface with several levels of surface oxygen vacancies concentration (Fig. 7d) and observed a strong pH dependence on OER activity, indicating the participation of lattice oxygen on Si-doped samples. Moreover, the one with a Si doping ratio of 0.05 displayed the fastest oxygen ion diffusion rate and the highest intrinsic activity, persuasively verifying the effectiveness of the increased oxygen vacancies content in enhancing LOER performance [106]. Coincidentally, Fabbri group achieved almost the similar conclusions that a moderate amount of oxygen vacancies could greatly increase the oxygen ion diffusion rate and consequently facilitate the LOER, whereas a high oxygen vacancy order would in turn reduce the oxygen diffusivity and lower the OER catalytic activity (Fig. 7e) [107]. Overall, surface oxygen vacancy plays a crucial role in modifying the physiochemical properties of perovskite oxides (e.g., electronic structure, electrical conductivity and oxygen ions diffusion rates) [108], which consequently tailor the adsorption and desorption capacities of oxygenated intermediates on the surface and the final OER performances.

### Cation Deficiency

**Fig. 8:**
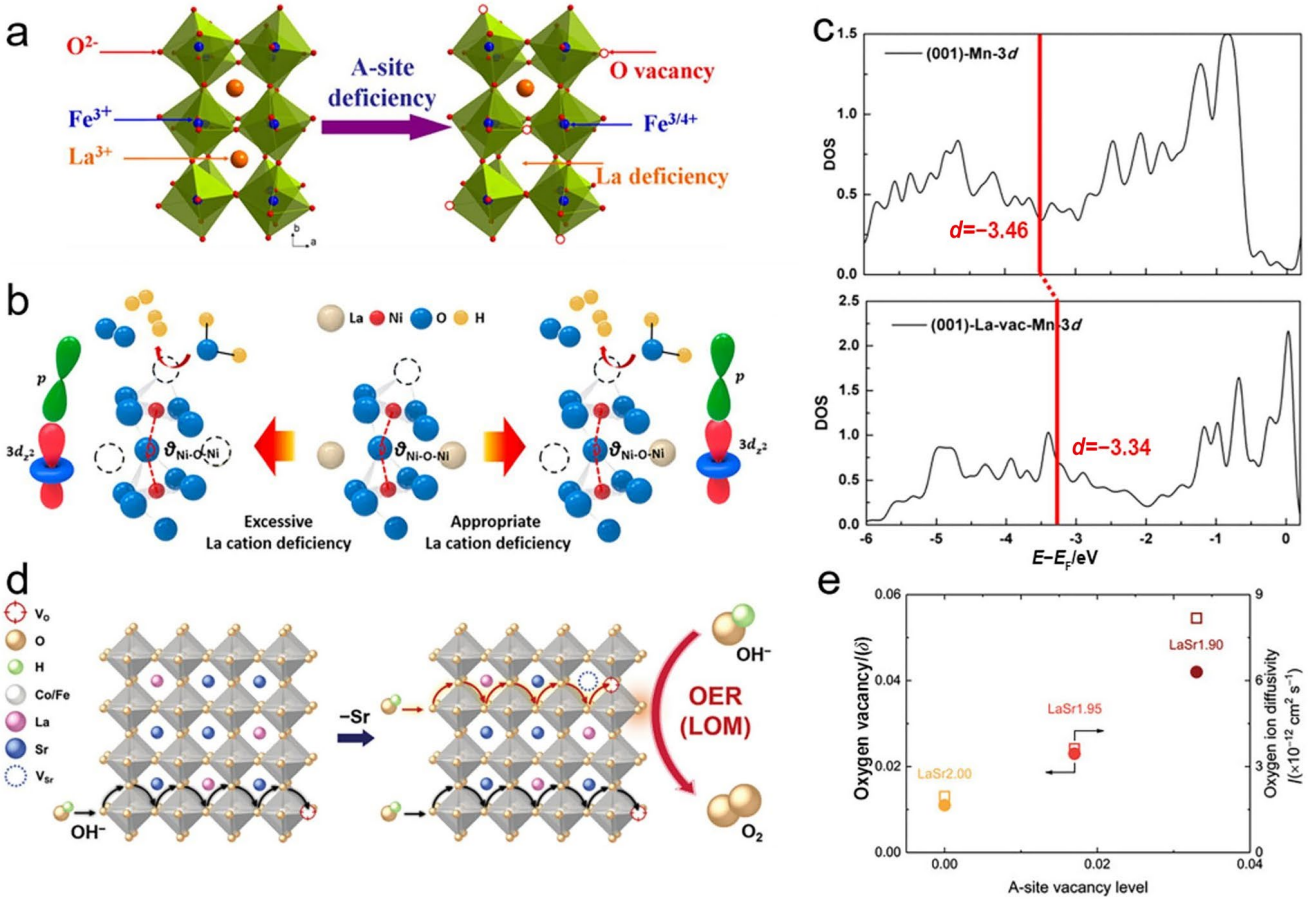
**a** The formation of oxygen vacancy and ${\mathrm{Fe}}^{4 + }$ cations in A-site deficient ${\mathrm{La}}_{{1{-}x}} {\mathrm{FeO}}_{{3}}$. Adapted with permission from Ref. [109]. Copyright © 2016, American Chemical Society. **b** The changes of Ni−O−Ni bond angle in excessive and appropriate La-deficient ${\mathrm{La}}_{{{1}{-}x}} {\mathrm{NiO}}_{{3}}$ films. Adapted with permission from Ref. [110]. Copyright © 2020, American Chemical Society. **c** The calculated d−band centers of ${\mathrm{LaMn}}{\mathrm{O}}_{3}$ and La-deficient ${\mathrm{La}}_{{{1}{-}\delta }} {\mathrm{MnO}}_{{3}}$. Adapted with permission from Ref. [113]. Copyright © 2020, Elsevier Ltd. **d** Schematic illustration of OER with a LOM pathway over Sr deficient perovskite oxide and **e** $\delta$ and oxygen ion diffusivity versus the A-site deficiency level. Adapted with permission from Ref. [114]. Copyright © 2020, Wiley–VCH

Introducing certain cation deficiency to break the stoichiometric A/B molar ratio and maintain its stability by distortion is another attractive strategy to significantly alter the physical and chemical properties of perovskite oxides. By far, most studies mainly focus on the A-site deficiency of perovskite oxides toward OER. For instance, Shao group investigated A-site cation deficiency in ${\mathrm{La}}_{{1{-}x}} {\mathrm{FeO}}_{{3}}$ and found that samples with appropriate A-site cation deficiency (*x* $\leqslant { 0}{\mathrm{.1}}$) maintained their perovskite oxide structure and the ${\mathrm{La}}_{0.95}{\mathrm{Fe}}{\mathrm{O}}_{3}$ with an *x* of 0.05 exhibited the highest catalytic activity originating from the slightly more surface oxygen vacancies and ${\mathrm{Fe}}^{4 + }$ ions (Fig. 8a) [109]. Likewise, benefiting from the increased charge transfer ability caused by the increased bond angle of Ni–O–Ni and an expanded bandwidth, ${\mathrm{La}}_{0.95}{\mathrm{Ni}}{\mathrm{O}}_{3}$ films with 5% La atom deficiency exhibited an enhanced OER performance, whereas ${\mathrm{La}}_{0.90}{\mathrm{Ni}}{\mathrm{O}}_{3}$ and ${\mathrm{La}}_{0.85}{\mathrm{Ni}}{\mathrm{O}}_{3}$ films with excessive La deficiency became more insulating than the pristine ${\mathrm{LaNi}}{\mathrm{O}}_{3}$ and consequently exhibited poor OER activity (Fig. 8b) [110]. Zhao et al. also examined A-site double cation deficiencies of ${\mathrm{(La}}_{{{0}{\mathrm{.6}}}} {\mathrm{Sr}}_{{{0}{\mathrm{.4}}}} {)}_{{{1}{-}x}} {\mathrm{Co}}_{{{0}{\mathrm{.8}}}} {\mathrm{Fe}}_{{{0}{\mathrm{.2}}}} {\mathrm{O}}_{{{3}{-}\delta }}$ and ${\mathrm{(Ba}}_{{{0}{\mathrm{.5}}}} {\mathrm{Sr}}_{{{0}{\mathrm{.5}}}} {)}_{{1{-}x}} {\mathrm{Co}}_{{{0}{\mathrm{.8}}}} {\mathrm{Fe}}_{{{0}{\mathrm{.2}}}} {\mathrm{O}}_{{{3}{-}\delta }}$ toward OER [111], the results displayed that double cation deficiencies could create more oxygen vacancies and higher oxidation states of B-site cations than individual A-site deficiency, as further confirmed by the investigation on A-site deficient perovskite oxide of ${\mathrm{(}{\mathrm{La}}_{0.6}{\mathrm{Sr}}_{0.4}\mathrm{)}}_{0.95}{\mathrm{MnO}}_{3}$ [112]. Except for the A-site deficiency, Zhang et al. also investigated A-site excessive perovskite oxide toward OER, but the results still supported the conclusion that A-site deficient ${\mathrm{La}}_{0.9}{\mathrm{Mn}}{\mathrm{O}}_{3}$ possessed a larger amount of oxygen vacancies, a stronger adsorption ability of oxygenated species and an upper d-band center of Mn cations than pristine ${\mathrm{LaMn}}{\mathrm{O}}_{3}$ and A-site excessive ${\mathrm{La}}_{1.1}{\mathrm{Mn}}{\mathrm{O}}_{3}$ (Fig. 8c) [113]. Moreover, manipulating the Sr deficiency over ${\mathrm{La}}_{{1/3}} {\mathrm{Sr}}_{{{1}{\mathrm{.9/3}}}} {\mathrm{Co}}_{{{0}{\mathrm{.5}}}} {\mathrm{Fe}}_{{{0}{\mathrm{.5}}}} {\mathrm{O}}_{{3{-}\delta }}$ was found to promote OER with a LOM pathway (Fig. 8d), because the introduction of Sr deficiency mainly increased the oxygen vacancies concentration, which significantly enhanced the oxygen mobility and enabled a fast replenishment of lattice oxygen (Fig. 8e) [114]. Clearly, an optimal A-site cation deficiency in perovskite oxides holds the potential to induce more oxygen vacancies formations and/or higher valence states of B-site cations, both of which are normally favorable for OER, whereas excessive cation deficiency in perovskite oxides may break the lattice structure and form impurity phase, thus degrading the OER performance.

## Surface Engineering

Tremendous research efforts have been taken concentrating on the surface engineering to directionally enhance OER performance over perovskite oxides. Despite the various underlying principles of the treatments (e.g., specific surface area, crystallinity, defects and hydrophilicity) discussed herein, the preferential surface chemistry of perovskite oxides has been demonstrated to make a difference in the heterogeneous catalysis toward OER, which normally could essentially facilitate mass transfer rates and enrich substantial amounts of highly active sites for desirable OER with higher catalytic efficiencies. From this point of view, this review summarizes, as below, the recent advances in surface engineering of perovskite oxides, mainly including size tuning, morphology control, amorphization and surface modification.

### Size Tuning

To address the limited catalytic activity over bulk-sized materials, researchers have switched their attention to synthesizing nanoscale materials. In fact, it is widely accepted that the particle size of perovskite oxides increases with ramping annealing temperature [115]. Inspired by this, Zhou et al. prepared ${\mathrm{LaCo}}{\mathrm{O}}_{3}$ by varying particle size through accurately controlling the annealing temperature and observed a beneficial tuning of ${\mathrm{e}}_{\mathrm{g}}$ filling of Co cations from 1.0 of 1 µm to 1.2 of 80 nm of ${\mathrm{LaCo}}{\mathrm{O}}_{3}$ due to the spin-state transition from low-spin to high-spin of ${\mathrm{Co}}^{3 + }$ induced by the size control [116]. Analogously, Wang et al. demonstrated that lowering the calcination temperature could dramatically reduce the particle size and enhance the covalency of M–O bonds, where a calcination temperature of 800 °C enabled ${\mathrm{SrCo}}_{{{0}{\mathrm{.5}}}} {\mathrm{Fe}}_{{{0}{\mathrm{.5}}}} {\mathrm{O}}_{{3{-}\delta }}$ to deliver a *j* of 10 mA cm^−2^ at a low *η* of 327 mV in 0.1 M KOH, superior to the benchmark ${\mathrm{Ru}}{\mathrm{O}}_{2}$ [117]. Therefore, the size effects of perovskite oxides toward OER cannot be ignored. Moreover, the size dependence on annealing temperature is further demonstrated by the ${\mathrm{La}}_{{{0}{\mathrm{.6}}}} {\mathrm{Sr}}_{{{0}{\mathrm{.4}}}} {\mathrm{CoO}}_{{3{-}\delta }}$ with different particle sizes from 60 to 450 nm annealed at different calcination temperatures. However, owing to a better crystallinity and a higher purity, reversed phenomena were detected on ${\mathrm{La}}_{{{0}{\mathrm{.6}}}} {\mathrm{Sr}}_{{{0}{\mathrm{.4}}}} {\mathrm{CoO}}_{{3{-}\delta }}$ calcined at 900 (LSC900) and 1 000 °C (LSC1000), which possessed smaller specific surface areas but exhibited better catalytic activities. Moreover, a ball milling of LSC1000 increased the specific surface area over 10 times and improved the OER performance [118]. Shao et al. verified this by reporting a significantly enhanced mass activity of ${\mathrm{SrNb}}_{{{0}{\mathrm{.1}}}} {\mathrm{Co}}_{{{0}{\mathrm{.7}}}} {\mathrm{Fe}}_{{{0}{\mathrm{.2}}}} {\mathrm{O}}_{{3{-}\delta }}$ after ball milling due to the reduced particle size and the correspondingly increased specific surface area [29]. Also, Yamada et al. ground the ${\mathrm{Ca}}{\mathrm{Cu}}_{3}{{\mathrm{Fe}}}_{4}{{\mathrm{O}}}_{12}$ through ball milling and obtained a largely increased specific surface area from 0.38 to 10.30 ${\mathrm{m}}^{{2}} {\mathrm{g}}^{{{-}1}}$, which improved the OER catalytic activity, but a long-time ball milling reversely lowered the OER catalytic activity, possibly due to the degradation of crystallinity [119]. Accordingly, Khaerudini et al. found that a longer ball milling time and a higher ratio of balls to powders could cause an increase in the content of impurity of ${\mathrm{Bi}}_{{{0}{\mathrm{.7}}}} {\mathrm{Sr}}_{{{1}{\mathrm{.3}}}} {\mathrm{Co}}_{{{0}{\mathrm{.5}}}} {\mathrm{Fe}}_{{{1}{\mathrm{.5}}}} {\mathrm{O}}_{{6{-}\delta }}$, which in turn deteriorated oxygen desorption [120]. Nevertheless, the particle size of the as-prepared materials strongly depends on the synthetic approach. For example, La doping in BSCF, i.e., ${\mathrm{La}}_{{{0}{\mathrm{.7}}}} {\mathrm{(Ba}}_{{{0}{\mathrm{.5}}}} {\mathrm{Sr}}_{{{0}{\mathrm{.5}}}} {)}_{{{0}{\mathrm{.3}}}} {\mathrm{Co}}_{{{0}{\mathrm{.8}}}} {\mathrm{Fe}}_{{{0}{\mathrm{.2}}}} {\mathrm{O}}_{{3{-}\delta }}$, could remarkably decrease the particle size from 100 nm at 900 °C to 50 nm at 700 °C [121], while a flame spraying could synthesize perovskite oxide with a smaller particle size and a better OER activity as compared to the conventional sol-gel method [62].

### Morphology Control

**Fig. 9:**
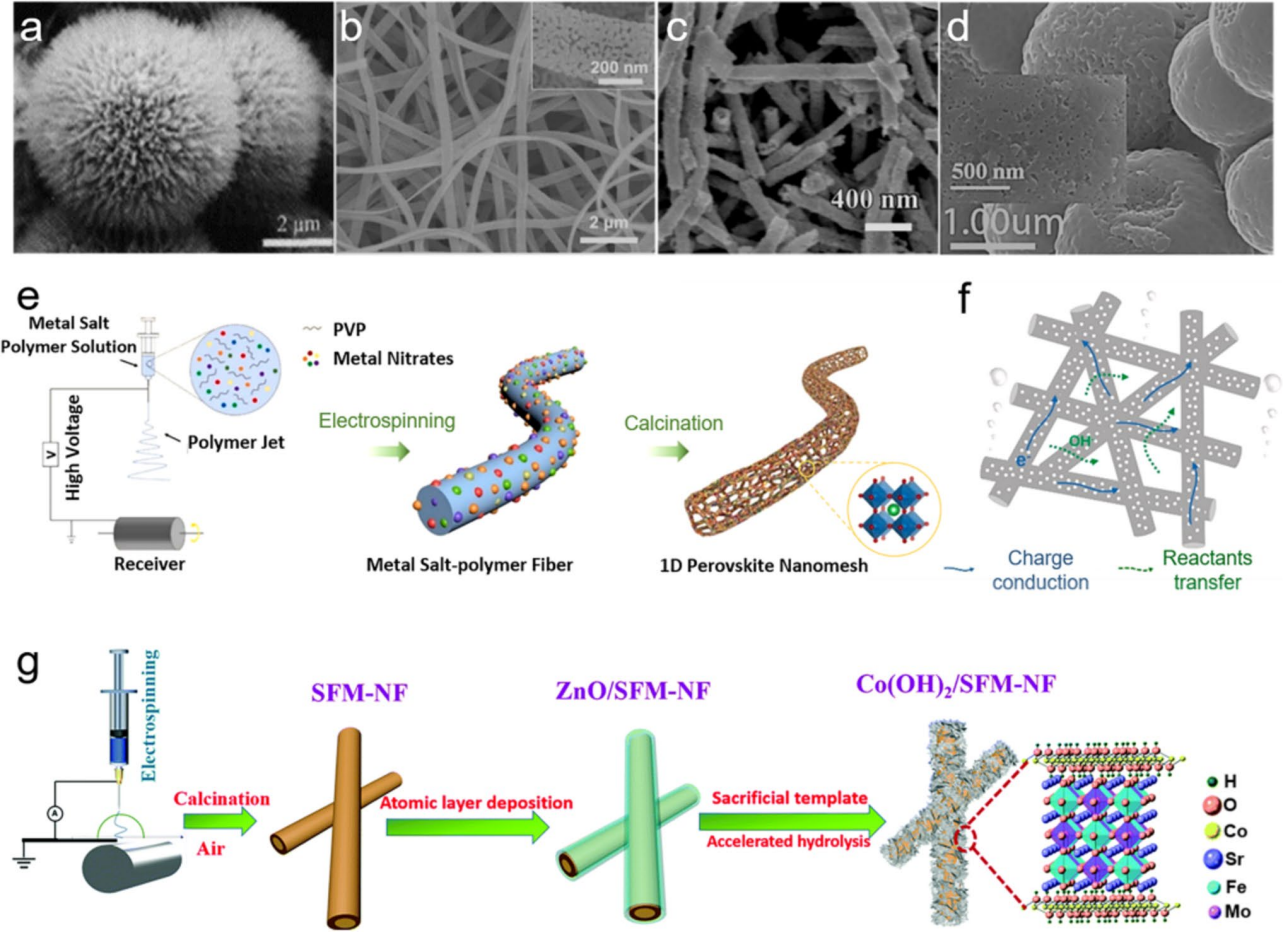
Scanning electron microscope (SEM) images of (**a**) urchin-like ${\mathrm{La}}_{0.8}{\mathrm{Sr}}_{0.2}{\mathrm{Mn}}{\mathrm{O}}_{3}$, **b** 1D ${\mathrm{La}}_{0.5}{\mathrm{Ba}}_{0.5}{\mathrm{Co}}_{0.8}{\mathrm{Ni}}_{0.2}{\mathrm{O}}_{{3}-\delta}$ nanomeshes, **c** hierarchical mesoporous/macroporous ${\mathrm{La}}_{0.5}{\mathrm{Sr}}_{0.5}{\mathrm{Co}}{\mathrm{O}}_{{3}-\delta}$ nanotubes and **d** porous ${\mathrm{GdFe}}{\mathrm{O}}_{3}$ nanospheres. Adapted with permission from Ref. [51, 124, 130, 132]. Copyright © 2013, Elsevier Ltd.; Copyright © 2021, Elsevier Ltd.; Copyright © 2015, American Chemical Society; Copyright © 2021, American Chemical Society. **e** The synthesis procedures of 1D ${\mathrm{La}}_{0.5}{\mathrm{Ba}}_{0.5}{\mathrm{Co}}_{0.8}{\mathrm{Ni}}_{0.2}{\mathrm{O}}_{{3}-\delta}$ nanomeshes. Schematic diagrams of (**f**) favorable charge conduction and reactants transfer during OER. **g** The synthetic process of the ${\mathrm{Co}}{\mathrm{(OH)}}_{2}$/${\mathrm{Sr}}_{2}{{\mathrm{Fe}}}_{1.5}{\mathrm{Mo}}_{0.5}{\mathrm{O}}_{\mathrm{5} + \delta}$. Adapted with permission from Ref. [133]. Copyright © 2020, The Royal Society of Chemistry

Constructing specific morphologies of perovskite oxides to explore the corresponding electrochemical performance and establishing the associated nanostructures—activities relationships are of prime importance to provide direction for future research. Generally, perovskite oxide nanostructures with favored porosity and large specific area could be obtained by finely controlling the synthetic conditions, which is favorable to the diffusion of gas reactants/products during the OER process and offer an increased number of reactive sites. Currently, various specific nanostructures, e.g., nanofibers [79, 122, 123], nanotubes [124–126], nanospheres [127], and nanowires [128], have been reported to promote OER with varying degrees of success. Representatively, ${\mathrm{La}}_{{{0}{\mathrm{.5}}}} {\mathrm{(Ba}}_{{{0}{\mathrm{.4}}}} {\mathrm{Sr}}_{{{0}{\mathrm{.4}}}} {\mathrm{Ca}}_{{{0}{\mathrm{.2}}}} {)}_{{{0}{\mathrm{.5}}}} {\mathrm{Co}}_{{{0}{\mathrm{.8}}}} {\mathrm{Fe}}_{{{0}{\mathrm{.2}}}} {\mathrm{O}}_{{3{-}\delta }}$ nanorods with rich oxygen vacancies on the surface achieved an optimal ${\mathrm{e}}_{\mathrm{g}}$ occupation and excellent OER/hydrogen evolution reaction (HER) catalytic activities [129]. Similarly, ${\mathrm{La}}{\mathrm{Fe}}_{0.2}{\mathrm{Ni}}_{0.8}{\mathrm{O}}_{3}$ nanorods with a modified electronic structure and a largely enhanced specific surface area of 58.1 ${\mathrm{m}}^{{2}} {\mathrm{g}}^{{{-}1}}$ also delivered a desirable OER performance [52]. More exquisitely, an urchin-like ${\mathrm{La}}_{0.8}{\mathrm{Sr}}_{0.2}{\mathrm{Mn}}{\mathrm{O}}_{3}$ (Fig. 9a) with an ultrahigh specific surface area of 48 ${\mathrm{m}}^{{2}}\, {\mathrm{g}}^{{{-}1}}$ was synthesized through a co-precipitation method, which exhibited considerably superior catalytic activities for both OER and ORR (oxygen reduction reaction) in 0.1 mol L^–1^ KOH solution [51].

It is worth noting that specific nanostructures of perovskite oxides not only can increase the specific surface area, but also can stimulate particular effects in certain situations toward OER. For instance, Xiong et al. demonstrated that, besides the larger specific surface area, 1D ${\mathrm{La}}_{{{0}{\mathrm{.8}}}} {\mathrm{Sr}}_{{{0}{\mathrm{.2}}}} {\mathrm{Co}}_{{{0}{\mathrm{.2}}}} {\mathrm{Fe}}_{{{0}{\mathrm{.8}}}} {\mathrm{O}}_{{3{-}\delta }}$ nanofibers simplified the charge transport pathways and speeded up the transport rates of electrons and ions as compared to the counterparts of nanorods and NPs [123]. Meanwhile, Huang et al. fabricated ${\mathrm{La}}_{{{0}{\mathrm{.5}}}} {\mathrm{Ba}}_{{{0}{\mathrm{.5}}}} {\mathrm{Co}}_{{{0}{\mathrm{.8}}}} {\mathrm{Ni}}_{{{0}{\mathrm{.2}}}} {\mathrm{O}}_{{3{-}\delta }}$ nanomeshes by annealing metal salt-polymer fibers (Fig. 9e), where the unique cross-linked and highly porous nanostructure could expose more active sites (Fig. 9b) and enable an optimized electronic structure and an enhanced electron transfer ability (Fig. 9f) [130]. Besides, specific nanostructure could also accelerate mass transfer of electrolyte, as confirmed by the porous Co-doped ${\mathrm{Pr}}_{{{0}{\mathrm{.5}}}} {\mathrm{Ba}}_{{{0}{\mathrm{.5}}}} {\mathrm{MnO}}_{{3{-}\delta }}$ nanofibers, where an easier accessibility of electrolyte to the electrochemically active sites, together with the well-distributed oxygen diffusion channels, was observed [131]. The similar phenomenon was also observed on hierarchical mesoporous or microporous ${\mathrm{La}}_{{{0}{\mathrm{.5}}}} {\mathrm{Sr}}_{{{0}{\mathrm{.5}}}} {\mathrm{CoO}}_{{3{-}\delta }}$ nanotubes (Fig. 9c) where a superior OER performance was achieved by virtue of a comparably improved ion transfer rate, a highly facilitated electron transfer rate and a large specific surface area [124]. Aside from constructing specific nanostructures of perovskite oxides, it is equally important to decorate intrinsically more active materials (e.g., ${\mathrm{Pt}}{\mathrm{O}}_{{x}}$ + Ni/NiO NPs) on the nanostructured perovskite oxides (e.g., porous sphere-like ${\mathrm{GdFe}}{\mathrm{O}}_{3}$) to balance their strengths and weakness and consequently reach a win–win situation to enable a fast surface charge transfer and an efficient oxygen electrocatalysis at the interfaces of material–material and material–electrolyte (Fig. 9d) [132]. For example, Zhao et al. integrated the electrospinning and the atomic layer deposition techniques to decorate amorphous ${\mathrm{Co}}{\mathrm{(OH)}}_{2}$ nanoflakes on the surface of ${\mathrm{Sr}}_{{2}} {\mathrm{Fe}}_{{{1}{\mathrm{.5}}}} {\mathrm{Mo}}_{{{0}{\mathrm{.5}}}} {\mathrm{O}}_{5 + \delta }$ nanofibers for the construction of a crystallized core-amorphous shell nanostructure (Fig. 9g). Benefitting from the unique nanostructure and the synergistic effects between ${\mathrm{Co}}{\mathrm{(OH)}}_{2}$ and ${\mathrm{Sr}}_{{2}} {\mathrm{Fe}}_{{{1}{\mathrm{.5}}}} {\mathrm{Mo}}_{{{0}{\mathrm{.5}}}} {\mathrm{O}}_{5 + \delta }$, the core–shell nanostructured composite possessed a large electrochemical surface area with abundant oxygen vacancies, which delivered a fast electron transfer ability and superior bifunctional OER and HER performances under the harsh alkaline media [133]. Simply put, constructing specific nanostructures of perovskite oxides indeed provides a smart design strategy to efficiently drive OER, which could not only greatly enlarge specific surface areas to maximize the exposure of active sites and guarantee the easy accessibility of reactants to the active sites, but also optimize the charge and reactants transfer pathways.

### Amorphization

**Fig. 10:**
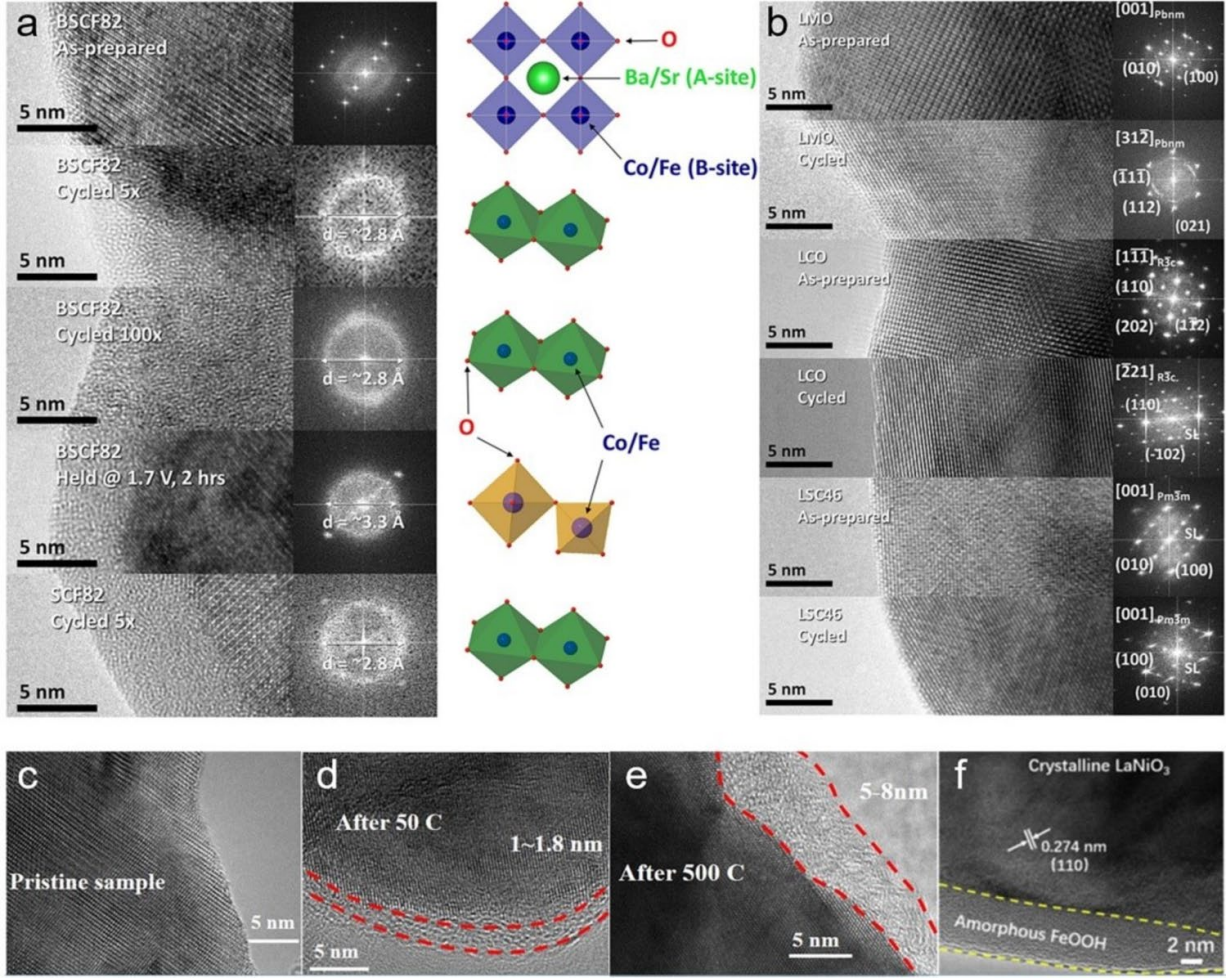
High resolution TEM (HRTEM) images and corresponding fast Fourier transforms of (**a**) as-prepared BSCF82, the ones after 5 and 100 CV cycles as well as the one potentiostatically tested at 1.7 V for 2 h and ${\mathrm{Sr}}{\mathrm{Co}}_{0.8}{\mathrm{Fe}}_{0.2}{\mathrm{O}}_{{3}-\delta}$ (SCF82) after 5 CV cycles, and **b** pristine and cycled perovskite oxides of LMO, LCO and LSC46. Adapted with permission from Ref. [135]. Copyright © 2012, American Chemical Society. HRTEM images of (**c**) pristine ${\mathrm{La}}_{2}{\mathrm{MnNi}}{\mathrm{O}}_{6}$, **d** the ones after 50 and **e** 500 CV cycles. Adapted with permission from Ref. [95]. Copyright © 2018, American Chemical Society. And **f** as-prepared ${\mathrm{LaNi}}{\mathrm{O}}_{3}\mathrm{@FeOOH}$. Adapted with permission from Ref. [142]. Copyright © 2020, Elsevier Ltd.

In early 1980s, Kobussen and coworkers first reported that amorphous hydrated cobalt oxides were formed on the surface of ${\mathrm{La}}_{0.5}{\mathrm{Ba}}_{0.5}{\mathrm{Co}}{\mathrm{O}}_{3}$ after a long-term anodic polarization in alkaline solution, which has sparked extensive and worldwide research attention in the past few decades due to its non-negligible impact on electrocatalysis [134]. For the state-of-the-art BSCF upon initiating cyclic voltammetry (CV) or potentiostatic tests for OER, a rapid amorphization occurred on the surface, accompanied by a leaching of A-site cations, which in turn dramatically increased the OER catalytic activity of BSCF, whereas the similarly prepared ${\mathrm{LaMn}}{\mathrm{O}}_{3}$ (LMO), ${\mathrm{LaCo}}{\mathrm{O}}_{3}$ (LCO) and ${\mathrm{La}}_{{{0}{\mathrm{.4}}}} {\mathrm{Sr}}_{{{0}{\mathrm{.6}}}} {\mathrm{CoO}}_{{3{-}\delta }}$ (LSC46) still maintained their crystallized surfaces (Fig. 10a, b) [135]. In parallel, the dynamic surface self-reconstruction of BSCF, accompanied with LOER and the dissolution of metal cations, was captured using time-resolved X-ray absorption spectroscopy (XAS) [136]. Indeed, the amorphization and the self-reconstruction share the similar phenomenon on perovskite oxide surface during OER, i.e., dynamic structural transformation, both of which involve the dissolution of A-site cations and the formation of an amorphous layer due to the redeposition of B-site cations. Subsequently, Xie et al. reported that the amorphous layer composed of active species of Mn/Ni oxide/hydroxide was gradually formed on the surface of ${\mathrm{La}}_{2}{\mathrm{MnNi}}{\mathrm{O}}_{6}$ during CV tests, and the thickness and the displayer OER activity increased with cycle number (Fig. 10c–e) [95]. It is worth noting that perovskite oxides are much more prone to transform into amorphous species in acidic solution because the cations are more easily leached. Limited by this, only Ir- and Ru-based perovskite oxides could be available for OER in acidic media [137, 138]. Xu et al. reported that the Sr and Co in ${\mathrm{SrCo}}_{{{0}{\mathrm{.9}}}} {\mathrm{Ir}}_{{{0}{\mathrm{.1}}}} {\mathrm{O}}_{{3{-}\delta }}$ would dissolve in 0.1 mol L^−1^ ${\mathrm{HClO}}_{{4}}$ during the OER cycles and transform into amorphous ${\mathrm{IrO}}_{x} {\mathrm{H}}_{y}$ species where the newly formed undercoordinated ${\mathrm{IrO}}_{x}$ octahedrons in the amorphous ${\mathrm{IrO}}_{{x}}{{\mathrm{H}}}_{{y}}$ species are responsible for the high OER catalytic activity [139]. This is also confirmed by Grimaud group in that ${\mathrm{Sr}}_{2}{\mathrm{MIr}}{\mathrm{O}}_{6}$ (M = Fe, Co) and ${\mathrm{Sr}}_{2}{{\mathrm{Fe}}}_{0.5}{\mathrm{Ir}}_{0.5}{\mathrm{O}}_{4}$ exhibited similar OER activities after several cycles regardless of their crystal structures and chemical composition, since the OER activities of Ir-based perovskite oxides are ultimately dominated by the amorphous phases of ${\mathrm{IrO}}_{x} \cdot m{\mathrm{H}}_{{2}} {\mathrm{O}}$ species on their surface [140]. Therefore, the as-prepared perovskite oxides may not represent the real active components under the OER conditions, which are called the pre-catalysts. Despite the instantaneous occurrence of structural change to form an amorphous layer on the surfaces of some certain perovskite oxides under the harsh OER conditions, the newly formed structures could maintain superb stabilities toward OER and display excellent OER activity without exception.

Inspired by this, directionally modulating the surface amorphization with different approaches to construct amorphous active layers on the surface of perovskite oxides has been proposed to improve OER catalytic activity and has been well recognized by the scientific community. For example, Zhang et al. reported that doping Ce in ${\mathrm{La}}_{0.9}{\mathrm{Ce}}_{0.1}{\mathrm{Ni}}{\mathrm{O}}_{3}$ could not only upshift the O 2p band center and increase the oxygen vacancies content, but also greatly facilitate the in situ formation of active NiOOH species on the surface and lower the reconstruction potential, all of which consequently enabled an enhanced OER activity. Moreover, benefitting from the limited electrolyte penetration, the perovskite oxides bulk could remain unchanged to ensure a long-term OER stability [53]. In addition, using a post-processing approach, an amorphous layer of $\mathrm{Co/FeO}{\mathrm{(OH)}}_{{x}}$ on the ${\mathrm{La}}_{0.8}{\mathrm{Sr}}_{0.2}{\mathrm{Co}}_{0.8}{\mathrm{Fe}}_{0.2}{\mathrm{O}}_{{3}-\delta}$ surface was ex situ fabricated in an aqueous ${\mathrm{NaB}}{\mathrm{H}}_{4}$ solution, but it only afforded an *η* of 248 mV to deliver 10 mA cm^−2^ and a Tafel slope of 51 $\text{mV }{\mathrm{dec}}^{-{1}}$ [141]. More recently, an amorphous layer of FeOOH on ${\mathrm{LaNi}}{\mathrm{O}}_{3}$ surface was synthesized (Fig. 10f) and achieved a *j* of 10 mA cm^−2^ at a lowered *η* of 264 mV, resulting from the increased active sites, the favorable adsorption energy of the oxygenated species and the fast charge transfer rate [142]. With the mature techniques, amorphous BSCF film was directly fabricated by photochemical deposition for the first time, and it exhibited a remarkably higher OER performance than the state-of-the-art crystalline BSCF, mainly derived from the high concentration of surface coordinately unsaturated metal sites (i.e., Co and trace amounts of Fe cations) which were considered as the active sites for boosting OER [143]. A further study using DFT calculations again confirmed that the appropriate oxidation state of Co cations favored the adsorption and desorption of intermediates [144]. In recent years, amorphous materials for OER have been brought to the forefront and inspired extensive studies, but the structural discrepancy between perovskite oxide pre-catalyst and the eventual reconstructed active species poses difficulties in establishing precise structure–activity relationships and designing targeted perovskite oxide catalysts. To go further into this topic and solve this issue, it is imperative to develop in situ/operando techniques to monitor the molecular-level atomic structure and electronic structure of the outermost surface of perovskite oxides and deeply understand the instantaneous steps in the evolving process of amorphous layer formation, and its subsequent electrochemical behaviors and the underlying mechanisms toward OER [145–147].

### Surface Modification

**Fig. 11:**
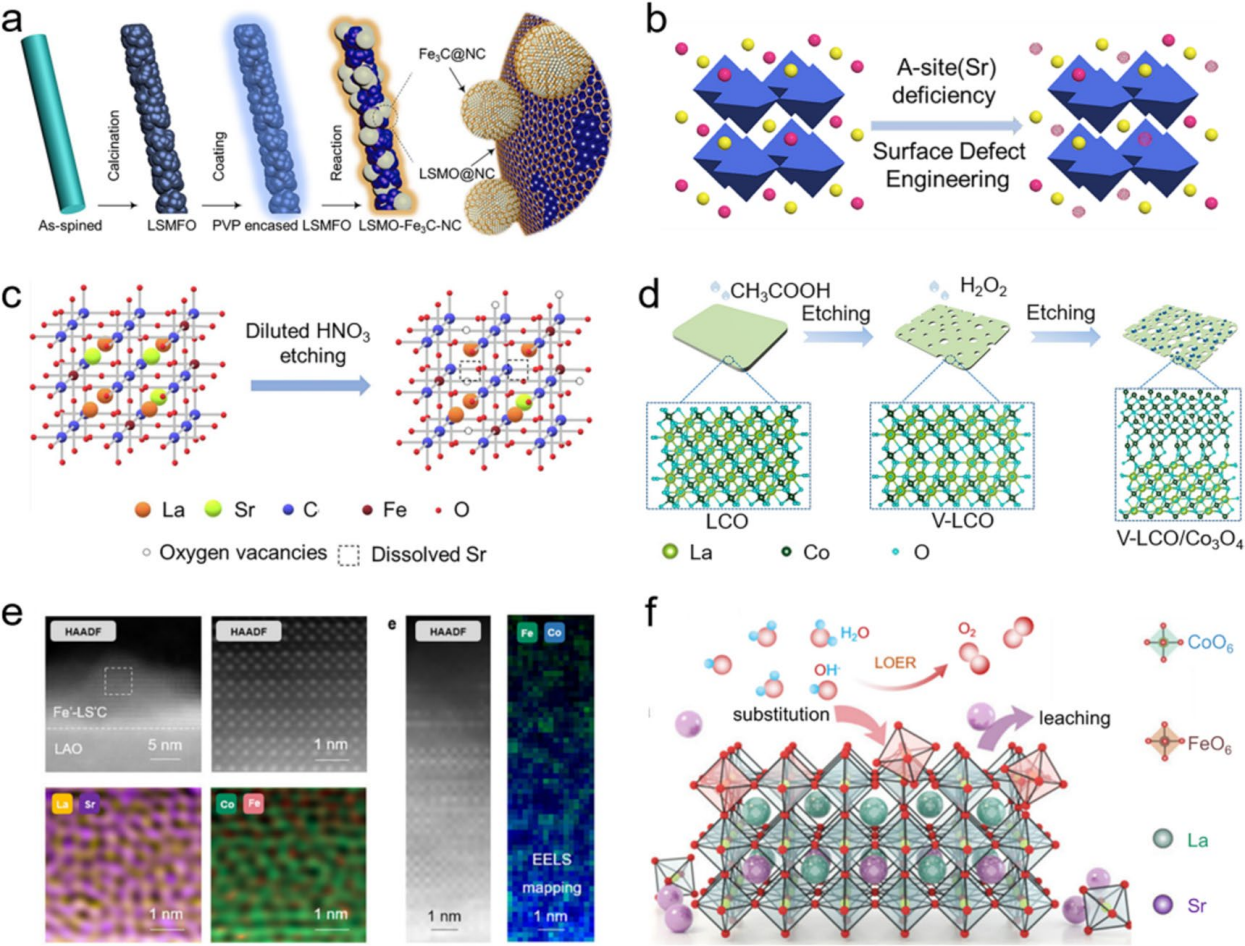
**a** The synthesis procedures of $\mathrm{LSMO} - {\mathrm{Fe}}_{3}{\mathrm{C}}-{\mathrm{NC}}$. Adapted with permission from Ref. [150]. Copyright © 2018, Elsevier Ltd. **b** The surface defect engineering by selective A-site acid etching. Adapted with permission from Ref. [152]. Copyright © 2021, American Chemical Society. **c** The diluted ${\mathrm{HN}}{\mathrm{O}}_{3}$ etching of ${\mathrm{La}}_{0.6}{\mathrm{Sr}}_{0.4}{\mathrm{Co}}_{0.8}{\mathrm{Fe}}_{0.2}{\mathrm{O}}_{{3}-\delta}$. Adapted with permission from Ref. [153]. Copyright © 2020, Elsevier Ltd. **d** The formation for $\mathrm{V-LCO/}{\mathrm{Co}}_{3}{{\mathrm{O}}}_{4}$ heterostructure by double-cation gradient etching. Adapted with permission from Ref. [154]. Copyright © 2021, American Chemical Society. **e** High-angle annular dark-field images, energy dispersive x-ray (EDX) spectroscopy mapping, and electron energy loss spectroscopy (EELS) mapping of surface Fe doped ${\mathrm{La}}_{{{0}{\mathrm{.5}}}} {\mathrm{Sr}}_{{{0}{\mathrm{.5}}{-}\delta }} {\mathrm{CoO}}_{{3}}$. **f** Schematic illustration of OER via LOM pathway. Adapted with permission from Ref. [156]. Copyright © 2022, The Royal Society of Chemistry

It is well known that the complicated proton-coupled electron transfer steps during OER occur at triple phase interfaces; thus the physiochemical properties of the perovskite oxide surface itself has an important impact on OER catalytic behaviors. In particular, a good surface hydrophilicity is an important prerequisite, since it ensures the intimate contact of heterogeneous species at the triple phase interfaces. Zhao and coworkers grew porous ${\mathrm{LaCo}}{\mathrm{O}}_{3}$ nanosheets vertically on nickel foam, which could reduce the bubble adhesive force and accelerate the dissipation of gas bubbles caused by the increase in surface hydrophilicity upon proceeding OER [148]. Equivalently, Co compounds [i.e., ${\mathrm{CoO(OH)}}/{\mathrm{Co}}_{{2}} {\mathrm{(CO}}_{{3}} {\mathrm{)(OH)}}_{{2}} \cdot {0}{\mathrm{.22H}}_{{2}} {\mathrm{O}}$ or ${\mathrm{Co}}_{{3}} {\mathrm{O}}_{{4}}$] were employed to modify surface hydrophilicity of ${\mathrm{(}{\mathrm{La}}_{0.8}{\mathrm{Sr}}_{0.2}\mathrm{)}}_{0.95}{\mathrm{Mn}}{\mathrm{O}}_{3}$ by a hydrothermal method, and it was found that the high concentration of adsorbed–OH on surface facilitated the formation of ${\mathrm{HO}}{\mathrm{O}}^{-}$ and consequently enhanced the OER performance [149]. Motivated by this, Luo group further designed an integrated composite with three components (i.e., ${\mathrm{La}}_{0.5}{\mathrm{Sr}}_{0.5}{\mathrm{Mn}}{\mathrm{O}}_{{3}-\delta}$ nanorods, ${\mathrm{Fe}}_{3}{\mathrm{C}}$ NPs and N-doped carbon) for OER (Fig. 11a), where the N-doped carbon materials became highly hydrophilic after heating with ${\mathrm{La}}_{{{\mathrm{0}}{\mathrm{.5}}}} {\mathrm{Sr}}_{{{\mathrm{0}}{\mathrm{.5}}}} {\mathrm{MnO}}_{{3{-}\delta }}$, consequently accelerating the adsorptions of ${\mathrm{H}}_{2}{\mathrm{O}}$ and ${\mathrm{O}}{\mathrm{H}}^{-}$ on the composite [150].

As of this writing, various strategies have been adopted to modify the surfaces of perovskite oxides for the enhancement of OER performance. Typically, Wu et al. developed a controllable lithium reduction strategy to build a defective surface of ${\mathrm{La}}_{0.6}{\mathrm{Sr}}_{0.4}{\mathrm{Co}}_{0.2}{\mathrm{Fe}}_{0.8}{\mathrm{O}}_{{3}-\delta}$ capable of lowering *η* of 100 mV to afford a *j* of 10 mA cm^−2^ induced by the improved oxygen exchange kinetics, electrical conductivity and structural durability [151]. Different from the A-site deficiency caused by the nonstoichiometric A/B molar ratio, the post-processing acid etching strategies have also been employed to enhance OER performances benefiting from the increased exposure of active sites, decreased coordination of B-site cations and tailored local electronic structure induced by the introduction of A-site deficiency on the surfaces of perovskite oxides. For instance, Shao et al. selectively corroded A-site Sr by ${\mathrm{HN}}{\mathrm{O}}_{3}$ etching to regulate surface defect of ${\mathrm{La}}_{0.6}{\mathrm{Sr}}_{0.4}{\mathrm{Co}}_{0.2}{\mathrm{Fe}}_{0.8}{\mathrm{O}}_{{3}-\delta}$ (Fig. 11b), which not only increased the exposure of B-site cations and optimized its electronic structure, but also decreased the coordination of B-site metals [152]. Similarly, selectively dissolving A-site cation of ${\mathrm{La}}_{0.6}{\mathrm{Sr}}_{0.4}{\mathrm{Co}}_{0.8}{\mathrm{Fe}}_{0.2}{\mathrm{O}}_{{3}-\delta}$ would also enrich highly oxidative oxygen species on the surface and increase the specific surface area (Fig. 11c) [153]. However, a long-term acid etching induced a large deviation of ${\mathrm{e}}_{\mathrm{g}}$ occupancy value of 1.2, resulting in a drop in OER catalytic activity. Shortly after, Gao et al. utilized a double-cation gradient etching process to modify the surface electronic structure of ${\mathrm{LaCo}}{\mathrm{O}}_{3}$ and obtained a perovskite oxide/spinel oxide heterostructure (${\mathrm{LaCo}}{\mathrm{O}}_{3}\mathrm{/}{\mathrm{Co}}_{3}{{\mathrm{O}}}_{4}$) with a gradient dissolution of A-site cations (Fig. 11d) [154]. The DFT calculations revealed that the d-band center of heterostructure was located at an appropriate position and as a result, possessed an optimal adsorption/desorption ability for oxygenated species. In parallel, Zhang et al. fabricated a ${\mathrm{NiO}}-{\mathrm{LaNi}}{\mathrm{O}}_{3}$ heterostructure via selective dissolution of A-site Sr cation in ${\mathrm{La}}_{0.95}{\mathrm{Sr}}_{0.05}{\mathrm{Ni}}{\mathrm{O}}_{{3{-}}\delta}$ using a facile acid etching method, and achieved an excellent OER activity in 1.0 mol L^−1^ KOH due to the high oxygen vacancy content and the large specific surface area. Moreover, the in situ exsolved NiO would transform into highly active NiOOH species after long-term electrochemical activation, which further improved the OER performance [155]. With one step further, by etching perovskite oxide in the acidic media containing ${\mathrm{Fe}}^{3+}$, Li et al. prepared ${\mathrm{La}}_{0.5}{\mathrm{Sr}}_{{0.5{-}}\delta}{\mathrm{Co}}{\mathrm{O}}_{3}$ with surface Fe sites and Sr deficiencies (Fig. 11e), and demonstrated that Sr deficiencies could accelerate the LOER by tailoring the O p band center, while surface Fe sites are the pivotal catalytic centers for LOER (Fig. 11f) [156]. Clearly, these results demonstrate the importance of surface physiochemical and structural properties (e.g., hydrophilicity, specific surface area and coordination environment) of perovskite oxides in heterogeneous catalysis and moreover provide the constructive insights to optimize the OER performances through intellectual surface modification, like surface coating, lithium reduction and etching.

## Structure Mutations

To meet the demands for specific applications, a variety of structure control strategies have been adopted to mutate perovskite oxides with desirable properties. In this part, we will discuss, in details, the effects of different structure mutations over perovskite oxides on intrinsically structural and physiochemical properties [e.g., electronic structure, oxygen vacancy content, M–O bonding configuration and electrical conductivity], together with the associated OER performances from the perspectives of phase transition, crystal orientation, lattice strain. Despite the different design principles, the various structure mutations share the common goal of designing high-performance perovskite oxides for OER.

### Phase Transition

**Fig. 12:**
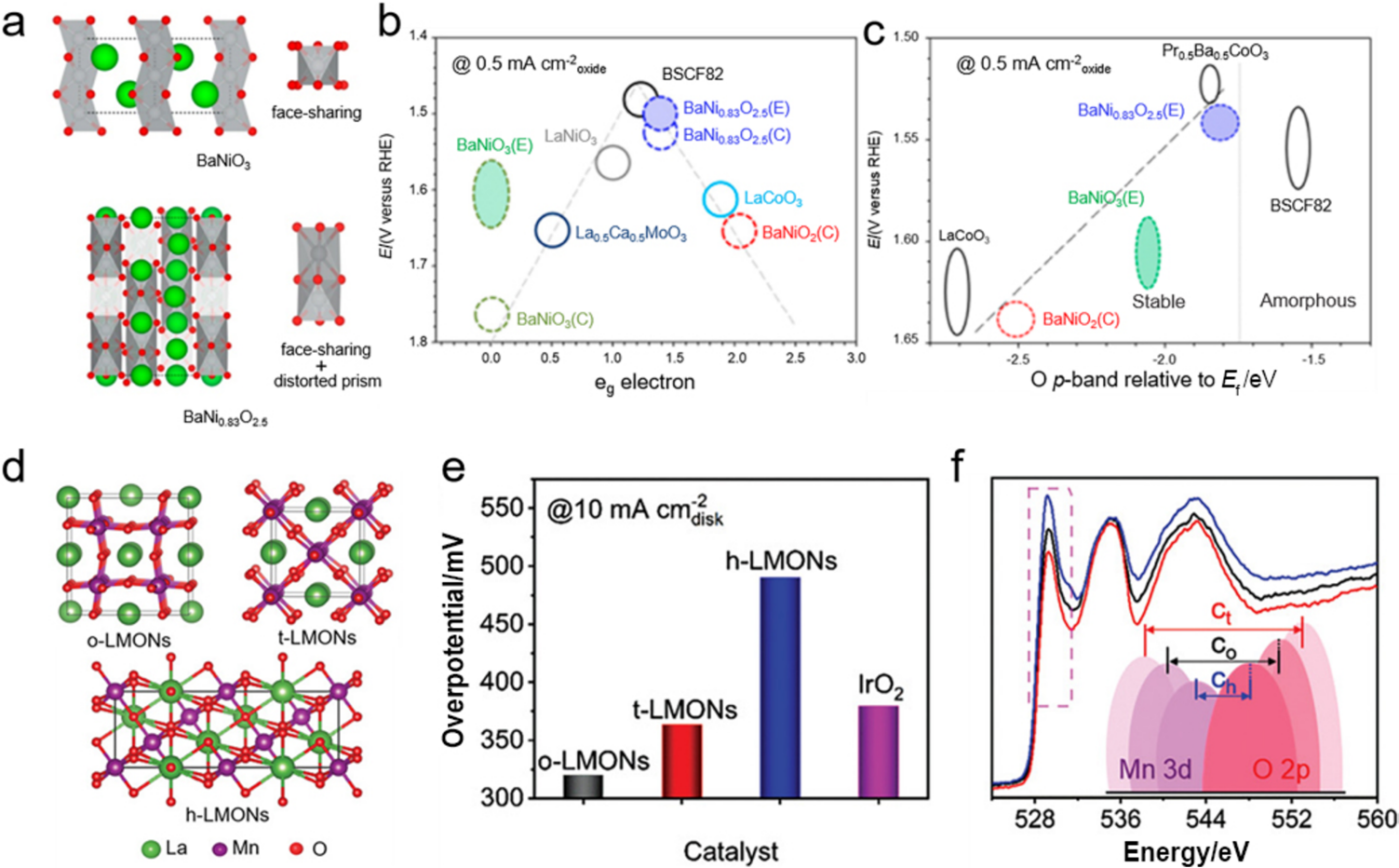
**a** Crystal structures of ${\mathrm{BaNi}}{\mathrm{O}}_{3}$ and ${\mathrm{Ba}}{\mathrm{Ni}}_{\mathrm{0.8}{3}}{\mathrm{O}}_{2.5}$. **b** The relations between potentials at ${0}{\text{.5 mA cm}}^{{{-}2}}_{{{\mathrm{oxide}}}}$ and **c** e_g_ fillings of B-site cations, and **c** O p−bands relative to *E*_*f*_ (eV) in perovskite oxides. Adapted with permission from Ref. [157]. Copyright © 2016, American Chemical Society. **d** Crystal structures, **e** *η* at 10 mA cm^−2^ and **f** O K-edge XAS spectra along with estimated Mn−O covalency of o-, t- and h-LMONs. Adapted with permission from Ref. [161]. Copyright © 2021, Wiley–VCH

It is commonly known that a good stability of perovskite oxides in alkaline solution is essential to promote steady OER. However, recent literature has demonstrated that the phase transition of perovskite oxides induced by a structure optimization would increase OER catalytic activity. For example, Han et al. reported that hexagonal ${\mathrm{BaNi}}{\mathrm{O}}_{3}$ experienced a structural transformation to ${\mathrm{Ba}}{\mathrm{Ni}}_{0.83}{\mathrm{O}}_{2.5}$ during OER (Fig. 12a**)**, where the newly formed ${\mathrm{Ba}}{\mathrm{Ni}}_{0.83}{\mathrm{O}}_{2.5}$ was located at a more proper O p-band center position and delivered an optimal ${\mathrm{e}}_{\mathrm{g}}$ occupancy (Fig. 12b, c) [157]. Such observation clearly indicated that phase transition can significantly increase the intrinsic OER catalytic activity of perovskite oxides. Motivated by this, Shao et al. doped trace amounts of Si in ${\mathrm{SrFe}}{\mathrm{O}}_{{3}-\delta}$ (i.e., ${\mathrm{Sr}}{\mathrm{Fe}}_{0.9}{\mathrm{Si}}_{0.1}{\mathrm{O}}_{{3}-\delta}$) to seduce a phase transition from tetragonal to cubic symmetry. Benefiting from the optimized Fe valence, the rich oxygen vacancies and the fast charge transfer, the cubic ${\mathrm{Sr}}{\mathrm{Fe}}_{0.9}{\mathrm{Si}}_{0.1}{\mathrm{O}}_{{3{-}}\delta}$ showed an excellent OER catalytic activity, threefold higher than that of the tetragonal ${\mathrm{SrFe}}{\mathrm{O}}_{{3}-\delta}$ [158]. Likewise, Lu et al. enabled a phase transition from hexagonal ${\mathrm{Sr}}_{2}{{\mathrm{Co}}}_{2}{{\mathrm{O}}}_{5}$ to cubic ${\mathrm{SrCo}}{\mathrm{O}}_{\mathrm{2.85}-\delta}{\mathrm{F}}_{0.15}$ through anion F doping, which significantly increased the electronic conductivity and the number of ${{\mathrm{O}}_{2}}^{{2}-}\mathrm{/}{\mathrm{O}}^{-}$ species on the surface [159]. To better understand the importance of phase transition, Tang et al. synthesized hexagonal BSCF, which delivered a better OER performance than the state-of-art cubic BSCF since the hexagonal BSCF displayed a faster charge transfer rate, a larger specific surface area and rich oxygen vacancies [160]. Furthermore, thin perovskite oxides of ${\mathrm{LaMn}}{\mathrm{O}}_{3}$ with orthorhombic, tetragonal, and hexagonal structures were synthesized by a salt-templated strategy, among which the orthorhombic ${\mathrm{LaMn}}{\mathrm{O}}_{3}$ possessed the optimal ${\mathrm{e}}_{\mathrm{g}}$ filling, the most oxygen vacancies and the strongest Mn–O covalency and hybridization (Fig. 12d–f); all of which enable a suitable surface binding energy and the highest OER performance [161]. More recently, Liu et al. reported that different phases (hexagonal, tetragonal and orthorhombic) could be obtained by replacing 5% Co of ${\mathrm{Sr}}{\mathrm{CoO}}_{3}$ by foreign elements. The electrochemical results show that the orthorhombic phase with an optimized Co–O bond possesses a better lattice oxygen diffusion and redox ability [162]. In brief, the above studies have revealed how phase transition affects OER performance of perovskite oxides, and provided us with a new vision to design new types of perovskite oxides via phase transformation.

### Crystal Orientation

**Fig. 13:**
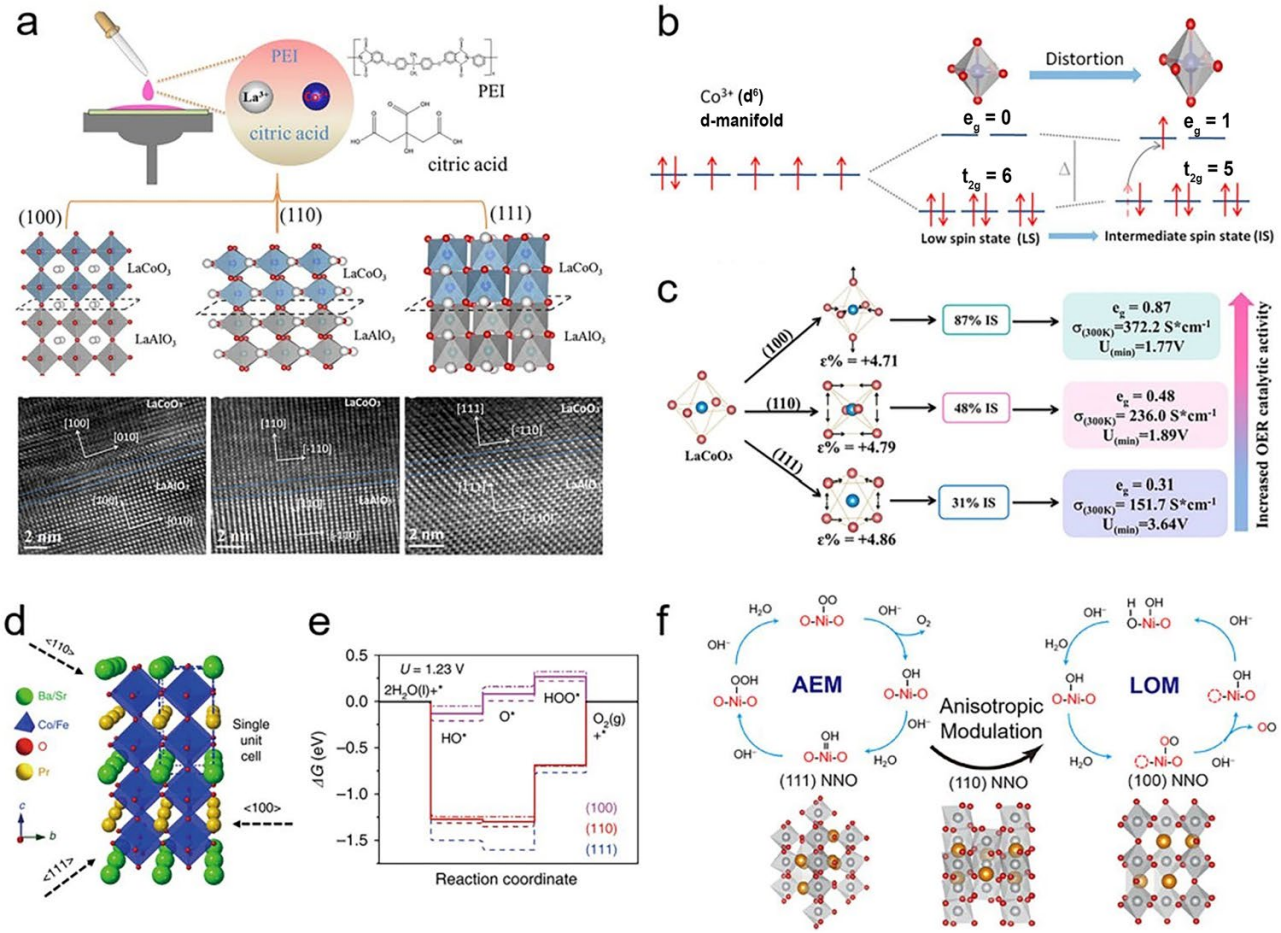
**a** Schematic illustrations and TEM images of LCO (100), LCO (110) and LCO (111) films on LAO substrates. **b** The transition of electrons from $\mathrm{t}_{2\mathrm{g}}$ to $\mathrm{e}_{\mathrm{g}}$ orbital and the evolution of electron spin state. **c** The relation between OER activity and spin configuration of variously oriented LCO films. Adapted with permission from Ref. [166]. Copyright © 2017, Cell Press. **d** Schematic drawing of the crystal structure, **e** ΔG for OER on (100), (110), (111)-oriented films of ${\mathrm{PrBa}}_{{{0}{\mathrm{.5}}}} {\mathrm{Sr}}_{{{0}{\mathrm{.5}}}} {\mathrm{Co}}_{{{1}{\mathrm{.5}}}} {\mathrm{Fe}}_{{{0}{\mathrm{.5}}}} {\mathrm{O}}_{5 + \delta }$. Adapted with permission from Ref. [168]. Copyright © 2020, Springer Nature. **f** The alternation of OER pathways on different oriented NNO films. Adapted with permission from Ref. [169]. Copyright © 2020, American Chemical Society

In agreement with the anisotropy of crystals, perovskite oxides with different crystal orientations could differ immensely in terms of the electron distribution and the adsorption/desorption of reaction intermediates [163]. As such, insights into the effects of the crystal orientation on the physiochemical properties of perovskite oxides are quite crucial in advancing the design of perovskite oxides for highly efficient OER. Wokaun et al. reported that ${\mathrm{La}}_{0.6}{\mathrm{Ca}}_{0.4}{\mathrm{CoO}}_{3}$ film with an orientation of (100) possessed a suitable surface energy and showed a higher OER activity than of (110) [164]. This result was further demonstrated by Markovic group, they found that polar ${\mathrm{SrTiO}}_{3}$ (110) and ${\mathrm{SrTiO}}_{3}$ (111) films displayed considerably larger surface energies and provided more sites for the adsorption of ${\mathrm{OH}}^{-}$, consequently exhibiting higher OER activities than non-polar ${\mathrm{SrTiO}}_{3}$ (001) film. However, their OER stability shows the opposite result that ${\mathrm{SrTiO}}_{3}$ (001) film displays a best stability [165]. Moreover, Wu et al. fabricated ${\mathrm{LaCo}}{\mathrm{O}}_{3}$ (LCO) films with different crystal orientations [i.e., (100), (110) and (111)] on ${\mathrm{LaAl}}{\mathrm{O}}_{3}$ (LAO) substrates to investigate the effects of the electronic state of LCO toward OER (Fig. 13a, b); the results illustrated that the LCO (100) film displayed the highest proportion of intermediate spin states of ${\mathrm{Co}}^{3+}$, which endowed it with an optimal ${\mathrm{e}}_{\mathrm{g}}$ filling of ~ 0.87, the highest electrical conductivity and the lowest adsorption energy of intermediate species (Fig. 13c) and consequently exhibited a remarkably better OER catalytic activity than LCO (110) and LCO (111) films in 1.0 M KOH solution [166]. Further doping Sr in LCO (111) could greatly enhance the Co 3d–O 2p hybridization and improve the charge transfer and deprotonation capability, as confirmed by Chai and coworkers [167]. Shortly after, ${\mathrm{Pr}}{\mathrm{Ba}}_{0.5}{\mathrm{Sr}}_{0.5}{\mathrm{Co}}_{1.5}{\mathrm{Fe}}_{0.5}{\mathrm{O}}_{\mathrm{5} + \delta}$ film with an orientation of (100) was prepared, which delivered a faster proton-coupled electron transfer ability, a higher ionic diffusivity and a better performance in deprotonation of ${\mathrm{H}}_{2}{\mathrm{O}}$ to form surface–OH species than the films with lattice orientations of (110) and (111) [168] (Fig. 13d, e). In addition, it is worth noting that crystal orientation determines the OER pathway. For instance, Sun et al. found that (100)-oriented ${\mathrm{NdNiO}}_{3}$ film with a lower formation energy and a higher stability of oxygen vacancies favored the participation of lattice oxygen via the LOM during OER, leading to a better OER performance than (110)- and (111)-oriented ${\mathrm{NdNiO}}_{3}$ films via the AEM (Fig. 13f**)** [169]. Therefore, controlling crystal orientations of perovskite oxides could tailor the polarity, adsorption ability and electronic state through regulating atomic arrangement and coordination environment.

### Lattice Strain

**Fig. 14:**
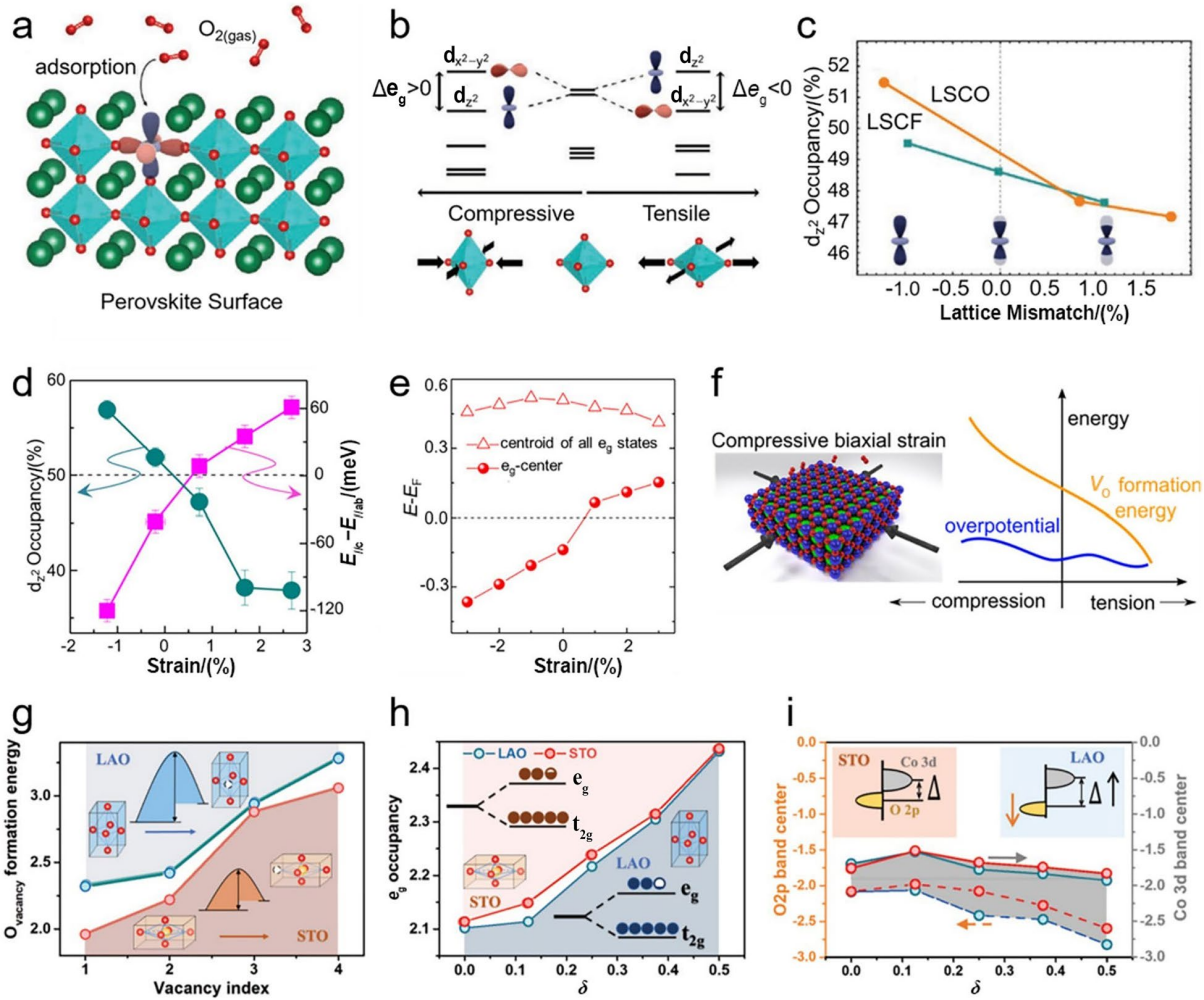
Schematic illustrations of (**a**) OER on perovskite oxide surface, **b** the effects of strain on orbital energies and electron occupancy. **c** The relation between $\mathrm{d}_{{z^{2} }}$ orbital occupancy and the lattice mismatch of ${\mathrm{La}}_{0.5}{\mathrm{Sr}}_{0.5}{\mathrm{Co}}{\mathrm{O}}_{3}$ and ${\mathrm{La}}_{0.8}{\mathrm{Sr}}_{0.2}{\mathrm{Co}}_{0.2}{\mathrm{Fe}}_{0.8}{\mathrm{O}}_{3}$ films. Adapted with permission from Ref. [172]. Copyright © 2021, Wiley–VCH. **d** $\mathrm{d}_{{z^{2} }}$ orbital electron occupancy and the relative energy positions of the $\mathrm{d}_{{z^{2} }}$ orbital compared to the $\mathrm{d}_{{x^{2} {-}y^{2} }}$ orbital along with **e** the position of centroids of ${\mathrm{e}}_{\mathrm{g}}$ states and ${\mathrm{e}}_{\mathrm{g}}\mathrm{-center}$ relative to *E*_F_ as a function of strain in LNO films. Adapted with permission from Ref. [61]. Copyright © 2016, American Chemical Society. **f** *η* and oxygen vacancy formation energy of ${\mathrm{SrCo}}{\mathrm{O}}_{3}$ against the compressive and tensile strains. Adapted with permission from Ref. [174]. Copyright © 2016, American Chemical Society. The plots of g) oxygen vacancy formation energy versus vacancy index, **h** ${\mathrm{e}}_{\mathrm{g}}$ occupancy and **i** O 2p band center versus $\delta$ values of LSC/LAO and LSC/STO. Adapted with permission from Ref. [175]. Copyright © 2019, Wiley–VCH

Normally, two ${\mathrm{e}}_{\mathrm{g}}$ orbitals (i.e., ${\mathrm{d}}_{{{x}}^{2}-{{y}}^{2}}$ and ${\mathrm{d}}_{{{z}}^{2}}$) in a cubic crystal field of unstrained perovskite oxides are energetically degenerate, where electrons are equally distributed, while the lattice strain in perovskite oxides induces the octahedral tilts and rotations, which alter the B–O bond lengths, break the energetic degeneracy of the two ${\mathrm{e}}_{\mathrm{g}}$ orbitals and cause the redistribution of electrons [170]. Moreover, the energy of ${\mathrm{d}}_{{{x}}^{2}-{{y}}^{2}}$ orbital could be reduced by the tensile strain, together with an increase of the electron occupancy in ${\mathrm{d}}_{{{x}}^{2}-{{y}}^{2}}$ orbital and a decrease of that in ${\mathrm{d}}_{{\mathrm{z}}^{2}}$ orbital, while an inverse phenomenon appears for the compressive strain. However, the difficulty to examine the lattice strain challenges its further investigation, thus it is imperative to explore a simple and effective device or system to study the effect of lattice strain on OER performance. Among the progresses made so far in this regard, Shao-Horn et al. firstly demonstrated that the moderate tensile strain could improve the OER/ORR catalytic activity of LCO by optimizing the ${\mathrm{e}}_{\mathrm{g}}$ orbital filling [171]. Following that, Martin et al. also reported that biaxial tensile strain could accelerate the reaction kinetics of ${\mathrm{La}}_{0.5}{\mathrm{Sr}}_{0.5}{\mathrm{Co}}{\mathrm{O}}_{3}$ and ${\mathrm{La}}_{0.8}{\mathrm{Sr}}_{0.2}{\mathrm{Co}}_{0.2}{\mathrm{Fe}}_{0.8}{\mathrm{O}}_{3}$ films toward oxygen electrocatalysis by lowering the electron occupation of the ${\mathrm{d}}_{{{z}}^{2}}$ orbitals (Fig. 14a–c) [172]. More persuasively, Lee group systematically investigated the OER activity trend of ${\mathrm{LaNiO}}_{3}$ (LNO) films by adjusting the strain level from − 2.2 to 2.7% and found that compressive strain could induce a higher occupancy of the ${\mathrm{d}}_{{{z}}^{2}}$ orbitals and a lower energy of ${\mathrm{e}}_{\mathrm{g}}\mathrm{-center}$, which subsequently caused a weak M–O chemisorption and an enhancement in OER activity (Fig. 14d, e) [61]. This phenomenon also appeared in the research of in-plane compressive strain of ${\mathrm{La}}_{0.95}{\mathrm{Ni}}{\mathrm{O}}_{3}$ films, where a higher ${\mathrm{d}}_{{{z}}^{2}}$ orbital occupancy and a suitable M–O bond strength for OER were induced [110]; a further comparative study also reported that 0.2% compressive or tensile strains could move the ${\mathrm{e}}_{\mathrm{g}}\mathrm{-center}$ of LNO membranes toward lower energies, causing a weakened M–O chemisorption and an enhanced charge transfer between LNO and oxygen intermediates [173].

Besides, strain could also regulate the oxygen vacancy formation energy to modify the OER activity. For instance, using ${\mathrm{NdNi}}{\mathrm{O}}_{3}$ as a model, Du et al. found that the compressive strain favored OER by generating an increased occupancy in ${\mathrm{d}}_{{{3}{{z}}}^{2}-{{r}}^{2}}$ orbital and a weak Ni–O chemisorption, and thus, benefiting from the formation of a high concentration of oxygen vacancy, the large enough tension strain could also improve OER catalytic activity [60]. Likewise, Smith et al. reported that a moderate compressive biaxial strain of ${\mathrm{SrCo}}{\mathrm{O}}_{3}$ could raise the energy barrier to form surface oxygen vacancy (Fig. 14f), leading to an optimal concentration of surface oxygen vacancies and thus, maintaining a considerable OER performance [174]. In parallel, Liu et al. found that ${\mathrm{La}}_{0.7}{\mathrm{Sr}}_{0.3}{\mathrm{Co}}{\mathrm{O}}_{{3}-\delta}$ under tensile strain (i.e., LSC/STO) showed a smaller oxygen vacancy formation energy than that under compressive strain (i.e., LSC/LAO) and consequently possessed a larger concentration of oxygen vacancy; they also found that excessive oxygen vacancies on LSC/STO surface aggrandized the ${\mathrm{e}}_{\mathrm{g}}$ state occupancy and enlarged the energy gap between O 2p and Co 3d bands (Fig. 14g–i), resulting in a lower OER performance as compared to LSC/LAO [175].

**Fig. 15:**
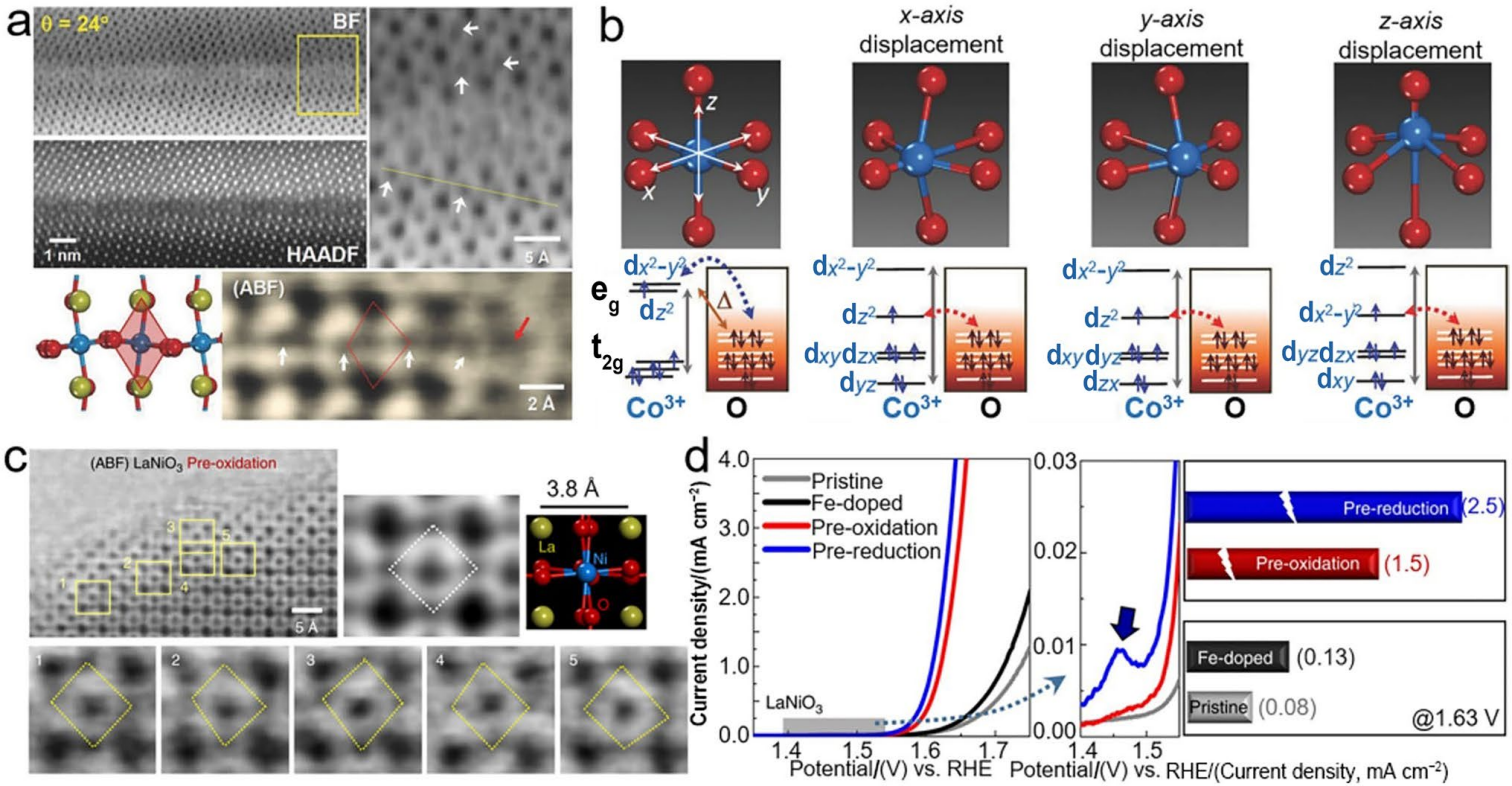
**a** STEM images of ${\mathrm{LaCoO}}_{3}$(001) film showing the grain boundary and the distortion of oxygen octahedral near the grain boundary (below). **b** Schematic illustration of the alternation of Co 3d orbital electron occupancy induced by Co displacements. Adapted with permission from Ref. [176]. Copyright © 2018, Wiley–VCH. **c** STEM images of the displaced oxygen octahedral in ${\mathrm{LaNiO}}_{3}$ films. **d** Comparison of OER catalytic activity of ${\mathrm{LaNiO}}_{3}$ films treated by different methods. Adapted with permission from Ref. [177]. Copyright © 2019, Springer Nature

In addition to tailor the in-plane lattice strain, Chung group paid special attention on the manipulation of the atomic-scale misfit strains of perovskite oxide lattice to improve the OER performance. For example, they employed sintered polycrystalline films as models and found that the surface-terminating grain boundaries in ${\mathrm{La}}{\mathrm{CoO}}_{3}$ and ${\mathrm{La}}{\mathrm{MnO}}_{3}$ achieved over an order of magnitude higher OER activity than the bulk one [176], while the atomically resolved scanning transmission electron microscopy (STEM) analysis confirmed that the atomic arrangement at the grain boundaries was severely displaced, which greatly broke the original symmetry configurations (Fig. 15a). The DFT calculation results indicated that such symmetry-broken atomic displacements could result in an enhanced hybridization between Co ${\mathrm{d}}_{{{x}}^{2}-{{y}}^{2}}$ (or ${\mathrm{d}}_{{{z}}^{2}}$) orbital and O 2p orbitals near the Fermi level, thereby endowing itself with an efficient charge transfer ability between transition metals and oxygen (Fig. 15b). Shortly after, using electrochemical pre-reduction and pre-oxidation methods in a Fe-containing KOH solution, they found that ${\mathrm{Fe}}^{3+}$ from the electrolyte could exchange the Ni sites on the surface region of thin ${\mathrm{LnNi}}{\mathrm{O}}_{3}$ (Ln = La, Pr and Nd) films and cause the strong distortion of oxygen octahedra with different degrees at the angstrom scale, while the samples prepared by Fe doping still maintained their oxygen octahedra undistorted (Fig. 15c) [177]. The DFT calculation results showed that the distortions could tailor the O 2p and Ni/Fe 3d states near the Fermi level, promoting the charge transfer between transition metals and oxygen, and greatly improving the OER catalytic activity (Fig. 15d). In another study, they demonstrated that employing lattice elastic strain in (001) epitaxial thin ${\mathrm{L}}{\mathrm{a}}{\mathrm{Ni}}{\mathrm{O}}_{3}$ films could break the lattice symmetry and lead to the formation of RP faults, which was demonstrated to be highly electroactive toward OER [178]. Comparatively, thin ${\mathrm{L}}{\mathrm{a}}{\mathrm{Ni}}{\mathrm{O}}_{3}$ film with a tensile misfit strain is more favorable for the formation of RP faults than that with a compressive misfit strain and consequently exhibited a better OER catalytic activity. The aforementioned studies have confirmed that tuning electronic structure and oxygen vacancies content through modulating lattice strain of perovskite oxides could accelerate the proton-coupled electron transfer kinetics and the adsorption of oxygenated intermediates during OER, paving a new avenue for the smart design of perovskite oxides.

## Hybrids

Compared with individual perovskite oxide, hybrids of perovskite oxides with other materials [i.e., carbon materials, other perovskite oxides, in situ exsolved metal/alloy (or compounds) NPs and metal-based compounds] could build an interface between two or more components, and reach an optimal electronic structure, thus generating a synergistic effect and delivering a better OER performance to some extent. The synergistic mechanisms of perovskite oxides with other materials are thus systematically elaborated in this section to provide more insights for the scientific community to develop advanced hybrids.

### Carbon Materials

Conventionally, carbon materials have superb electrical conductivity, which could well address the issue of inherently insufficient electrical conductivity of perovskite oxides when utilized as the conductive auxiliary materials to further enhance OER performance [179]. Thus, understanding how carbon materials interact with perovskite oxides and their synergistic contribution to the enhanced performance is crucial to guide the design of perovskite oxides/carbon materials. Simply put, Vulcan XC-72R, Ketjen black, Super P/C65 and acetylene black are commonly used in preparing electrodes, adding these carbon materials in perovskite oxides to form hybrids through a physical mixing method has been widely accepted as an effective strategy to improve the electrical conductivity of electrode and the subsequent OER catalytic activity [12]. In this regard, Morallón et al. demonstrated that the ${\mathrm{La}}{\mathrm{Mn}}_{0.7}{\mathrm{Co}}_{0.3}{\mathrm{O}}_{3}$/carbon hybrids obtained by ball-milling possess a higher interaction than that prepared by mortar and manual shaking, which enriched a faster electron transfer ability, higher OER and ORR catalytic activities [180].

However, many studies have suggested that carbon materials not only act as conductive supports, but also have more beneficial impacts on perovskite oxides. Schmidt et al. explored the influences of adding functionalized acetylene black carbon (AB) on various perovskite oxides [i.e., cubic perovskite oxides of ${\mathrm{La}}_{0.6}{\mathrm{Sr}}_{0.4}{\mathrm{Co}}_{0.8}{\mathrm{Fe}}_{0.2}{\mathrm{O}}_{{3}-\delta}$ and BSCF, and double perovskite oxides of $\mathrm{(}{\mathrm{Pr}}_{0.5}{\mathrm{Ba}}_{0.5}\mathrm{)Co}{\mathrm{O}}_{{3{-}}\delta}$], the results presented that significant improvements of OER catalytic activity were achieved by the addition of AB over cubic perovskite oxides, especially on BSCF, whereas negligible changes were observed on double perovskite oxides, implying the importance of AB and its preferential selection for different perovskite oxides [181]. To further understand the underlying interactions between carbon materials and perovskite oxides, Schmidt and co-workers found that carbon could act as a reducing agent to reduce B-site cations and thus, affect their electronic structure. This was also detected in BSCF/AB, where a reduction of the Co oxidation state occurred, and the tuned Co valence state optimized the electronic structure and improved intrinsic electrical conductivity of BSCF, thereby exhibiting a higher OER catalytic activity as compared to the pure BSCF [182]. More importantly, Co-based perovskite oxides appeared to be the best candidates for tuning the cation electronic structure since no shift was observed in Fe K-edge spectra, indicating that carbon materials cannot affect all B-site cations. Moreover, the electronic structure of BSCF can also be modified by simultaneously ball milling carbon fiber with BSCF [183]. However, the electronic interactions between carbon materials and perovskite oxides mainly depend on the physicochemical properties of carbon materials and the electronic properties of B-site cations. Nevertheless, it is worthy to note that a significant carbon oxidation appeared at high potentials during a long-term test, followed by the formation of carbonate in alkaline solution, which would in turn deteriorate the OER performance [181]. Generally, commercial carbon materials are inevitably corroded at high potentials, and transformed into ${\mathrm{CO}}_{2}$ [(Eq. 4)] and/or ${\mathrm{CO}}_{3}^{{2}-}$ [(Eq. 5)], resulting in the degradation of OER catalytic activity and stability. To address this issue, replacing the commercial carbon materials with graphitized carbon or other high conductive materials is an effective approach to improving the corrosion resistance [12]. For example, Ogumi and co-workers demonstrated that the Sb-doped ${\mathrm{Sn}}{\mathrm{O}}_{2}$ could be used as a conductive substitute to greatly improve the durability and activity of catalysts than commercial carbon [184]. Sato et al. employed graphitized platelet-type carbon nanofibers as conductive additives towards OER and demonstrated a highly resistant ability to carbon corrosion at high anodic potentials [185].

4 $${\text{C }} + {\text{ 4OH}}^{-} { } \to {\text{ CO}}_{{2}} { } + {\text{ 2H}}_{{2}} {\text{O }} + {\text{ 4e}}^{-}$$

5 $${\mathrm{CO}}_{{2}} { } + {\text{ 2OH}}^{-} { } \to {\text{ CO}}_{{3}}^{{{2}{-}}} { } + {\text{ H}}_{{2}} {\mathrm{O}}$$

### Other Perovskite Oxides

**Fig. 16:**
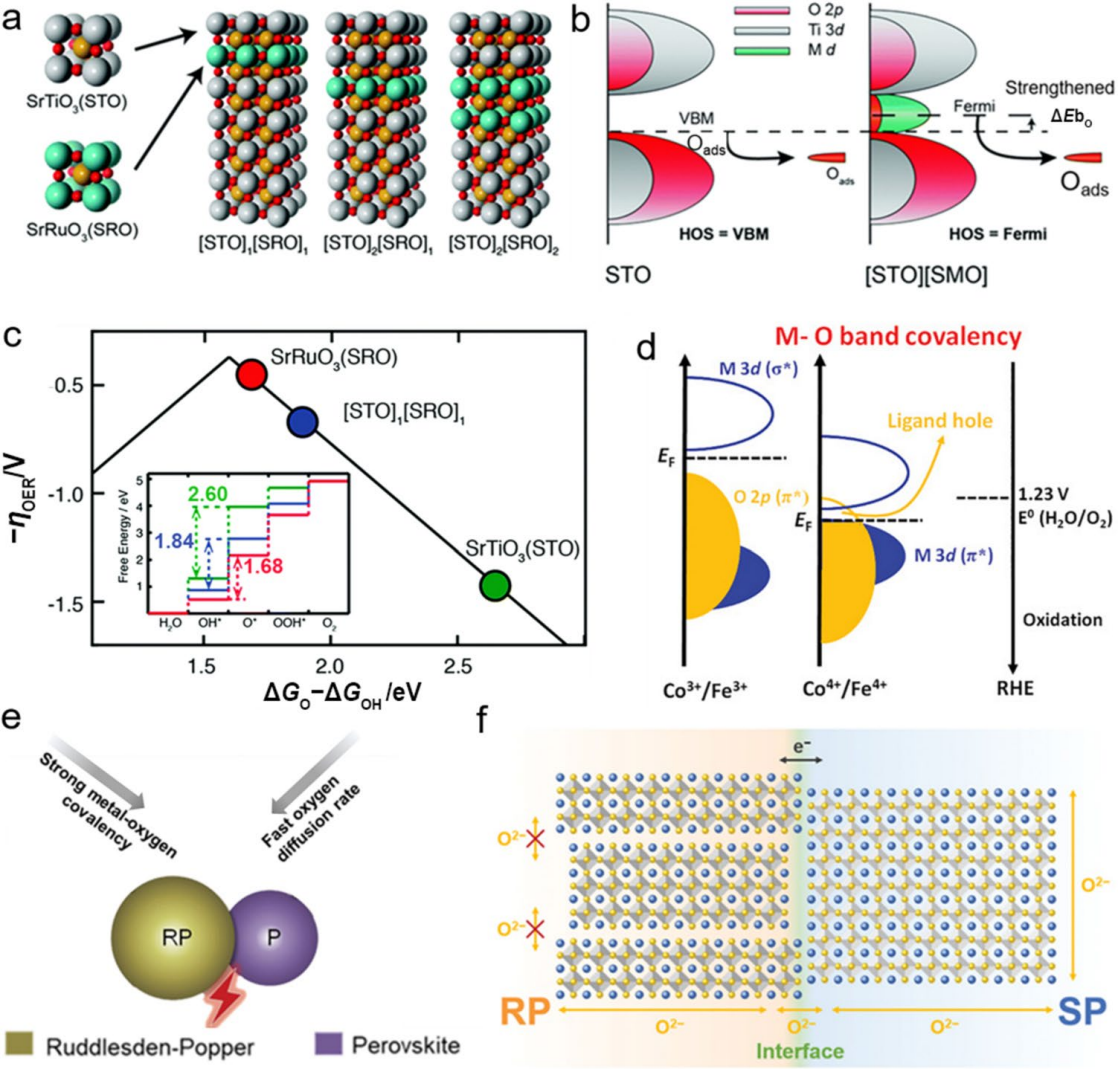
**a** The heterostructures of ${\mathrm{[STO]}}_{{n}}{\mathrm{[SRO]}}_{{m}}$. **b** DOS showing the influences of the buried SRO layer on the surface-adsorbed O and the top STO layer. **c** The DFT calculated OER potentials as a function of the $\Delta G_{{\mathrm{O}}} - \Delta G_{{{\mathrm{OH}}}}$. The inset is the Δ*G* for OER on the surface of SRO (red), STO (green) and ${\mathrm{[STO]}}_{1}{\mathrm{[SRO]}}_{1}$ (blue). Adapted with permission from Ref. [186]. Copyright © 2018, The Royal Society of Chemistry. **d** The enhanced covalency of ${\mathrm{Co}}^{4 + } {\mathrm{/Fe}}^{4 + }$−O bonds in RP/P composites. **e** Schematic illustration of lattice oxygen oxidation triggered by the composite interaction between RP and P. Adapted with permission from Ref. [190]. Copyright © 2020, Wiley–VCH. **f** Schematic illustration of the complementary ${\mathrm{e}}^{-}$ and ${\mathrm{O}}^{{2{-}}}$ transfers processes at the RP/SP interface. Adapted with permission from Ref. [191]. Copyright © 2021, Wiley–VCH

Many studies showed that perovskite oxide-based composites could notably improve OER catalytic activity by virtue of the synergistic effects among different perovskite oxides. For example, Vojvodic et al. reported a layered heterostructure with a specific architecture of core–shell through inserting one-unit cell of ${\mathrm{SrRu}}{\mathrm{O}}_{3}$ (SRO) underneath an ultrathin ${\mathrm{SrTi}}{\mathrm{O}}_{3}$ (STO), which could sufficiently activate the STO for OER. Further inserting two-unit cells of SRO could sufficiently prevent the inherently unstable STO from corrosion under OER conditions due to the interaction between the adsorbates and the shell layer resulting from the optimization of electronic hybridization (Fig. 16a–c) [186]. Besides, Marcel et al. constructed BSCF-${\mathrm{La}}_{0.8}{\mathrm{Sr}}_{0.2}{\mathrm{Mn}}{\mathrm{O}}_{{3}-\delta}$ hybrid on (001)-oriented Nb-doped ${\mathrm{SrTi}}{\mathrm{O}}_{3}$ film through pulsed laser deposition technique, which exhibited enhanced activities and stability for both OER and ORR, relatively better than the state-of-the-art ${\mathrm{Ir}}{\mathrm{O}}_{2}$ and ${\mathrm{Ru}}{\mathrm{O}}_{2}$ [187].

Moreover, Shao group examined a series of self-assembled perovskite oxide composites for OER, e.g., single perovskite oxide/single perovskite oxide (SP/SP), single perovskite oxide/double perovskite oxide (SP/DP) and single perovskite oxide/RP-type perovskite oxide (SP/RP), and these composites were all prepared by one-pot wet-chemistry approach [188–191]. For SP/DP composite (${\mathrm{Sr}}{\mathrm{Fe}}_{0.57}{\mathrm{Co}}_{0.27}{\mathrm{Mo}}_{0.16}{\mathrm{O}}_{2.99}\mathrm{/}{\mathrm{Sr}}_{2}{{\mathrm{Fe}}}_{0.85}{\mathrm{Co}}_{0.17}{\mathrm{Mo}}_{0.56}{\mathrm{Ni}}_{0.42}{\mathrm{O}}_{6}$), an amorphous surface layer would be formed on ${\mathrm{Sr}}{\mathrm{Fe}}_{0.57}{\mathrm{Co}}_{0.27}{\mathrm{Mo}}_{0.16}{\mathrm{O}}_{2.99}$, which could provide more electroactive sites, while ${\mathrm{Sr}}_{2}{{\mathrm{Fe}}}_{0.85}{\mathrm{Co}}_{0.17}{\mathrm{Mo}}_{0.56}{\mathrm{Ni}}_{0.42}{\mathrm{O}}_{6}$ acted as a skeleton to provide an electronic pathway for the amorphous layer, and regulated the amorphization of ${\mathrm{Sr}}{\mathrm{Fe}}_{0.57}{\mathrm{Co}}_{0.27}{\mathrm{Mo}}_{0.16}{\mathrm{O}}_{2.99}$, both of which compromised each other and guaranteed a stable high catalytic activity for OER [189]. For SP/RP composite (${\mathrm{La}}_{0.25}{\mathrm{Sr}}_{0.75}{\mathrm{Co}}_{0.5}{\mathrm{Fe}}_{0.5}{\mathrm{O}}_{3}\mathrm{/La}{\mathrm{Sr}}_{3}{{\mathrm{Co}}}_{1.5}{\mathrm{Fe}}_{1.5}{\mathrm{O}}_{10}$), the higher Co/Fe–O covalency of SP/RP than those of individual SP and RP counterparts could better activate lattice oxygen ions (Fig. 16d, e), and the faster transport of oxygen ions in SP/RP composite could enable a faster refilling of surface lattice oxygen, providing direct evidence of LOM toward OER [190]. More recently, the SP/RP composites (${\mathrm{La}}_{0.33}{\mathrm{Sr}}_{0.67}{\mathrm{Co}}_{0.5}{\mathrm{Fe}}_{0.5}{\mathrm{O}}_{3}\mathrm{/La}{\mathrm{Sr}}_{3}{{\mathrm{Co}}}_{1.5}{\mathrm{Fe}}_{1.5}{\mathrm{O}}_{10}$) with different SP/RP molar ratios were synthesized by directly annealing A-site non-stoichiometric RP precursors at high temperature. The results hinted that the SP/RP composite possessed more strongly interacting interfaces as compared to the physically mixed composite. More persuasively, a further ball-milling treatment would obviously lower the OER catalytic activity due to the damaged interfacial interaction, but a repeated calcination again restored the interfacial effect and the associated OER performance. A further study focused its emphasis on the strong coupling between SP and RP, which offsets the weakness of ${\mathrm{O}}^{2{-}}$ transfer along *c* direction on RP and finally promotes a better participation of lattice oxygen during OER (Fig. 16f) [191]. Upon using a one-pot wet-chemistry approach, Duan et al. found that the preparation of the targeted ${\mathrm{La}}_{{x}}{{\mathrm{Sr}}}_{\mathrm{4} - {{x}}}{\mathrm{Ni}}_{{y}}{{\mathrm{Fe}}}_{\mathrm{3} - {{y}}}{\mathrm{O}}_{\mathrm{10} - \delta}$ samples could undergo phase separation to form a composite composed of one SP phase and two RP phases. Among them, a relatively uniform distribution of SP and RP phases when synthesizing ${\mathrm{La}}_{2}{{\mathrm{Sr}}}_{2}{{\mathrm{Ni}}}_{1}{{\mathrm{Fe}}}_{2}{{\mathrm{O}}}_{\mathrm{10} - \delta}$ was obtained by alternating the cations stoichiometry, greatly improving the oxygen ion diffusion rate, and consequently leading to an enhanced OER performance [192]. It is clear that all the composites exhibited better OER performances as compared to their counterparts, persuasively confirming the effectiveness of constructing perovskite oxide-based composites.

### In situ Exsolution

**Fig. 17:**
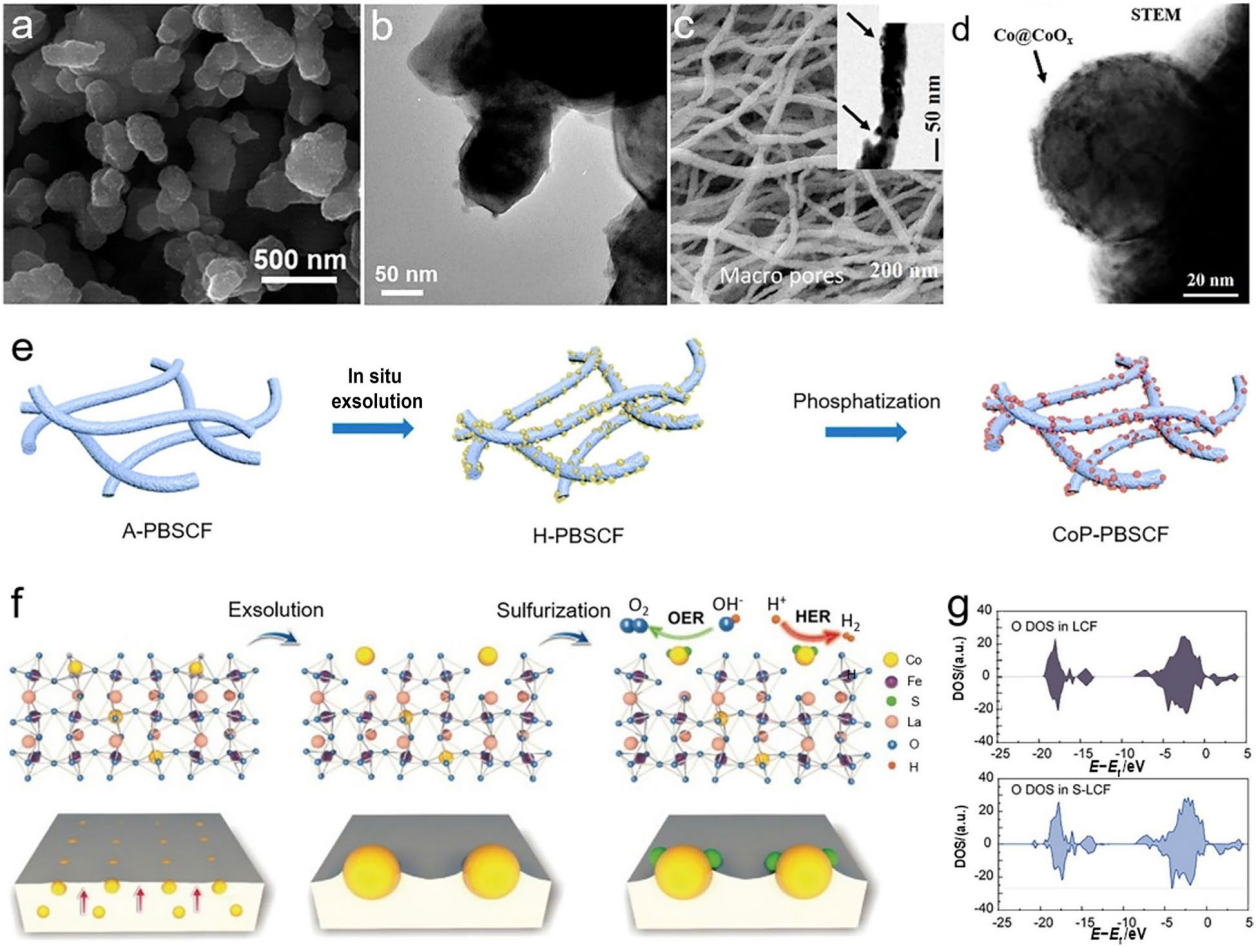
**a** SEM and **b** TEM images of ${\mathrm{Sr}}_{{2}} {\mathrm{Fe}}_{{{1}{\mathrm{.3}}}} {\mathrm{Ni}}_{{{0}{\mathrm{.2}}}} {\mathrm{Mo}}_{{{0}{\mathrm{.5}}}} {\mathrm{O}}_{{6{-}\delta }}$ annealed at 700 °C in 5% ${\mathrm{H}}_{{2}} /{\mathrm{Ar}}$. Adapted with permission from Ref. [194]. Copyright © 2017, The Royal Society of Chemistry. **c** SEM and TEM images of the as-prepared mesoporous nanofibers. **d** Scanning TEM image showing the ${\mathrm{Co@CoO}}_{x}$ NPs on ${\mathrm{(PrBa}}_{{{0}{\mathrm{.8}}}} {\mathrm{Ca}}_{{{0}{\mathrm{.2}}}} {)}_{{{0}{\mathrm{.95}}}} {\mathrm{(Co}}_{{{1}{\mathrm{.5}}}} {\mathrm{Fe}}_{{{0}{\mathrm{.5}}}} {)}_{{{0}{\mathrm{.95}}}} {\mathrm{Co}}_{{{0}{\mathrm{.05}}}} {\mathrm{O}}_{{{5} + \delta }}$ nanofibers. Adapted with permission from Ref. [196]. Copyright © 2017, Elsevier Ltd. The formation processes of **e** CoP-PBSCF and **f** S-LCF. **g** The calculated O DOS in LCF and S-LCF. Adapted with permission from Ref. [198, 200]. Copyright © 2019, The Royal Society of Chemistry; Copyright © 2020, Elsevier Ltd.

Generally, B-site cations (e.g., Fe, Co and Ni) energetically prefer to be reduced and then in situ exsolved from the bulk lattice with the oxygen escaping from the corresponding sites under reducing conditions as compared to the inert A-site cations (e.g., La, Sr and Ba) [193]. More importantly, perovskite oxides with homogeneously distributed metal/alloy NPs synthesized by in situ exsolving A/B-site cations from perovskite oxide backbone have shown comparably high OER performances. For example, Yang et al. annealed double perovskite oxides of ${\mathrm{Sr}}_{2}{{\mathrm{Fe}}}_{1.3}{\mathrm{Ni}}_{0.2}{\mathrm{Mo}}_{0.5}{\mathrm{O}}_{{6}-\delta}$ at 700 °C in different atmospheres (i.e., Ar, ${\mathrm{H}}_{2}$/Ar and ${\mathrm{H}}_{2}$), where the one treated in ${\mathrm{H}}_{2}\mathrm{/Ar}$ possessed rich oxygen vacancies and its surface was uniformly covered by in situ exsolved FeNi alloy NPs (Fig. 17a, b), resulting in a greatly increased OER catalytic activity [194]. Following this, they further demonstrated that in situ exsolving Fe NPs from ${\mathrm{SrTi}}_{0.8}{\mathrm{Fe}}_{0.2}{\mathrm{O}}_{{3{-}}\delta}$ could increase oxygen vacancies concentration and expose more active sites, both of which facilitate the adsorption of OH^‒^ and greatly lower the required energy to drive a desirable OER [195]. Equivalently, the mesoporous ${\mathrm{(}{\mathrm{Pr}}{\mathrm{Ba}}_{0.8}{\mathrm{Ca}}_{0.2}\mathrm{)}}_{0.95}{\mathrm{(}{\mathrm{Co}}_{1.5}{\mathrm{Fe}}_{0.5}\mathrm{)}}_{0.95}{\mathrm{Co}}_{0.05}{\mathrm{O}}_{\mathrm{5} + \delta}$ nanofibers with in situ formed $\mathrm{Co@}{\mathrm{CoO}}_{{x}}$ NPs (Fig. 17c, d) also achieved notably higher OER catalytic performance than BSCF, ${\mathrm{IrO}}_{2}$ and ${\mathrm{RuO}}_{2}$ in alkaline solutions due to the rich oxygen vacancies, the synergistic effects of dual components and the large specific surface area [196].

As research progressed, Ru NPs were first in situ exsolved on the surface of ${\mathrm{La}}_{0.9}{\mathrm{Fe}}_{0.92}{\mathrm{Ru}}_{\mathrm{0.08}-{{x}}}{\mathrm{O}}_{{3{-}}\delta}$, followed by an oxidation, to form a composite of ${\mathrm{RuO}}_{2}\mathrm{/}{\mathrm{La}}_{0.9}{\mathrm{Fe}}_{0.92}{\mathrm{Ru}}_{{0.08{-}}{{x}}}{\mathrm{O}}_{{3{-}}\delta}$, and it is found that the formed ${\mathrm{RuO}}_{2}$ on ${\mathrm{La}}_{0.9}{\mathrm{Fe}}_{0.92}{\mathrm{Ru}}_{0.08}{\mathrm{O}}_{{3{-}}\delta}$ and the increased electrical conductivity collectively contributed to an enhanced OER catalytic activity [197]. Following this, a further study was carried out on $\mathrm{CoP-Pr}{\mathrm{Ba}}_{0.5}{\mathrm{Sr}}_{0.5}{\mathrm{Co}}_{1.5}{\mathrm{Fe}}_{0.5}{\mathrm{O}}_{\mathrm{5} + \delta}$ (CoP-PBSCF) nanofibers prepared by a two-step process involving an in situ exsolution of Co NPs and a subsequent phosphatization (Fig. 17e). An intimate integration between CoP NPs and PBSCF yielded substantial amounts of synergistically active sites where the CoP NPs could tailor the electronic structure of CoP-PBSCF, accelerate ion and electron transfers, and collectively contribute to an enhanced OER catalytic activity [198]. In a similar approach, the in situ formed ${\mathrm{Ni}}_{2}{\mathrm{P}}$ NPs on ${\mathrm{La}}_{0.8}{\mathrm{Sr}}_{0.2}{\mathrm{Cr}}_{0.69}{\mathrm{Ni}}_{0.31}{\mathrm{O}}_{{3}-\delta}$ greatly enhanced the intrinsic activity by ~ 6 times and the mass activity by ~ 10 times due to an improved electrical conductivity and an optimized O 2p band center position [199]. In addition, to avoid a decrease in electronic conductivity caused by the oxidation of the in situ exsolved metallic NPs under an anodic potential prior to OER, Sun et al. integrated ${\mathrm{Co}}{\mathrm{S}}_{2}$ NPs on ${\mathrm{La}}{\mathrm{Fe}}_{0.8}{\mathrm{Co}}_{0.2}{\mathrm{O}}_{3}$ (LCF) through a consecutive in situ exsolution of Co NPs and a post-sulfurization process (Fig. 17f), where the as-prepared composite possessed abundant electroactive sites, an optimal electronic structure and rich oxygen vacancies (Fig. 17g), all of which contributed to faster ion and charge migration abilities [200]. Through employing the operando XAS tracking technique, Liu et al. found that the in situ exsolved Co and Fe NPs on ${\mathrm{La}}{\mathrm{Co}}_{0.8}{\mathrm{Fe}}_{0.2}{\mathrm{O}}_{{3}-\delta}$ transformed into amorphous $\mathrm{(Co/Fe)O(OH)}$ with an unsaturated coordination of Fe and Co ions under harsh OER condition, and delivered a *j* of 10 mA cm^−2^ at a low *η* of 293 mV [201].

Except for in situ exsolving metal/alloy NPs from B-site of perovskite oxides, some electroactive metal NPs (e.g., Ag and Ni) could be also in situ exsolved from A-site when electrocatalytically inert cations (e.g., Ti, Mn) occupy the B-site [202, 203]. Typically, Pu et al. prepared ${\mathrm{Pr}}_{0.9}{\mathrm{Ag}}_{0.5}{\mathrm{Ba}}_{0.5}{\mathrm{Sr}}_{0.5}{\mathrm{Co}}_{2}{{\mathrm{O}}}_{\mathrm{5} + \delta}$ nanofibers and exsolved well-distributed Ag NPs in situ from A-site; the results suggested that the abundant oxygen vacancies on the surface and the strong interfacial interaction between Ag NPs and the perovskite oxide backbone could jointly facilitate the charge transfer and lower the energy barrier in deprotonation of *OH to *O [204]. This is further confirmed by the in situ exsolved Ni NPs from the A-site of ${\mathrm{CaTi}}{\mathrm{O}}_{3}$, where the strong metal–perovskite oxide interfacial interaction greatly enhanced the OER performance [203]. Clearly, the in situ exsolutions of metal/alloy NPs from A-site (e.g., Ag and Ni) and B-site (e.g., Fe, Co and Ni) of perovskite oxides are of prime significance in guiding the intellectual design of high-performance perovskite oxides capable of efficiently driving OER.

### Metal-Based Compounds

**Fig. 18:**
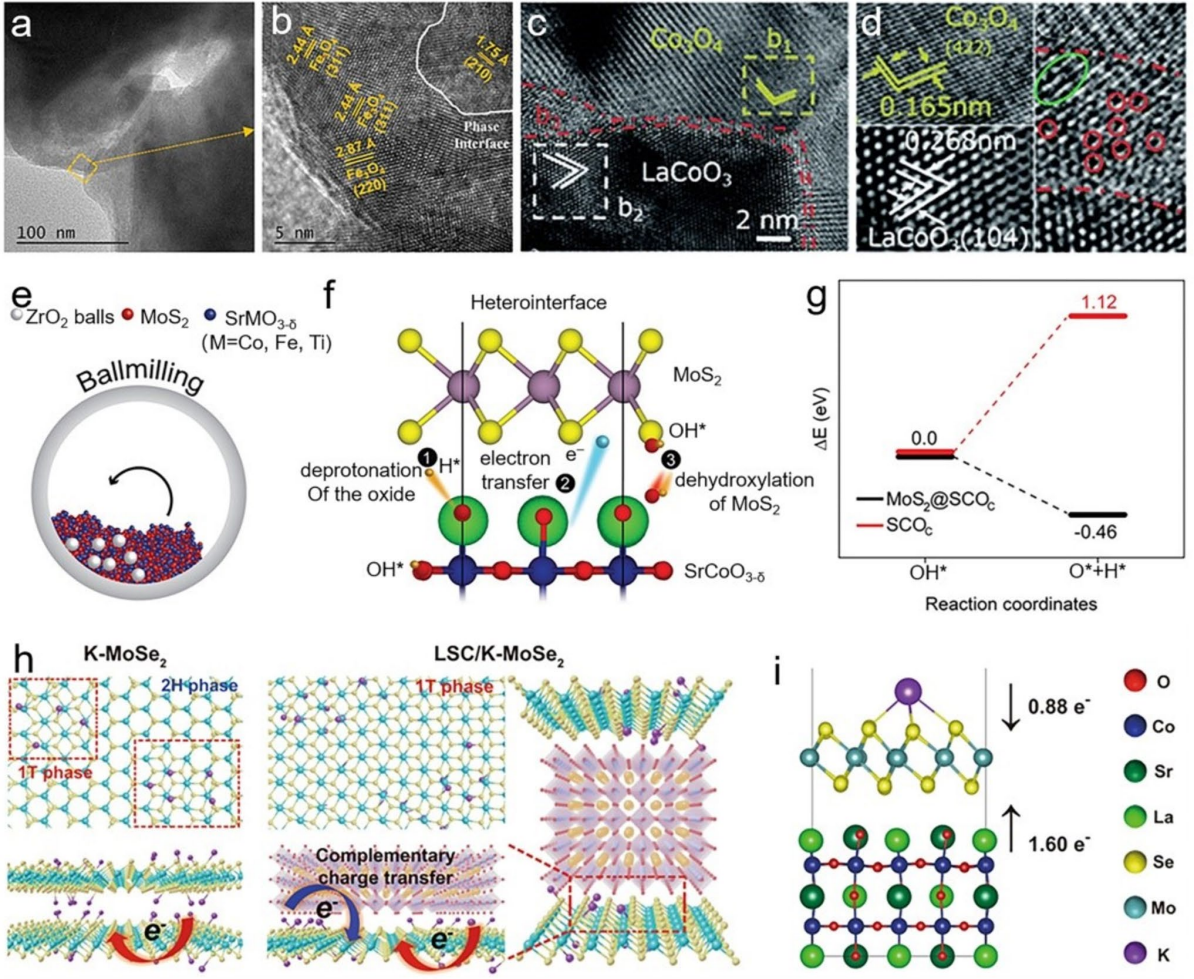
TEM images of (**a**, **b**) ${\mathrm{Fe}}_{3}{{\mathrm{O}}}_{4}$/${\mathrm{SrCo}}_{{{0}{\mathrm{.8}}}} {\mathrm{Fe}}_{{{0}{\mathrm{.2}}}} {\mathrm{O}}_{{3{-}\delta }}$ heterostructure and **c**, **d** ${\mathrm{Co}}_{3}{{\mathrm{O}}}_{4}\mathrm{/LaCo}{\mathrm{O}}_{3}$ heterostructure. Adapted with permission from Ref. [207, 208]. Copyright © 2021, American Chemical Society; Copyright © 2022, The Royal Society of Chemistry. **e** The preparation of ${\mathrm{Mo}}{\mathrm{S}}_{2}$/${\mathrm{SrCoO}}_{{3{-}\delta }}$ heterostructure with a ball-milling method. **f** Schematic illustration of the improved deprotonation, electron transfer and dihydroxylation by incorporating ${\mathrm{Mo}}{\mathrm{S}}_{2}$ in ${\mathrm{SrCoO}}_{{3{-}\delta }}$. **g** The differences of adsorption energies of the ${\text{OH* }} \to {\text{ O* + H*}}$ step on ${\mathrm{SrCoO}}_{{3{-}\delta }}$ and ${\mathrm{MoS}}_{{2}}$/${\mathrm{SrCoO}}_{{3{-}\delta }}$. Adapted with permission from Ref. [211]. Copyright © 2021, Wiley–VCH. **h** Schematic illustrations of the atomic structure and the charge transfer effect for $\mathrm{K} - {\mathrm{Mo}}{\mathrm{Se}}_{2}$ and $\mathrm{K} - {\mathrm{Mo}}{\mathrm{Se}}_{2}\mathrm{/}{\mathrm{La}}_{0.5}{\mathrm{Sr}}_{0.5}{\mathrm{O}}_{{3{-}}\delta}$ composites. **i** Charge transfer from K and ${\mathrm{La}}_{0.5}{\mathrm{Sr}}_{0.5}{\mathrm{Co}}{\mathrm{O}}_{{3{-}}\delta}$ to ${\mathrm{Mo}}{\mathrm{Se}}_{2}$ in an optimal $\mathrm{K} - {\mathrm{Mo}}{\mathrm{Se}}_{2}\mathrm{/}{\mathrm{La}}_{0.5}{\mathrm{Sr}}_{0.5}{\mathrm{CoO}}_{{3{-}}\delta}$ heterostructure. Adapted with permission from Ref. [212]. Copyright © 2021, Springer Nature

Transition metal-based materials have long been the center of research for promoting OER in the past few decades, especially Co-, Ni- and Fe-based oxides/hydroxides [17, 19, 205]. The composites containing perovskite oxides and these transition metal-based compounds have also been demonstrated to have superb OER performance. For example, Ciucci et al. fabricated a core–shell structured CoNi/BSCF/N-doped-C which delivered a *j* of 10 mA cm^−2^ at an *η* of 220 mV on account of the facilitated charge transfer derived from the interfacial interactions between the different components and the electroactive M–OOH/M–OH, originated from the bimetallic CoNi NPs transformation on the surface of BSCF under OER condition [206]. Cui et al. reported that incorporating ${\mathrm{Fe}}_{3}{{\mathrm{O}}}_{4}$ with ${\mathrm{SrCo}}_{0.8}{\mathrm{Fe}}_{0.2}{\mathrm{O}}_{{3}-\delta}$ could enhance the electrical conductivity, increase the Co oxidation state and activate the surface oxygen to ${\mathrm{O}}^{2{-}}\mathrm{/}{\mathrm{O}}^{-}$ species for efficient OER (Fig. 18a, b) [207]. Afterwards, a ${\mathrm{Co}}_{3}{{\mathrm{O}}}_{4}\mathrm{/LaCo}{\mathrm{O}}_{3}$ heterostructure was fabricated with rich oxygen vacancies, increased active sites and accelerated charge transfer rate, which exhibited excellent performances for both OER and ORR (Fig. 18c, d) [208]. Moreover, a self-assembled nanorod-like ${\mathrm{SrCo}}_{0.55}{\mathrm{Fe}}_{0.5}{\mathrm{O}}_{{3{-}}\delta}$ composite composed of a corner-sharing ${\mathrm{SrCo}}_{0.5}{\mathrm{Fe}}_{0.5}{\mathrm{O}}_{{3{-}}\delta}$ and an edge-sharing ${\mathrm{Co}}_{3}{{\mathrm{O}}}_{4}$ was synthesized through a phase separation strategy induced by the excessive introduction of B-site cation. The corner-sharing phase could facilitate the electron transfer, while the edge-sharing phase could promote the deprotonation of *OH during the LOER process, both of which collaboratively reached a double win situation and enabled a superior OER performance, i.e., affording a low *η* of 290 mV to deliver a *j* of 10 mA cm^−2^ in 1.0 mol L^−1^ KOH [209]. Followed by this, Shao et al. prepared a hybrid composed of ${\mathrm{Sr}}_{0.8}{\mathrm{Co}}_{0.8}{{\mathrm{Fe}}_{0.2}{\mathrm{O}}}_{\mathrm{3} - \delta}$ and ${\mathrm{Sr}}_{3}{{\mathrm{B}}}_{2}{{\mathrm{O}}}_{3}$ via a facile sol-gel method, where the ${\mathrm{Sr}}_{3}{{\mathrm{B}}}_{2}{{\mathrm{O}}}_{3}$ as a proton acceptor would promote the interfacial proton transfer, eliminate the inherent kinetic barrier for the rate-determining step and facilitate the formation of O–O bonds during OER. More importantly, the introduction of ${\mathrm{Sr}}_{3}{{\mathrm{B}}}_{2}{{\mathrm{O}}}_{3}$ has been demonstrated as a universally applicable strategy to promote the OER performance for numerous perovskite oxides with O 2p band center close to Fermi level, e.g., ${\mathrm{La}}_{0.4}{\mathrm{Sr}}_{0.6}{\mathrm{Co}}{\mathrm{O}}_{\mathrm{3} - \delta}$, $\mathrm{(}{\mathrm{Pr}}_{0.5}{\mathrm{Ba}}_{0.5}\mathrm{)Co}{\mathrm{O}}_{\mathrm{3} - \delta}$, BSCF and ${\mathrm{Sr}}{\mathrm{Co}}_{0.8}{\mathrm{Fe}}_{0.2}{\mathrm{O}}_{\mathrm{3} - \delta}$ [210].

Other than the oxides and hydroxides, perovskite oxides with transition metal sulfides, phosphides and other transition metal compounds have also been extensively studied. As a representative, incorporating ${\mathrm{Mo}}{\mathrm{S}}_{2}$ with ${\mathrm{SrCo}}{\mathrm{O}}_{{3{-}}\delta}$ obtained a 10-fold higher mass activity, and a largely lowered Tafel slope of only 37 ${\mathrm{mV}}{\text{ dec}}^{-{1}}$, since ${\mathrm{Mo}}{\mathrm{S}}_{2}$ promoted the *OH deprotonation, i.e., the rate-determining step, at the heterointerface during OER (Fig. 18e–g) [211]. A further study demonstrated that mixing $\mathrm{K} - {\mathrm{Mo}}{\mathrm{Se}}_{2}$ with ${\mathrm{La}}_{0.5}{\mathrm{Sr}}_{0.5}{\mathrm{O}}_{{3}-\delta}$ via ball milling could enhance the electrical conductivity and the adsorption capability of oxygen intermediates (e.g., *O, *OH, *OOH) (Fig. 18h, i) [212]. Added to the above is the coupling of Co–Pi with Sr-doped ${\mathrm{La}}_{0.7}{\mathrm{Sr}}_{0.3}{\mathrm{Mn}}{\mathrm{O}}_{3}$, the resultant materials delivered an extremely low *η* of 220 mV at 10 mA cm^−2^ and a Tafel slope of 62 ${\mathrm{mV}}{\text{ dec}}^{-{1}}$, benefited by the enhanced surface kinetics caused by a rapid shuttling of Co cations between + 2 and + 3/ + 4 states in Co phosphate (Co–Pi) [213]. Apparently, assembling metal-based compounds with perovskite oxides enables a strong synergistic interaction, which collectively tunes the electronic structure and accelerates the proton-coupled electron transfers toward efficient OER.

## Other Representative Strategies

**Fig. 19:**
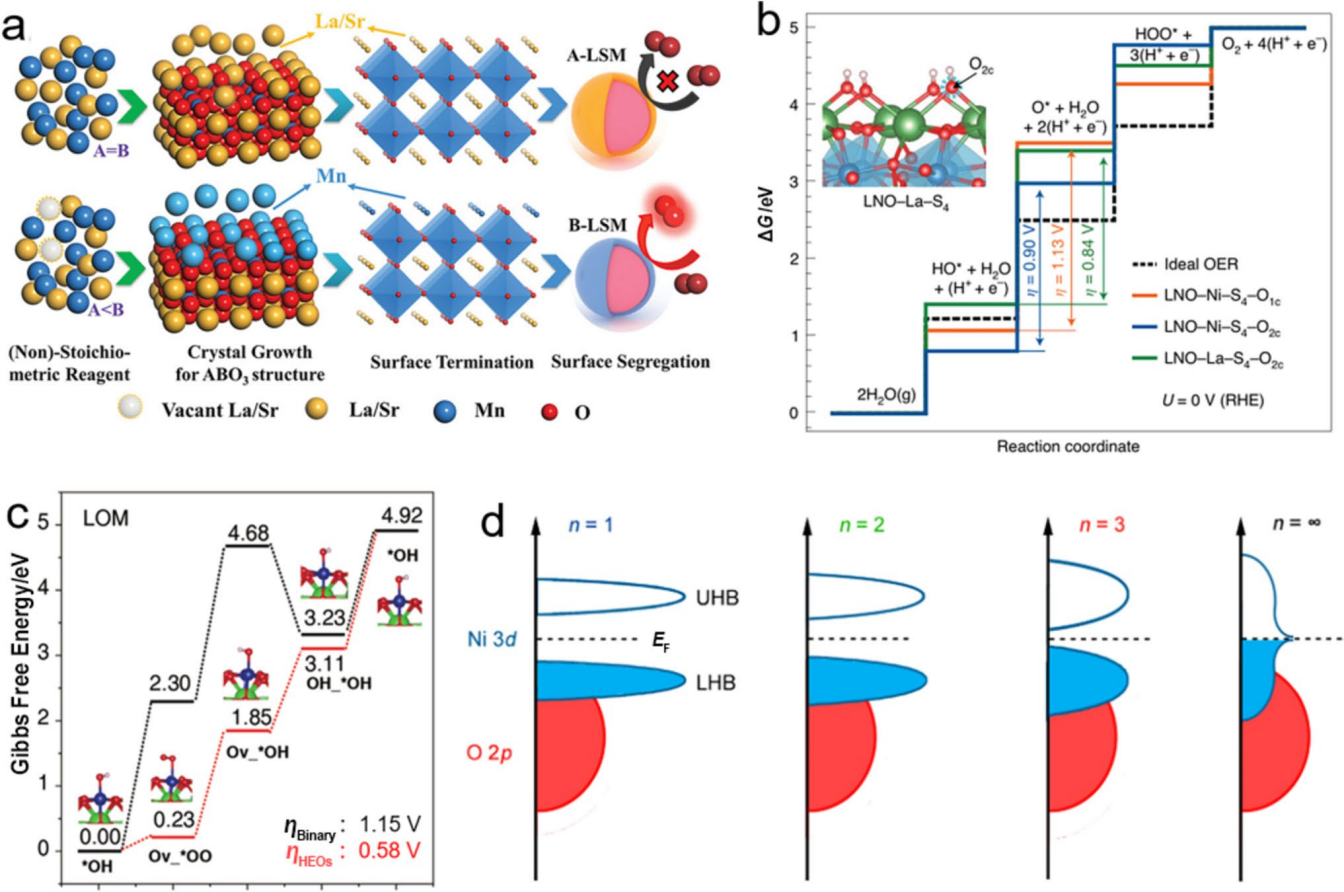
**a** The manipulation of the surface termination of ${\mathrm{La}}_{0.4}{\mathrm{Sr}}_{0.4}{\mathrm{Mn}}{\mathrm{O}}_{3}$. Adapted with permission from Ref. [214]. Copyright © 2021, Wiley–VCH. **b** The calculated Δ*G*s of ${\mathrm{LaNi}}{\mathrm{O}}_{3}$ films with different surface terminations toward OER. Adapted with permission from Ref. [215]. Copyright © 2021, Springer Nature. **c** The calculated ΔGs of high entropy La_0.6_Sr_0.4_Co_0.2_Fe_0.2_Mg_0.2_Ni_0.2_Mn_0.2_O_3_ and La_0.6_Sr_0.4_Co_0.2_Mn_0.8_O_3_ toward OER via a LOM pathway. Adapted with permission from Ref. [217]. Copyright © 2022, Wiley–VCH. **d** The hybridization of O 2p and Ni 3d orbitals of ${\mathrm{La}}_{{n}}{\mathrm{Sr}}{\mathrm{Ni}}_{{n}}{{\mathrm{O}}}_{{3}{{n}}+ {1} }$ (*n* = 1, 2, 3 and ∞, respectively). Adapted with permission from Ref. [214]. Copyright © 2020, American Chemical Society

In addition to the conventional design strategies for perovskite oxides, some representative but rarely reported strategies could also significantly improve the OER performance. For example, tuning the surface composition of perovskite oxides to expose certain surface termination at an atomic-scale holds great potential to effectively enhance the OER performance. Feng et al. reported that the Mn-terminated ${\mathrm{La}}_{0.45}{\mathrm{Sr}}_{0.45}{\mathrm{Mn}}{\mathrm{O}}_{3}$ could maximize the exposure of active B-site cations at the surface, optimize the hybridization of Mn 3d and O 2p orbitals and promote the activation of surface lattice oxygen (Fig. 19a) [214]. Furthermore, Chueh et al. demonstrated that different surface cation terminations in epitaxial ${\mathrm{LaNi}}{\mathrm{O}}_{3}$ films could determine whether the outer atomic layer at the surface experiences a structure transformation during OER. Typically, surface Ni-terminated perovskite oxide could undergo a surface transformation to form a Ni oxyhydroxide-type layer, which is highly electroactive and could lower the *η* toward OER by up to 150 mV at 1 mA cm^−2^ as compared to the surface La-terminated perovskite oxide (Fig. 19b) [215]. Besides, the development of high entropy materials offers broad possibilities for tailoring the structural and/or electronic configurations of perovskite oxides toward OER through manipulating the configurational entropy. For the first time, Ting group prepared a series of high entropy perovskite oxides consisted of at least five different transition metal oxides with similar cationic radii at B-site (e.g., Cr, Mn, Fe, Co and Ni) using a facile co-precipitation method, and the results showed that the high entropy perovskite oxides could catalyze OER via AEM pathways and display higher OER catalytic activities than the single perovskite oxides [216]. Furthermore, after changing the stoichiometry of B-site metals of $\mathrm{La(CrMnFe}{\mathrm{Co}}_{2}\mathrm{Ni)}{\mathrm{O}}_{3}$ with the largest ${\mathrm{Co}}^{3+}\mathrm{/}{\mathrm{Co}}^{2+}$ ratio could display an optimal OER performance, delivering a lowest *η* of 325 mV at a *j* of 10 mA cm^−2^ and an outstanding electrochemical OER stability of 50 h. Dissimilarly, Sun et al. reported that the high entropy perovskite oxide of ${\mathrm{La}}_{0.6}{\mathrm{Sr}}_{0.4}{\mathrm{Co}}_{0.2}{\mathrm{Fe}}_{0.2}{\mathrm{Mg}}_{0.2}{\mathrm{Ni}}_{0.2}{\mathrm{Mn}}_{0.2}{\mathrm{O}}_{3}$ promoted OER via a LOM pathway but only afforded an *η of* 320 mV to deliver a *j* of 10 mA cm^−2^. The DFT calculation results indicated that the high configuration entropy could result in a greatly random occupation of B-site cations, which increased the electrical conductivity, facilitated the formation of surface oxygen vacancies and activated the lattice oxygen for ${\mathrm{O}}_{2}$ evolution (Fig. 19c) [217]. With one step further, high entropy perovskite oxide–halide solid solutions [218] and high entropy perovskite fluorides [219] have also been developed as catalysts to boost OER, and they all exhibited excellent OER performances. These results persuasively demonstrated the great potential of high entropy perovskite oxides toward heterogeneous electrocatalysis. In the meantime, the structural dimensionality of RP-type perovskite oxides has also been investigated for OER. Zeng et al. found that the hybridization of Ni 3d–O 2p orbitals became stronger as the dimensionality (i.e., n) of ${\mathrm{La}}_{{n}}{\mathrm{Sr}}{\mathrm{Ni}}_{{n}}{{\mathrm{O}}}_{{3}{{n}}+{1} }$ (*n* = 1, 2, 3 and ∞) increased, accompanied by a transition from insulator to metal (Fig. 19d). This greatly improved the electrical conductivity and Ni–O covalency of ${\mathrm{La}}_{{n}}{\mathrm{Sr}}{\mathrm{Ni}}_{{n}}{{\mathrm{O}}}_{{3}{{n}}+ {1} }$, and eventually promoted the OER kinetics [220]. However, Nie et al. obtained a different conclusion that the ${\mathrm{La}}_{{n+1}}{\mathrm{Ni}}_{{n}}{{\mathrm{O}}}_{{3}{{n}}+ {1} }$ with a *n* = 5, i.e., ${\mathrm{La}}_{6}{{\mathrm{Ni}}}_{5}{{\mathrm{O}}}_{16}$, possessed an optimal Ni 3d–O 2p orbitals hybridization and ${\mathrm{e}}_{\mathrm{g}}$ occupancy, enabling a relatively higher OER activity than others [221]. Clearly, all the involved design strategies also offer attractive avenues for the construction of perovskite oxides toward OER.

It is worth noting that altering the environmental factors, e.g., tuning the pH of electrolyte [222], adding active cations to the electrolyte [46], employing light irradiation treatment [223], applying a magnetic field [224] and increasing the electrolyte temperature [225], has also provided distinctive and novel insights into the enhancements of OER performances. However, this review particularly focuses on the smart design of perovskite oxides, thus a summary of tuning environment factors toward OER is not within the scope of this paper.

## Conclusions and Perspectives

In the past decades, tremendous efforts, both experimental and theoretical, have been taken to improve the OER performance of perovskite oxides from multiple directions with varying degrees of success. Ample information is available in the literatures in regards to many intellectual design strategies which can alleviate the barriers that hinder or weaken the OER catalytic activity and stability on perovskite oxides, so as to eventually enable the commercial utilization of perovskite oxide-based materials for efficient OER. Herein, five main aspects, as listed below, are systematically and comprehensively summarized in terms of their impacts on physiochemical properties such as electronic structure, crystal structure, oxygen vacancy concentration, specific surface area and hydrophilicity, all of which are demonstrated to effectively promote OER performances with varying degrees. The five aspects are (1) synthetic modulation, (2) doping (i.e., electron tuning, structure distortion, oxygen vacancy and cation deficiency), (3) surface engineering (i.e., size tuning, morphology control, amorphization and surface modification), (4) structure mutations (i.e., phase transition, crystal orientation and lattice strain), (5) hybrids [perovskites with carbon materials, other perovskite oxides, in situ exsolved metal/alloy (or compounds) NPs and metal-based compounds]. Notably, perovskite oxides have also been extensively investigated in many other electrochemical devices, e.g., those for ORR, HER, ${\mathrm{C}}{\mathrm{O}}_{2}$ and ${\mathrm{N}}_{2}$ reduction reactions. Therefore, developing high-performance perovskite oxides capable of effectively driving the associated electrochemical energy storage and conversion devices is of great significance in pursuit of reaching a sustainable development and realizing the ultimate goal of global “carbon emissions peak” and “carbon neutrality”. The abovementioned five aspects of intellectual design strategies can highlight the pathways towards cutting-edge research for the scientific community to develop the perovskite oxides and their derivatives with desirable performances.

Despite significant progress having been made, various issues still remain with the perovskite oxides developed so far, which impede their large-scale applications. Therefore, future studies should be mainly focused on the exploration of highly active and stable perovskite oxides with specific emphasis on solving those issues in preparation for future commercial utilizations of perovskite oxide-based materials for OER. More specifically, the issues and challenges that the technology should address in the future are suggested as follows:

- The structures, dopants and composition of perovskite oxides have crucial impacts on the electronic structure and the intrinsic catalytic activity. In particular, given the better tunability of structures, dopants and composition as well as higher diffusion and surface exchange coefficients, the layered perovskite oxides, as alternatives, hold the greater possibilities to display considerably higher OER performances than cubic ones. Thus, it is imperative to comprehensively uncover the inter-relationships among the structure–, dopant–and composition–related catalytic activities and stabilities of perovskite oxides and to clarify the importance and the role of each part. To this end, an integration of two or more strategies with their own clarified benefits would open up doors for countless opportunities in designing high-performance perovskite oxides without apparent weaknesses, which could be achieved by reaching an optimal balance via mutual compensations.
- Perovskite oxides catalyze OER via different pathways (i.e., AEM and LOM) could result in significant differences in their activity and stability. However, there are still intense debates on what decides the difference and how it works, solving these issues is thus critical for future design of perovskite oxides. Based on the current understanding of the OER mechanisms toward perovskite oxides, there exist intellectual design principles for the purpose of improving their OER performance. To be specific, the scaling relation among OER intermediates in AEM indicates a cap on the OER activity; thus future efforts toward the design of perovskite oxides may focus on the optimizations of electronic structure and the adsorption energies of oxygenated intermediates. In the meantime, perovskite oxides with a LOM during OER could break the inherent limitation caused by the adsorption energy scaling relationship, and provide a possible route for a potential breakthrough in OER activity at an expense of the surface structural stability of perovskite oxides. Therefore, to stabilize the bulk lattice oxygen through optimizing the atomic coordination configurations of the reconstructed surface layer is a promising design route to improve the OER stability.
- Numerous in situ/operando techniques have been developed throughout the entire heterogeneous electrocatalysis, e.g., in situ/operando XAS, in situ Raman spectroscopy, scanning electrochemical microscopy, in situ Fourier transform infrared spectroscopy (FTIR), in situ transmission electron microscopy (TEM), in situ X-ray photoelectron spectroscopy (XPS), in situ ultraviolet–visible spectroscopy, in situ electrochemical atomic force microscopy, femtosecond spectra, near-atmospheric scanning tunneling microscopy and isotope labeling experiments. Indeed, in situ/operando techniques could directly observe the process and achieve various data and information in real time, e.g., the adsorption behaviors, the nature of intermediates, the microstructural molecular species, the changes in active species and the reaction process pathways during OER, all of which could provide more persuasive and solid evidences to clarify the underlying OER mechanism and in turn, benefit the future designs of perovskite oxides with higher catalytic efficiency.
- Theoretical computations (e.g., DFT calculation, molecular dynamics simulation and machine learning) have been widely utilized to better rationalize the experimental results from perspectives of thermodynamics and kinetics. Specifically, DFT calculations and molecular dynamics simulations that partially involve the calculations of Gibbs free energies, adsorption energies and density of states as well as charge densities and transition states could well interpret the relationships among the intrinsically thermodynamic and/or kinetic properties and the corresponding enhanced OER catalytic performances over various materials. In recent years, artificial intelligence (AI) and machine learning (ML) have been gradually adopted to quickly discover and screen candidate materials and assist in unlocking the reaction mechanisms, including but not limited to the enumerations of optimal materials, the statistics of high-throughput experimental and theoretical parameters and the predictions of stable facets, adsorption sites and adsorption energies. The theoretical computations, possibly coupled with AI and ML, are thus highly encouraged in future research to advance the methodology and technology for constructing perovskite oxide-based materials with high OER catalytic performances.
- Catalytic descriptors can intuitively reflect the OER catalytic activities of materials and the associated catalytic mechanisms. Typically, these descriptors include ($\Delta G_{{{\mathrm{*O}}}} {{{-}}}\Delta G_{{{\mathrm{*OH}}}}$) [38], ${\mathrm{e}}_{\mathrm{g}}$ orbital filling [16], M–O covalency [43], and the position of O 2p band center relative to Fermi level [31] derived from theoretical calculations, all of which have been proposed for perovskite oxides toward OER. Based on these descriptors, much work has been accomplished and significant advances have been made in terms of designing and evaluating high-performance perovskite oxides in the past decades. However, these individual descriptors are limited within their own capacities when individually explaining and predicting the OER performances of the perovskite oxides having complicated structures, composition and undergoing sophisticated reaction mechanisms at the same time. Therefore, a universal descriptor that comprehensively combines the contributions from multiple descriptors should be established to collectively reflect the near-reality of the OER process, thus allowing more intelligent design strategies to accomplish the ultimate task of implementing the practical OER catalytic system at the commercial level.
- Considerable progress has been achieved in the development of perovskite oxides to boost OER, but quite limited studies focus on the industrial-scale synthesis of perovskite oxides to meet the practical requirements of commercialization. Almost all the reported synthetic routes are still limited to the lab-scale level, severely challenging their further utilization. Moreover, the harsh operating conditions, including high temperature (70–100 °C), high pressure (> 1 Mpa), high electrolyte concentration (> 1 mol L^−1^) for the industrial devices (e.g., proton or anion exchange membrane water electrolyzers), normally pose stringent restrictions on the selection of perovskite oxide-based catalysts. To break the current bottlenecks, exploring facile, low-cost, environment-friendly and highly reproducible synthetic routes that could meet the fundamental economical and scaling-up requirements, and simultaneously match the manufacturing technology to guarantee overall efficiency of the developed industrial devices has never been more imperative.
